# Development, validation, and pilot-testing of a comprehensive patient-reported EORTC custom questionnaire for monitoring real-world tyrosine kinase toxicity in chronic myeloid leukemia

**DOI:** 10.1007/s00520-026-11371-w

**Published:** 2026-11-12

**Authors:** Yolba Smit, Jacolien Timmerman, Alexander Currie, Hatem Soltan, Inger L. Abma, Jeroen J. W. M. Janssen, Eduardus F. M. Posthuma, Rianne Fijten, Ewald M. Bronkhorst, Andre Dekker, Rosella P. M. G. Hermens, Nicole M. A. Blijlevens

**Affiliations:** 1 Department of Haematology, Radboud university medical center, Route 476, Nijmegen, PO Box 9101, 6500HB The Netherlands; 2 IQ Health Science Department, Radboud university medical center, Nijmegen, The Netherlands; 3 Department of Internal Medicine, Reinier de Graaf Hospital, Delft, The Netherlands; 4 Department of Haematology, Leiden University Medical Center, Leiden, The Netherlands; 5 Department of Radiation Oncology (Maastro), GROW School for Oncology, Maastricht University Medical Centre+, Maastricht, the Netherlands

**Keywords:** Patient-reported outcome measures, Chronic myeloid leukemia, TKI, PROM, Toxicity monitoring, Tolerability

## Abstract

**Purpose:**

In chronic myeloid leukemia (CML), treatment choices between tyrosine kinase inhibitors (TKIs) are guided by efficacy and tolerability, yet no instrument adequately captures tolerability from the patient perspective. As a result, TKI choice may not comprehensively take into account the prevalence and severity of what patients’ experience. We therefore developed, validated, and pilot-tested a new custom questionnaire to capture real-world patients’ experiences, and explored pilot data.

**Methods:**

A mixed-methods study was conducted, following COREQ and STROBE guidelines. Development started with CML-specific items from existing instruments, followed by semi-structured sessions with purposefully sampled patients, and convenience-sampled professionals, focused on comprehensibility, comprehensiveness, relevance, acceptability, workflow, and graphics. Based on qualitative analyses, items were substituted or added from the EORTC Item library. Psychometric quantitative evaluation assessed filter question validity, redundancy, reliability, measurement error, responsiveness, scale construction, and symptom severity differences in pilot data and meta-analyses.

**Results:**

A new EORTC custom questionnaire was developed, covering 50 symptoms. Seven of those symptoms are covered by scales consisting of multiple items, with a Cronbach’s alpha ≥ 0.73 for each of those scales. Psychometric properties further showed fair-to-excellent reliability and positive agreement between time points ranging from 45 to 93% in test–retest, while responsiveness was indicated for half of symptoms. Twenty-four generic filter questions are available for computer adaptive testing, an option preferred by 87% of patients. For 23 issues, no existing EORTC item was available; these need to be added later. Pilot data showed that the severity of symptoms, as reported by patients, differed significantly between the three most common TKIs in a third of symptoms. These significant differences ranged from 5 to 30%. The highest difference was for swelling of the eyes or face, which was reported 30% more severe in patients on imatinib, compared to patients on nilotinib, when adjusted for age, gender, years since diagnosis, years on current treatment, and TKI-dosage. We could not adjust for comorbidity or comedication.

**Conclusion:**

We developed and validated an EORTC custom questionnaire and workflow to monitor 50 CML patient-reported toxicity symptoms in clinical care. Sexuality problems are excluded from pre-existing instruments, yet pilot data revealed these are prevalent and severe from patients’ perspective. One-third of evaluated symptoms varied in severity by type of TKI treatment, independent from age, gender, years since diagnosis, years on current treatment, and TKI-dosage. These findings can tentatively inform shared decision-making, when switching TKIs due to intolerability.

**Supplementary Information:**

The online version contains supplementary material available at https://doi.org/10.1007/s00520-026-11371-w.

## Introduction

Adverse event assessment in cancer treatment needs to be modernised—particularly by incorporating patient-reported outcomes in real-world settings [1–5]. This is especially relevant in cancers requiring lifelong oral targeted treatment, where multiple options exist and decisions are shaped by both efficacy and tolerability. This is the case in chronic myeloid leukemia (CML) for instance. In its chronic form—as opposed to the acute phase of CML—it can be treated with one of six commonly used oral tyrosine kinase inhibitors (TKIs). This life-saving oral TKI treatment—the life expectancy of chronic CML patients approaches that of the general population—comes at the cost of low-grade intolerability symptoms. Unfortunately, most patients need to endure these symptoms life-long, as only up to one-third of CML patients can stop their TKI treatment at some point. Though the symptoms experienced during TKI use are often referred to as ‘low-grade’, their perpetual occurrence is a great burden on patients’ daily lives: due to the intolerability of the symptoms they experience, one in four to one in five patients switches to a different TKI [6, 7].

Unfortunately, evidence on intolerability, experienced by patients as symptoms, from both randomised [8, 9] and non-randomised [10–23] studies, is scattered, inconsistent and sometimes conflicting [24]. As a result, treatment switches are often based on expert knowledge, which tends to underestimate both the prevalence and severity of what patients experience [25]. Therefore, patient-reported toxicity symptom data are essential to incorporate the patient’s perspective, provide real-world insights into treatment tolerability, and guide decision-making regarding dose modifications or treatment switches [26]. Yet, no current instrument sufficiently captures TKI-related toxicity as experienced by patients [27]. We therefore developed and validated a new custom questionnaire to allow CML patients to monitor TKI toxicity in daily clinical care, complete with a workflow and graphical overview, while evaluating between TKI differences.

## Methods

This mixed-methods study applied both qualitative and quantitative methodology, reported according to COREQ [28] and STROBE [29] (overviews in Supplementary File 1). Ethical approval was waived, as the study did not fall under the Medical Research Involving Human Subjects Act.

**Fig. 1:**
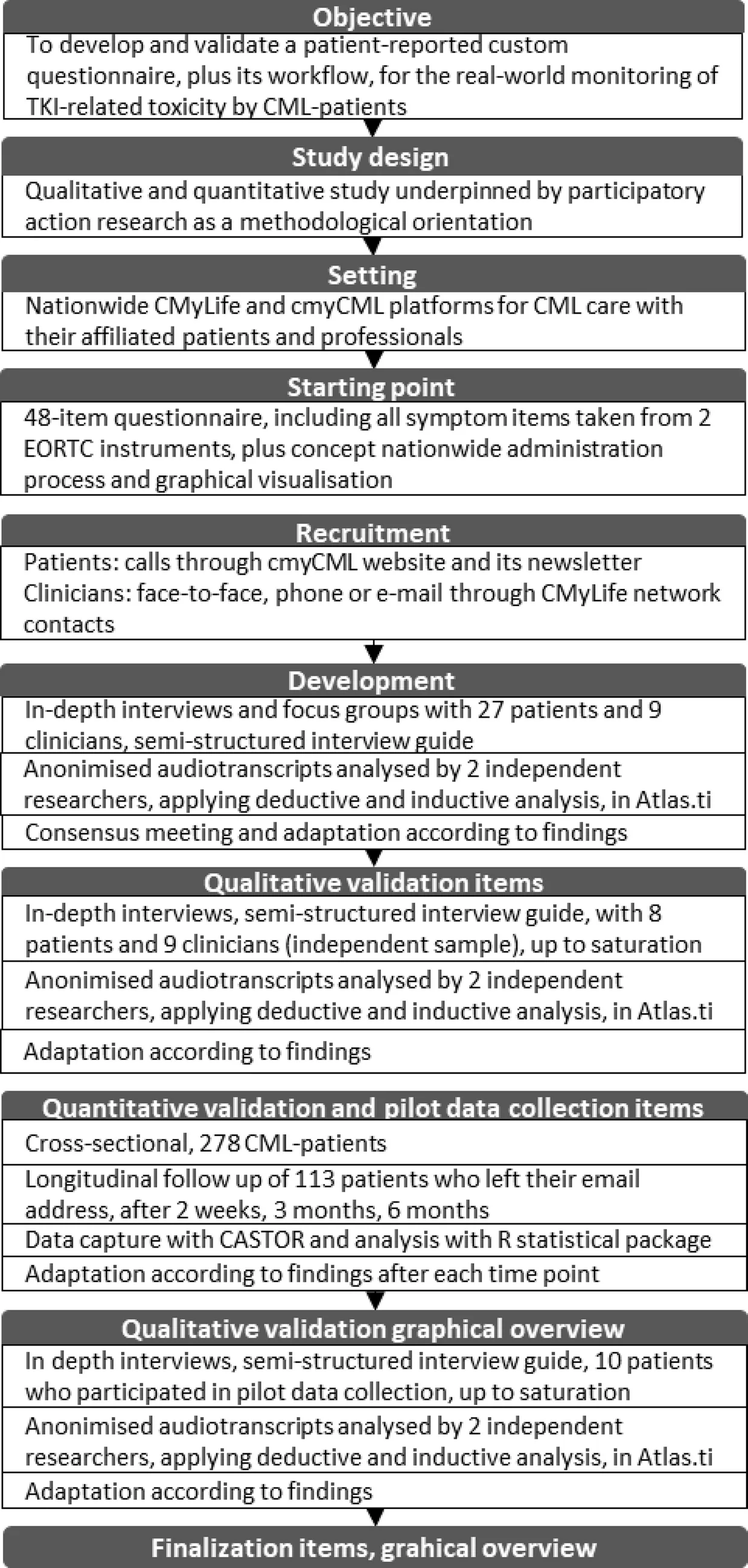
Study flow diagram

This study consisted of a development phase, followed by qualitative and quantitative validation phases, pilot data collection and analyses. Figure 1 summarises the study flow, for which we here describe the main points and in Supplementary File 1 the details. Participatory action research was the methodological orientation underpinning this study: aiming to improve daily practice through change and wanting to empower research participants in improving daily practice [30].

### Design requirements and setting

The questionnaire was designed primarily for real-world clinical use, alongside research, to capture symptoms and complaints CML patients experience from TKI therapy, enabling personalised advice aligned with current guidelines. It was intended for electronic administration via the CMyLife platform, which supports Dutch CML patients manage their CML, interpret BCR::ABL1 results, and take action. CMyLife currently includes a patient-portal (www.cmycml.nl), a CML guideline app, and a clinical decision support system [31, 32].

### Development and qualitative validation phases

The questionnaire started as a 48-item tool combining symptom items from the EORTC QLQ-CML-24 and Symptom Set [16, 33, 34], the only instruments capturing key toxicities [27]. It was expanded and refined during development and validation through interviews and focus groups (sessions) with CML patients and professionals, including nurse specialists and hematologists. The EORTC Item library—containing over 1000 items—was used throughout, along with its contracting pipeline, to add or replace items as needed [35].

### Quantitative validation phase and pilot data

We recruited chronic-phase CML patients via www.cmycml.nl for an online survey on TKI-related toxicity and feedback (T1). Respondents were invited for follow-up surveys at 2 weeks (T2), 3 months (T3), and 6 months (T4). Questionnaires, administered through Castor electronic data-capture software, were adapted between timepoints based on feedback and research needs. Pilot data from T1-4 informed scale construction to enhance interpretability for real-world research and reporting (Supplementary File 1). We evaluated feedback, filter-item validity, redundancy, item-level reliability, measurement error, responsiveness, symptom severity, and TKI differences—based on both pilot data and systematically sourced studies [24] in meta-analyses—and performed cluster analysis. Analyses were conducted in R 4.4.1; meta-analyses in STATA 18.5.

### Development and validation graphical overview

In addition to questionnaire development and validation, we developed graphical concepts to visualise symptom severity over time, based on best practices [36–38]. We subsequently held sessions—similar to those for the questionnaire—with patients and professionals to elicit preferences and validate the adapted design.

### Role of the funding source

Funding sources had no role in study design, data collection, analysis, interpretation, writing the report, or the decision to submit.

## Results

A total of at least 278 patients and 18 healthcare professionals participated in the development and validation phases, including pilot data collection, depicted in Fig. 1 and described here below in detail. Patient participants in the qualitative phases may overlap with those in the quantitative phase. Table 1 provides the patient characteristics per phase. Translated illustrative quotations and detailed analyses are provided in Supplementary File 1.

**Table 1:** Characteristics of participants in each phase

	Development^$^	Qualitative validation^$^	Quantitative validation^$,#^	Qualitative validation graphical overview^#^
Patients				
Number	27	8	278	10
Female	16 (59.3%)	6 (75.0%)	145 (52.1%)	
Median age in years (range)	60.5 y (33 to 77)	61 y (49 to 84)	63 (28 to 84)	59 (47 to 84)
Median time since diagnosis in years	5 y (0 to 16)	5 y (1 to 23)	6 (1 to 22)	7 (3 to 10)
University medical centre	9 (33.3%)	2 (25.0%)	Not available	Not available
Educational level*	Low 1 (3.7%)	Low 1 (12.5%)	Not available	Not available
	Middle 3 (11.1%)	Middle 2 (25.0%)		
	High 19 (70.4%)	High 5 (62.5%)		
	Unknown 4 (14.8%)	Unknown 0		
*Treatment:*	Not available	Not available		
Asciminib			12 (4.5%)	1 (10.0%)
Bosutinib			14 (5.2%)	1 (10.0%)
Dasatinib			70 (26.2%)	2 (20.0%)
Imatinib			84 (31.5%)	1 (10.0%)
Nilotinib			39 (14.6%)	1 (10.0%)
Ponatinib			8 (3.0%)	1 (10.0%)
Treatment-free remission			40 (15.0%)	3 (30.0%)
Nurse-specialists			Not applicable	Not applicable
Number	5	2		
Female	5 (100%)	2 (100%)		
University medical centre	2 (40%)	1 (50%)		
Hematologists			Not applicable	Not applicable
Number	4	7		
Female	1 (25%)	5 (71%)		
University medical centre	2 (50%)	4 (57%)		

^$^Participants in the qualitative validation phase were an independent sample from the development phase. Patients in qualitative and quantitative phases may have overlapped. ^#^Sampled from quantitative validation participants. *According to the definition of Statistics Netherlands

### Development phase

Twenty-seven patients, five nurse specialists (one in-training), and four hematologists participated in the development phase. Patients were purposefully sampled from 59 applicants to our online recruitment, with no refusals. We had no data on which TKI applicants were using. They took part in three online group sessions and three individual sessions (one by telephone) based on patient preference. Professionals—both nurse specialists and hematologists—participated in three individual sessions and three sessions with two attendees, all online, to accommodate their schedules. Three nurse specialists declined due to time constraints, and one did not respond.

During sessions, participants identified issues across eight topics (Supplementary File 1): Comprehensibility, Comprehensiveness, (Personal) Relevance, Frequency of administration, Order of items, Open text-box, Recall period, and Graphical concepts. In response to the first topic Comprehensibility, one item on ‘pain in chest’ was replaced by ‘pain on chest’ to better reflect common Dutch phrasing. In response to the second topic Comprehensiveness, thirteen items were added to the 48 items we started the development phase with. In addition, patients identified seven missing issues which had no corresponding items in the EORTC Item library (Table 2).

**Table 2:**
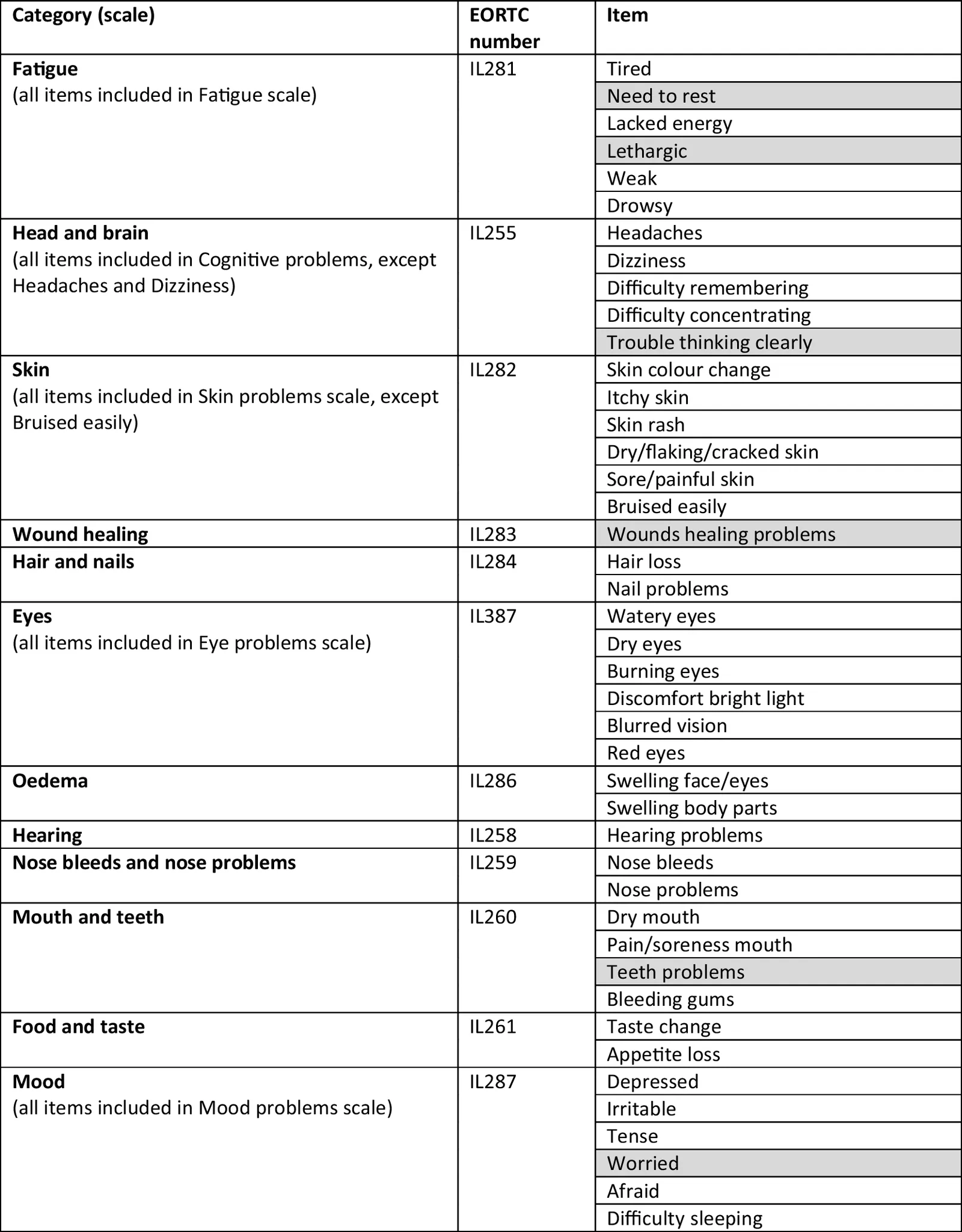

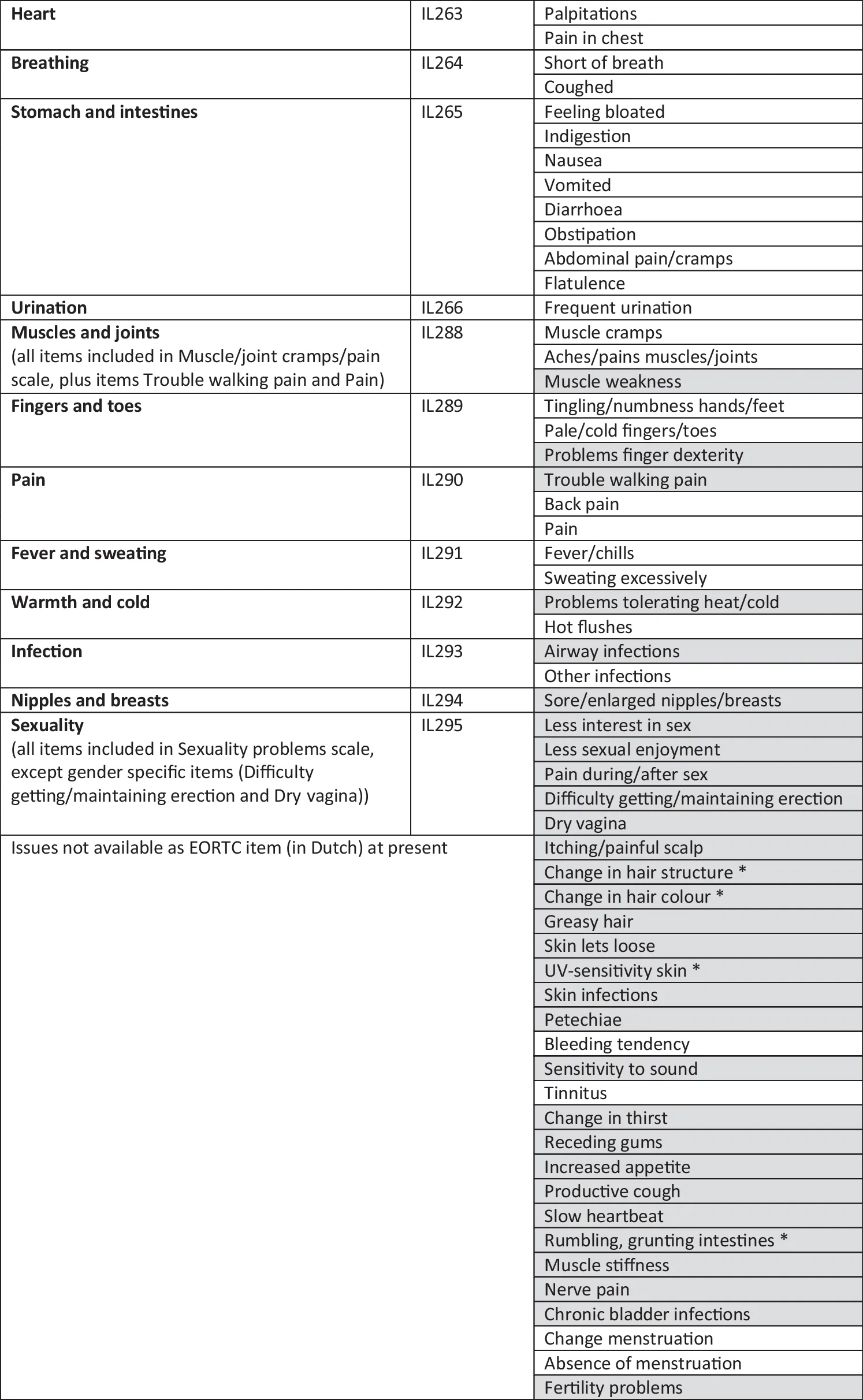
The final custom questionnaires^$^ with category names, items pertaining to categories and scales, and missing issues

Items in shaded cells are not included in any other currently available, validated questionnaire [27]

^$^Also available as one questionnaire (EORTC IL386) or as an add-on to the QLQ-CML-24 (EORTC IL407) with 49 symptom items additional to the QLQ-CML24

*Not available in Dutch language

Regarding the third topic which came up during sessions, (Personal) Relevance, patients preferred not to classify items as irrelevant and valued comprehensiveness over brevity. Professionals considered issues outside of their expertise or unamendable to be less relevant, though no items were deemed irrelevant. All participants were interested in how to tailor the questionnaire to make it a more personalised experience. Examples mentioned were as follows: a more ample use of filter questions, only administer items which previously scored either ‘a little’, ‘quite a bit’, or ‘very much’, and: enable to decline administration with a simple: ‘I have no symptoms’.

Four perspectives emerged with regard to the fourth topic raised during session: Frequency of administration. These four perspectives were: link the administration to BCR::ABL1 monitoring, administer more frequently at the start of (a new) TKI, also administer during treatment-free remission, and enable on-demand administration on patients’ own initiative. Concerning the fifth topic raised during sessions, Order of items, participants preferred to keep similar items together, start with the most prevalent symptoms, and move from physical items first to emotional items later on. In addition, an open text-box was the sixth topic raised, and was deemed useful for entering any missing symptom or lifestyle factors that might relate to symptoms was requested by several patients. One patient mentioned in the chat of an online group session that the Recall period of a week is rather short, and this was considered the seventh topic raised during sessions. No more information was available on the reason, or for which items the recall period was considered too short.

With regard to graphical concepts, which was the last topic raised during sessions, participants were asked to objectively interpret both bar and line concept graphs depicting the course of symptoms over time. Almost all interpretations were correct, both for bar as well as for line graphs. Patients preferred a line graph to a bar graph. For the symptom overview, participants preferred an overview with multiple line graphs, requested addition of TKI type and dosage, preferred clustering of symptoms in logical groupings for better oversight, and wanted to be able to share their symptom overview with professionals.

### Qualitative validation phase

Eight patients, two nurse specialists and seven hematologists, all of whom had not participated in the development phase, participated in sessions on the qualitative validation phase. We had no data on which TKI applicants were using. Two lower educated [39] patients were recruited through nurse specialists, because very little lower educated patients applied via the online recruitment. Six patients were purposefully sampled from 59 applicants to our online recruitment call, with no refusals. All patients were interviewed individually: two by telephone at their request and the other six online. Professionals took part in seven individual sessions and one session with two professionals, all online and so planned for convenience to their schedules. Participants raised new issues related to Comprehensibility and Comprehensiveness (Supplementary File 1). Twelve items were added and one item, considered unclear because of the phrasing ‘Have you had problems with coughing?’, was replaced by ‘Have you coughed?’. For eight issues that patients identified as missing, no corresponding items were available in the EORTC Item library (Table 2). No new issues emerged in the last two interviews after the sixth interview, indicating saturation.

### Quantitative validation phase

#### Response, feedback

In the quantitative validation phase, we invited chronic phase CML patients to fill in the questionnaire and answer questions about their user experience, through the CMyCML website (www.cmycml.nl) and its newsletter. At T1, 278 patients responded, out of an unknown number of patients viewing the CMyCML website or its newsletter, and all were included. Imatinib was used by 31.5%, dasatinib by 26.2%, 15.0% were in treatment-free remission, 14.6% were on nilotinib, 5.2% on bosutinib, 4.5% on asciminib, and 3.0% on ponatinib (Table 1). One hundred thirteen (40.6%) opted to complete subsequent surveys, with response rates of 78.8% (T2), 75.5% (T3), and 71.7% (T4). On average, patients took 8.0 min (SD 4.4) to complete 72 items in a single questionnaire (T1), compared to 9.4 min (SD 3.8 min) to complete 17 filter questions, leading to 17 questionnaires with a maximum of 77 items (T3). Most patients (86.8%) preferred completing multiple questionnaires with filter questions, 10.8% preferred a single questionnaire with all items, and 2.4% preferred answering only the items that had bothered them in the previous administration.

When asked about their opinion on the questionnaire, overall, patients’ attitude was positive; most patients found it easy to complete (63.2%) or were neutral (12.9%); 94.5% stated they had understood all items; 47.7% found that the questionnaire gave them insight into their complaints and symptoms; and only 1.4% found it stressful to fill in. 59.5% of patients found it better to divide the questionnaire into categories, to more easily find questions that would apply to them. At T1, a minority of patients (14.0%) said they would not use the questionnaire to get an overview of their symptoms or complaints, whereas 44.7% would, and 41.3% was neutral. Patients who would not use the questionnaire to get an overview, stated as reasons: not wanting to be occupied with their disease, not seeing added value, as symptoms were known to them, not having (a lot of) symptoms, and difficulty in interpreting their symptoms as TKI- or otherwise related. Patients preferred a median of 75 days (IQR, 148.8) between filling out surveys. At T2, 56% of patients indicated they preferred an open text-box at the end of the questionnaire, to either register other symptoms or complaints, give extra or more nuanced information, e.g. on co-morbidities, co-medication, social functioning, quality of life, or pose questions. Patients gave as new feedback that they had comorbidities or used comedications that might affect their answers. At T3, all patients understood all items. No new feedback was received at T4.

Based on patients’ feedback, seven items were added and one item (about taste) was replaced for better comprehensibility. However, no corresponding items were available in the EORTC Item library for eight missing issues (Table 2, details in Supplementary File 1).

#### Psychometric properties

We evaluated the *Validity of filter questions* for symptom categories (Methods in Supplementary File 1), because of the wish for a more personalised experience, and a preference for symptom categories. Filter questions were considered the most feasible approach to achieve this. Two overarching EORTC-items (skin problems, eye problems) were invalid as filters for more detailed items, and would not suffice for the questionnaire as a whole. Therefore, custom generic filter questions were developed and tested for item categories during T3-T4, resulting in 24 filter questions. Of these, 22 were deemed valid at T4, while two (on ‘Nose bleeds and nose problems’, and ‘Sexuality’) were considered invalid, with adapted versions available for further field testing in the future (Supplementary File 1). Table 2 shows the final instrument, available as a single 77-item EORTC custom questionnaire or 24 separate EORTC custom questionnaires with 24 generic filter questions, plus 23 missing issues that need to be added later. *Redundancy*: no inter-item correlation exceeded 0.9 for any item combination (data not shown), so all items were retained. *Reliability* and *measurement error* were evaluated for the 70 items administered at T1 and T2 (test–retest). All items had at least fair-to-good reliability (weighted kappa ≥ 0.40), though the lower 95%CI was below 0.40 for 22 items. Fourteen items showed excellent reliability (weighted kappa ≥ 0.75). The percentage positive agreement between T1 and T2 ranged from 45.3% for dry, flaky, or cracked skin to 93.3% for Sore/enlarged nipples/breasts (Supplementary File 1). Indications for sufficient *responsiveness* were found for 21/50 symptoms/scales (see Scale construction under Pilot data). The median change from T3 to T4 was 29.6% in self-reported improved patients, 7.7% in stable patients, and −18.5 in deteriorated patients (Supplementary File 1).

#### Graphical overview

**Fig. 2:**
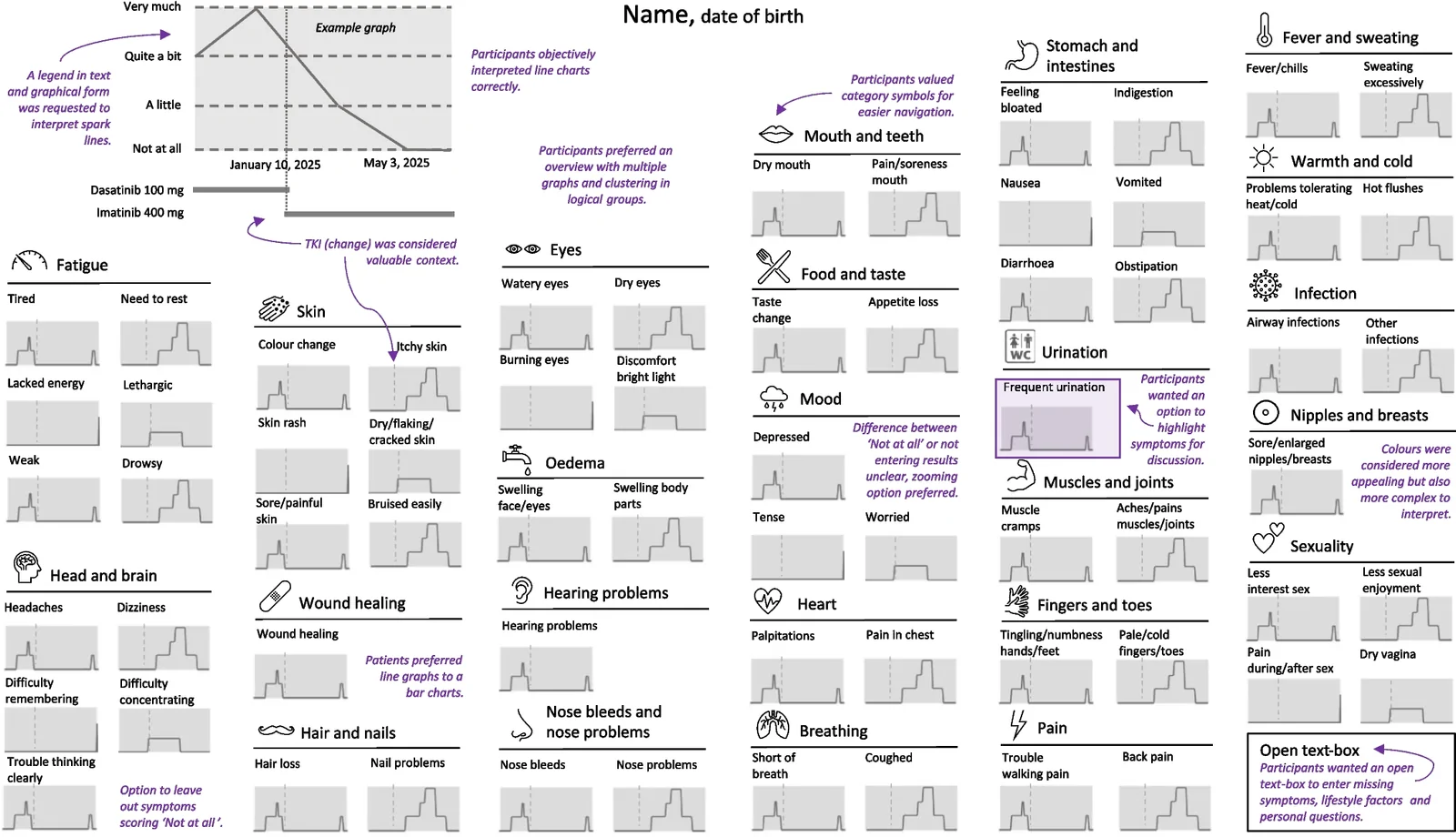
The final graphical overview, with issues participants brought forward in the development and validation phases in purple

Ten patients participated in sessions on the graphical overview; four invited participants did not respond, and one declined. All interviews were online except one requested as in-person. No new issues emerged after the eighth interview, indicating saturation. Feedback included legend, category symbols, medication change, highlight symptoms, colour design, zoom option, and omitting ‘Not at all’ symptoms (Fig. 2). To address questionnaire length and the resulting cluttered graphics, several solutions were proposed and implemented: (1) clustering symptoms in logical categories, with symbols for easier navigation; (2) option to hide ‘Not at all’ results; (3) ability to highlight symptoms for discussion in consultation, avoiding time spent on less relevant issues.

### Pilot data

*Scale construction and characteristics*: 71 out of 77 items were retained for factor analysis, resulting in 7 scales with 33 items in total. Each of these seven scales had a meaningful interpretation and good internal consistency (Cronbach’s alpha ≥ 0.73, Supplementary File 1). Therefore, pilot data analyses concerned 7 scales and 43 single items, totalling 50 symptoms/scales. *(Comparative) analyses*: Fatigue was both the most prevalent and severe symptom across all TKIs (summarised in Table 3, detailed in Supplementary File 1). Other common issues included Muscle/joint cramps/pain, Eye problems, Skin problems, Mood problems, Cognitive problems, and Sexuality problems, as well as Flatulence, Frequent urination, and Tingling/numbness of hands/feet. At T1, the adjusted mean severity of 13 of 44 evaluable symptoms/scales (29.5%) differed significantly across treatment options with ≥ 30 participants (Table 4). However, only Swelling face/eyes and Feeling bloated showed robust significant differences after correction for multiple comparisons. *Meta-analyses*: Eight studies [11, 14–20] contributed data for meta-analyses of eight individual symptoms (Supplementary File 1). For three symptoms—Nausea/vomiting, Dyspnoea and Appetite loss—indirect and unadjusted comparisons indicated statistically significant differences between TKI treatments, in addition to the differences observed with our pilot data alone. *Cluster analysis*: The clustering separated patients with less severe symptoms from those with more severe symptoms. Clusters differed significantly by gender, with a higher proportion of females in the higher severity cluster (*p* = 0.030). No significant differences were found for other covariates, though there was a trend suggesting a more recent diagnosis was associated with the more severe symptom cluster (*p* = 0.051).

**Table 3:** Most prevalent and severe symptoms per current treatment

Current CML-treatment	Prevalence			Severity		
	*N* of symptoms/scales which bothered ≥ 50% of patients	Top 5	Prevalence (95% confidence interval)	*N* of symptoms/scales with mean symptom severity > 33.3	Top 5	Mean severity (95% confidence interval)
Any TKI	12	Fatigue (scale) Eye problems (scale) Muscle/joint cramps/pain (scale) Skin problems (scale) Mood problems (scale)	91.6 (87.2–94.9) 86.6 (80.9–91.2) 85.0 (78.9 (89.9) 83.1 (76.9–88.1) 80.5 (74.3 (85.8)	2	Fatigue (scale) Flatulence Frequent urination Muscle/joint cramps/pain (scale) Dry vagina	41.5 (38.2–44.8) 36.3 (31.5–41.0) 31.6 (26.9–36.4) 28.6 (25.2–31.9) 27.9 (21.0–34.8)
Asciminib	31	Muscle/joint cramps/pain (scale) Fatigue (scale) Skin problems (scale) Mood problems (scale) Cognitive problems (scale)	100 (63.1–100) 91.7 (61.5–99.8) 91.7 (61.5–99.8) 90.0 (55.5–99.7) 88.9 (51.8–99.7)	10	Feeling bloated Short of breath Coughed Abdominal pain/cramps Fatigue (scale)	45.8 (3.9–87.8) 45.8 (16.3–75.4) 45.8 (16.3–75.4) 41.6 (9.2–74.1) 40.3 (23.8–56.7)
Bosutinib	24	Fatigue (scale) Flatulence Muscle/joint cramps/pain (scale) Cognitive problems (scale) Skin problems (scale) Eye problems (scale) Mood problems (scale)	100 (76.8–100) 100 (69.2–100) 81.8 (48.2–97.7) 75.0 (34.9–96.8) 75.0 (34.9–96.8) 75.0 (34.9–96.8) 75.0 (34.9–96.8)	9	Flatulence Nose problems Abdominal pain/cramps Fatigue (scale) Feeling bloated	70.0 (49.1–90.9) 46.6 (0–100) 43.3 (9.5–77.1) 42.8 (29.0–56.6) 42.4 (15.7–69.1)
Dasatinib	17	Fatigue (scale) Skin problems (scale) Mood problems (scale) Eye problems (scale) Muscle/joint cramps/pain (scale)	91.4 (82.3–96.8) 85.7 (73.8–93.6) 84.7 (73.0–92.8) 81.8 (69.1–90.9) 81.8 (69.1–90.9)	5	Fatigue (scale) Pale/cold fingers toes Frequent urination Tingling/numbness hands/feet Flatulence	46.3 (39.8–52.9) 40.0 (20.7–59.3) 35.7 (26.1–45.4) 35.1 (25.3–45.0) 34.5 (25.4–43.7)
Imatinib	16	Muscle/joint cramps/pain (scale) Fatigue (scale) Eye problems (scale) Skin problems (scale) Mood problems (scale)	89.7 (79.9–95.8) 89.3 (80.6–95.0) 88.7 (79.0–95.0) 78.9 (67.6–87.7) 78.7 (67.7–87.3)	3	Fatigue (scale) Swelling face/eyes Flatulence Muscle/joint cramps/pain (scale) Diarrhoea	39.0 (33.7–44.3) 38.9 (31.3–46.4) 36.7 (29.5–44.0) 31.3 (25.7–36.9) 30.9 (22.3–39.5)
Nilotinib	11	Fatigue (scale) Eye problems (scale) Skin problems (scale) Mood problems (scale) Muscle/joint cramps/pain (scale)	94.9 (82.7–99.4) 91.2 (76.3–98.1) 82.9 (66.4–93.4) 78.4 (61.8–90.2) 75.0 (56.6–88.5)	2	Difficult erection Fatigue (scale) Dry vagina Flatulence Back pain	41.6 (19.3–64.0) 38.5 (31.2–45.7) 33.3 (16.2–50.5) 32.3 (21.1–43.5) 30.9 (10.3–51.6)
Ponatinib	15	Fatigue (scale) Cognitive problems (scale) Eye problems (scale) Dry mouth Indigestion Frequent urination Tingling/numbness hands/feet	87.5 (47.3–99.7) 85.7 (42.1–99.6) 85.7 (42.1–99.6) 85.7 (42.1–99.6) 83.3 (35.9–99.6) 83.3 (35.9–99.6) 83.3 (35.9–99.6)	2	Fatigue (scale) Frequent urination Nose problems Dry mouth Feeling bloated	40.5 (20.2–60.9) 38.9 (12.5–65.2) 33.3 33.3 (15.5–51.1) 33.3 (2.0–64.6)
Treatment-free remission	13	Muscle/joint cramps/pain (scale) Fatigue (scale) Mood problems (scale) Skin problems (scale) Nose problems	93.8 (79.2–99.2) 90.0 (76.3–97.2) 83.3 (67.2–93.6) 82.9 (66.4–93.4) 75.0 (34.9–96.8)	3	Fatigue (scale) Back pain Dry vagina Muscle/joint cramps/pain (scale) Nose problems	40.5 (32.3–48.8) 37.5 (6.1–68.8) 35.5 (15.3–55.8) 30.9 (24.0–37.8) 29.1 (11.3–47.0)

Severity ranges from 0 (‘not at all’) to 100 (‘a lot’), with a score of 33.3 indicating ‘a little’, and 66.6 ‘quite a bit’. No 95% confidence interval calculated if only one score available, or if all scores were the same

**Table 4:**
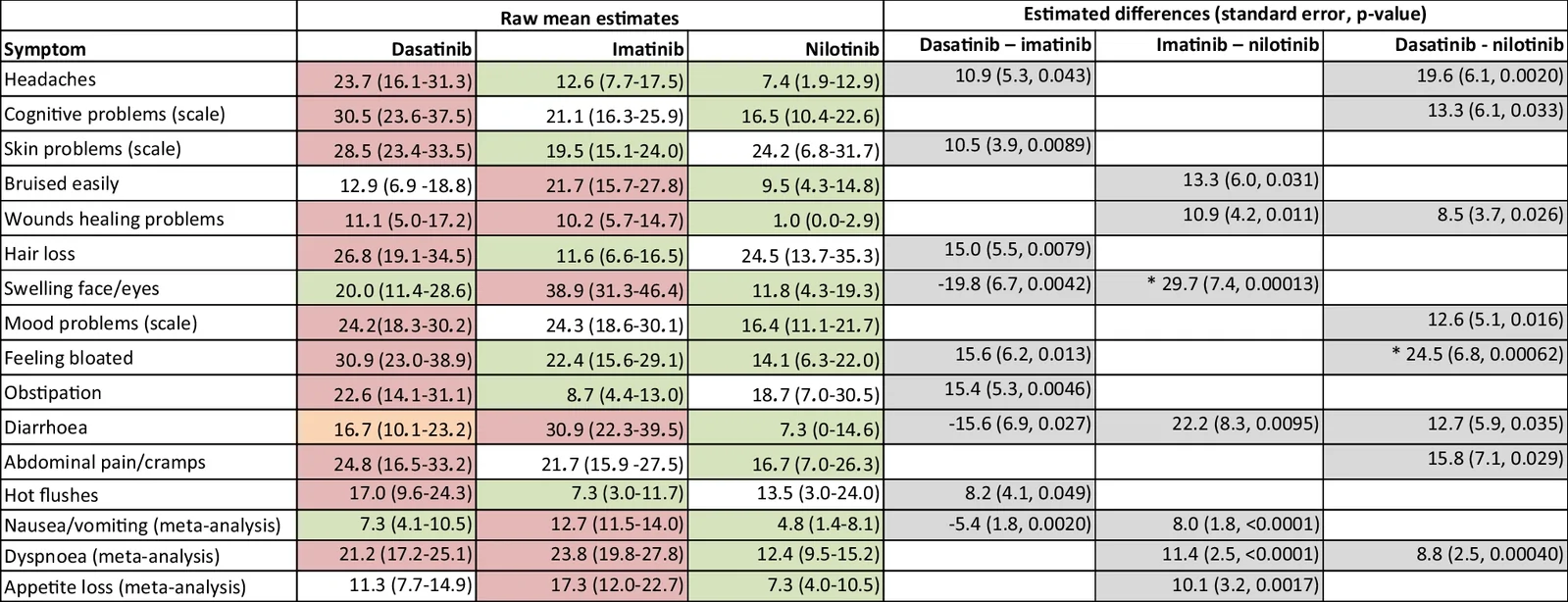
Symptoms with a significant difference between current TKI treatments, used by ≥ 30 patients, from our pilot data (top 13 rows) and meta-analyses (bottom three rows) of eight systematically identified studies [11, 14–20] and our pilot data

*Robust in Benjamini–Hochberg procedure for multiple comparisons; Symptom scores range from 0 (‘not at all’) to 100 (‘a lot’), with a score of 33.3 indicating ‘a little’, and 66.6 ‘quite a bit’. Shaded areas indicate a covariant-adjusted (age, gender, years since diagnosis, years on current treatment, TKI-dosage) or meta-analysed significant difference between treatment options, with red indicating a worse symptom experience; green a better symptom experience; orange an intermediate symptom experience; and grey an adjusted (pilot data) or unadjusted (meta-analysis) estimate of significant differences

## Discussion

In this study, we developed, validated, and pilot-tested a questionnaire to measure CML patient-reported toxicity in real-world settings, including its workflow and graphical feedback. The questionnaire is available through the EORTC Item library as a single questionnaire (IL386), an add-on to QLQ-CML24 (IL407), or as 24 separate questionnaires with filter questions – the latter preferred by most patients. Compared to existing instruments [27], it adds 17 items, notably on sexuality, which ranked among the most prevalent and severe symptoms in our pilot data, which is not reflected in product information brochures [40–45]. This highlights the need for research on sexual health in CML treatment, and in haematology in general [46, 47].

On 23 issues, no items exist in the EORTC Item library—the most comprehensive library available—indicating future inclusion needs. Menstrual disorders lack items despite patient demand and their listing as side effects in dasatinib, imatinib, and nilotinib brochures [42, 43, 45]. Updates will be needed as new TKIs emerge; few patients used asciminib, bosutinib, or ponatinib. Patients favoured an open text-box for additional details, enabling detection of emerging toxicities. Reliability was fair to excellent; responsiveness was shown for nearly half the scales, but others lacked sufficient data or were possibly affected by recall period or anchor questions. Designing responsiveness studies remains challenging without a gold standard. Importantly, comprehensibility—a prerequisite for responsiveness—was validated.

Pilot data indicated both a high prevalence and high severity of symptoms. Patients with more severe symptoms may have been more likely to participate, introducing selection bias. However, out of eight symptoms/scales reported elsewhere, mean severity was comparable for six. Only fatigue during dasatinib and nilotinib, and insomnia during imatinib were more severe in our study. Meta-analysis was largely impossible because most published data lacked individual symptom details. The higher proportion of females in the high-severity cluster aligns with previous findings of more severe symptoms in female CML patients [16] and broader sex differences in adverse drug reactions [48]. Possible explanations include a relatively higher (pharmacological) TKI dose in women, who generally have lower body mass, and other sex/gender factors such as physiology, lifestyle, behaviour, help-seeking, communication, or adherence. If women experience more severe symptoms, this may explain why they comprised 52.1% of respondents, despite CML being more common in men (ratio 1.5 [49]). Ultimately, sex- and gender-specific TKI prescribing may be justified, as treatment response disparities are increasingly recognised in other fields [50]. Our custom questionnaire could support more equitable care.

In our pilot data and meta-analyses of systematically sourced studies, one-third of evaluated symptoms/scales differed significantly in severity across most common treatments, with differences up to 30%. These findings can guide shared decision-making when switching TKIs is indicated. Differences aligned with expected pathophysiology: dasatinib, which showed the most severe symptoms in most cases, has more off-tumour on-target effects than imatinib and nilotinib [51]. More severe bruising, wound-healing issues, and facial/eye swelling with imatinib may relate to its effects on endothelial permeability, and impeded thrombus formation and collagen synthesis [52].

This raises the broader question of whether patient-reported symptoms are causally linked to current TKI treatment. Using a validated instrument does not imply causality, just as clinician-reported adverse events do not [5, 53]. Comorbidities, comedications, prior dosages, previous TKIs, and depth of response may all influence symptoms. Patients in our pilot data also noted difficulty attributing causes. For example, high severity of Difficulty in getting or maintaining an erection in nilotinib users may reflect age, comorbidities, or cardiovascular risk management rather than the drug itself. Still, knowing patients experience these symptoms is important – regardless of causality – to enable discussion, facilitate shared decision-making, and prompt action [54].

Literature shows CML patients on TKIs report worse scores for depression, dyspnoea, fatigue, insomnia, cognitive issues, pain, physical health-related quality of life, and composite symptom burden (e.g. nausea, diarrhoea, itching, skin changes, swelling) compared to the general population or controls [17, 55, 56]. However, by TKI, such data are often unavailable for most symptoms. A nationwide cohort study TKI-treated CML patients had an increased risk for 142 of 670 disease categories – including ophthalmic, infectious, ear, and brain disorders – with none more common in controls [57]. Thus, evidence on experienced symptoms remains limited.

A major strength of this study is patients’ and professional involvement – not only in questionnaire development and validation, but also in providing results and workflow feedback. While rigorous guidance exists for selecting and assessing patient-reported outcome measures [58], to our knowledge, no such guidance addresses presenting results or workflow design.

Beyond missing clinical data (e.g. prior TKIs), a key limitation is the small size of some pilot subgroups, which hindered comparisons with newer TKIs, dosage effects, and treatment changes (switching, stopping, adjusting). We may have lacked power to detect differences between the three most common TKIs for certain symptom-TKI combinations, especially sex-dependent symptoms such as erectile dysfunction, the most severe symptom in nilotinib users. For example, detecting a 10% difference in fatigue between imatinib and other TKIs would require 97 patients per group, versus 44 for a 15% difference. Only imatinib and dasatinib groups exceeded 44 participants. Achieving such sizes is challenging in this rare disease, especially when accounting for various treatment pathways and prior TKI use. This underscores the need for large, preferably nationwide studies linking patient-reported outcomes to clinical data.

Beyond TKI differences, large prospective or longitudinal studies – such as RODEO [59] – are needed to assess how dose reductions affect symptom severity, in which patients, and to what extent. Such evidence is crucial for personalising TKI strategies [54]. The interim results of RODEO showed modest but significant improvements (5–10%) in diarrhoea, fatigue, and nausea/vomiting across TKIs [60].

## Conclusions

We developed and validated an EORTC custom questionnaire and workflow to monitor CML patient-reported toxicity in clinical care. Pilot data revealed a broader symptom spectrum than existing tools and showed one-third of symptoms varied in severity by TKI-type, independent from age, gender, years since diagnosis, years on current treatment, and TKI-dosage. However, we could not evaluate our findings independent from comorbidities and comedication, or in the newer TKIs. Our findings can tentatively inform shared decision-making when switching TKIs due to intolerability. Developing and validating patient-reported instruments for real-world use is iterative, especially with emerging therapies. Future studies should therefore confirm our findings and explore newer TKIs, dose adjustments, treatment switches, comorbidities and comedication, to fully leverage patient-reported toxicity data in clinical practice.

## Supplementary Information

Below is the link to the electronic supplementary material.

ESM 1 Supplementary Material 1 (DOCX 1.44 MB)

## Data Availability

The datasets are published in Data Sharing Collections in the Radboud Data Repository (https://doi.org/10.34973/9288-ds63) under restricted access. Requests should be directed at the corresponding author and will be reviewed by the Committee of Healthcare Renewal and Innovation of the Dutch Society for Hematology. Data will be shared with researchers who provide a sound protocol and sign a data access agreement. Approved requests will be granted immediate access upon publication of this article, and for up to 15 years thereafter.
