# Supplementary material for: Development, validation, and pilot-testing of a comprehensive patient-reported EORTC custom questionnaire for monitoring real-world tyrosine kinase toxicity in chronic myeloid leukemia
Source: Support Care Cancer. 2026 Nov 12;34(12):1220. doi: 10.1007/s00520-026-11371-w (PMC13759593; doi:10.1007/s00520-026-11371-w)
Supplement: Supplementary file 1 [file 520_2026_11371_MOESM1_ESM.docx]

**Supplementary Information for ‘Development, validation, and pilot-testing of a comprehensive patient-reported EORTC custom questionnaire for monitoring real-world tyrosine kinase toxicity in chronic myeloid leukemia’ Supportive Care in Cancer**

Yolba Smit, Jacolien Timmerman, Alexander Currie, Hatem Soltan, Inger Abma, Jeroen Janssen, Eduardus Posthuma, Rianne Fijten, Ewald Bronkhorst, Andre Dekker, Rosella Hermens, Nicole Blijlevens

Mrs. Yolba Smit, Department of Hematology, Radboud university medical center, [yolba.smit@radboudumc.nl](mailto:yolba.smit@radboudumc.nl)

Contents

[COREQ and STROBE checklists	3](#_Toc208919188)

[Methods	6](#_Toc208919189)

[Development phase	6](#_Toc208919190)

[Study population and recruitment	6](#_Toc208919191)

[Sessions	6](#_Toc208919192)

[Qualitative data analysis	6](#_Toc208919193)

[Consensus meeting	6](#_Toc208919194)

[Qualitative validation phase of the custom questionnaire	6](#_Toc208919195)

[Quantitative validation phase	7](#_Toc208919196)

[Recruitment and surveys	7](#_Toc208919197)

[Analyses	7](#_Toc208919198)

[Pilot-data	7](#_Toc208919199)

[Qualitative validation of the graphical overview	8](#_Toc208919200)

[Topics semi-structured interview guides	8](#_Toc208919201)

[Development	8](#_Toc208919202)

[Validation	8](#_Toc208919203)

[IL234	10](#_Toc208919204)

[Detailed results development phase	10](#_Toc208919205)

[Consensus meeting	11](#_Toc208919206)

[Detailed results qualitative validation	16](#_Toc208919207)

[Detailed results quantitative validation	20](#_Toc208919208)

[Final set of filter questions	21](#_Toc208919209)

[Reliability and measurement error	22](#_Toc208919210)

[Responsiveness	23](#_Toc208919211)

[Prevalence of symptom score categories	25](#_Toc208919212)

[Mean and median symptom scores	41](#_Toc208919213)

[Our pilot data in the context of published literature	47](#_Toc208919214)

[Meta-analysis of severity of symptoms	47](#_Toc208919215)

[TKI-treatments compared in other studies	58](#_Toc208919216)

[References	60](#_Toc208919217)

# COREQ and STROBE checklists

Table 1 COREQ (COnsolidated criteria for REporting Qualitative research) Checklist [1]

| Topic | Item no. | Guide questions/description | This study |
| --- | --- | --- | --- |
| Domain 1: Research team and reﬂexivity | | | |
| Personal characteristics | | | |
| Interviewer/facilitator | 1 | Which author/s conducted the interview or focus group? | Yolba Smit, Alexander Currie |
| Credentials | 2 | What were the researcher’s credentials? E.g. PhD, MD | YS: MD MSc; AC: BM |
| Occupation | 3 | What was their occupation at the time of the study? | YS: Policy advisor, PhD student; AC: Medical student |
| Gender | 4 | Was the researcher male or female? | YS: Female; AC: Male |
| Experience and training | 5 | What experience or training did the researcher have? | YS: Trained as a medical doctor and MSc in epidemiology. AC: Trained as a medical doctor, half-way Master phase. Both: Received recent training in conducting focus groups and individual interviews for qualitative research. |
| Relationship with participants | | | |
| Relationship established | 6 | Was a relationship established prior to study commencement? | YS: knew two patients, one nurse-specialist and 5 hematologists/guideline developers form previous collaborations. One of the guideline developer/hematologists is the promotor in her PhD trajectory. AC: no prior relationship |
| Participant knowledge of the interviewer | 7 | What did the participants know about the researcher? e.g. personal  goals, reasons for doing the research | All participants were made aware of the PhD position of YS and of the research questions. |
| Interviewer characteristics | 8 | What characteristics were reported about the inter viewer/facilitator? e.g. Bias, assumptions, reasons and interests in the research topic | YS’s main interest is applied guideline development. The focus of her PhD is to evaluate how patients’ experiences can best be incorporated in to the guideline development process in a systematic way. She assumes that guidelines in general can benefit to some extent from capturing patients experiences, such as side effects, systematically. |
| Domain 2: Study design | | | Study protocols: [Zenya WebShare - Radboudumc](https://webshare.zenya.work/j18l7prkd4ctjmly/DocumentList.aspx) |
| Theoretical framework | | | |
| Methodological orientation and Theory | 9 | What methodological orientation was stated to underpin the study? e.g. grounded theory, discourse analysis, ethnography, phenomenology, content analysis | Participatory action research was the methodological orientation that underpinned this study. Participatory action research aims to understand reality, as well as also improve it through change, and in doing so wants to empower the research participants [2]. |
| Participant selection | | | |
| Sampling | 10 | How were participants selected? e.g. purposive, convenience, consecutive, snowball | See Methods “Study population and recruitment” |
| Method of approach | 11 | How were participants approached? e.g. face-to-face, telephone, mail, email | See Methods “Study population and recruitment” |
| Sample size | 12 | How many participants were in the study? | See Results, Table 1 |
| Non-participation | 13 | How many people refused to participate or dropped out? Reasons? | See Results |
| Setting | | | |
| Setting of data collection | 14 | Where was the data collected? e.g. home, clinic, workplace | See Results |
| Presence of non-  participants | 15 | Was anyone else present besides the participants and researchers? | A facilitator from the CMyLife team was present during all focus groups, and at two focus groups and some interviews with professionals an apprentice was present |
| Description of sample | 16 | What are the important characteristics of the sample? e.g. demographic data, date | See Results Table 1 |
| Data collection | | | |
| Interview guide | 17 | Were questions, prompts, guides provided by the authors? Was it pilot tested? | See Methods “Sessions”, and Interview guides in Supplementary Information |
| Repeat interviews | 18 | Were repeat inter views carried out? If yes, how many? | No |
| Audio/visual recording | 19 | Did the research use audio or visual recording to collect the data? | See Methods “Sessions” |
| Field notes | 20 | Were ﬁeld notes made during and/or after the interview or focus group? | Field notes were made during and/or after sessions |
| Duration | 21 | What was the duration of the inter views or focus group? | See Results |
| Data saturation | 22 | Was data saturation discussed? | See Methods “Qualitative data analysis” |
| Transcripts returned | 23 | Were transcripts returned to participants for comment and/or correction? | No |
| Domain 3: analysis and ﬁndings | | | |
| Data analysis | | | |
| Number of data coders | 24 | How many data coders coded the data? | Two |
| Description of the coding tree | 25 | Did authors provide a description of the coding tree? | See Supplementary Information ‘Detailed results development phase’, with tables, and similar for the qualitative validation phase |
| Derivation of themes | 26 | Were themes identiﬁed in advance or derived from the data? | See Methods “Data analysis” |
| Software | 27 | What software, if applicable, was used to manage the data? | See Methods “Data analysis” |
| Participant checking | 28 | Did participants provide feedback on the ﬁndings? | No |
| Reporting | | | |
| Quotations presented | 29 | Were participant quotations presented to illustrate the themes/ﬁndings? Was each quotation identiﬁed? e.g. participant number | See Supplementary Information ‘Detailed results development phase’, with tables, and similar for the qualitative validation phase |
| Data and ﬁndings consistent | 30 | Was there consistency between the data presented and the ﬁndings? | Results section |
| Clarity of major themes | 31 | Were major themes clearly presented in the ﬁndings? | Table 2, Results section |
| Clarity of minor themes | 32 | Is there a description of diverse cases or discussion of minor themes? | Results and Discussion sections, plus detailed description in Supplementary Information |

Table 2 STROBE (Strengthening the Reporting of Observational Studies in Epidemiology) checklist [3] for quantitative validation and pilot data analyses

| Topic | Item  No | Recommendation | This study |
| --- | --- | --- | --- |
| Title and abstract | | | |
|  | 1 | (a) Indicate the study’s design with a commonly used term in the title or the abstract | Abstract |
|  |  | (b) Provide in the abstract an informative and balanced summary of what was done and what was found | Abstract |
| Introduction | | | |
| Background/  rationale | 2 | Explain the scientific background and rationale for the investigation being reported | Introduction |
| Objectives | 3 | State specific objectives, including any prespecified hypotheses | Introduction |
| Methods | | | |
| Study design | 4 | Present key elements of study design early in the paper | See Methods. Study protocols: [Zenya WebShare - Radboudumc](https://webshare.zenya.work/j18l7prkd4ctjmly/DocumentList.aspx) |
| Setting | 5 | Describe the setting, locations, and relevant dates, including periods of recruitment, exposure, follow-up, and data collection | Described in: Quantitative validation, Recruitment and surveys |
| Participants | 6 | (a) Cohort study—Give the eligibility criteria, and the sources and methods of selection of participants. Describe methods of follow-up  Cross-sectional study—Give the eligibility criteria, and the sources and methods of selection of participants | Described in: Quantitative validation, Recruitment and surveys |
| Variables | 7 | Clearly define all outcomes, exposures, predictors, potential confounders, and effect modifiers. Give diagnostic criteria, if applicable | Described in: Quantitative validation paragraph |
| Data sources/  measurement | 8* | For each variable of interest, give sources of data and details of methods of assessment (measurement). Describe comparability of assessment methods if there is more than one group | Described in: Quantitative validation paragraph |
| Bias | 9 | Describe any efforts to address potential sources of bias | None |
| Study size | 10 | Explain how the study size was arrived at | The objective was to include as many participants as possible |
| Quantitative variables | 11 | Explain how quantitative variables were handled in the analyses. If applicable, describe which groupings were chosen and why | Described in: Analyses quantitative validation, and Pilot data paragraphs |
| Statistical methods | 12 | (a) Describe all statistical methods, including those used to control for confounding | Described in: Analyses quantitative validation, and Pilot data paragraphs |
|  |  | (b) Describe any methods used to examine subgroups and interactions |  |
|  |  | (c) Explain how missing data were addressed |  |
|  |  | (d) Cohort study—If applicable, explain how loss to follow-up was addressed  Cross-sectional study—If applicable, describe analytical methods taking account of sampling strategy |  |
|  |  | (e) Describe any sensitivity analyses |  |
| Results | | | |
| Participants | 13* | (a) Report numbers of individuals at each stage of study—e.g. numbers potentially eligible, examined for eligibility, confirmed eligible, included in the study, completing follow-up, and analysed | Results section, Paragraph Quantitative validation, response, feedback |
|  |  | (b) Give reasons for non-participation at each stage | Unknown |
|  |  | (c) Consider use of a flow diagram | Figure 1 |
| Descriptive data | 14* | (a) Give characteristics of study participants (e.g. demographic, clinical, social) and information on exposures and potential confounders | Table 1 |
|  |  | (b) Indicate number of participants with missing data for each variable of interest | Not reported |
|  |  | (c) *Cohort study*—Summarise follow-up time (e.g., average and total amount) | 6 months from t1 to T4 |
| Outcome data | 15* | *Cohort study*—Report numbers of outcome events or summary measures over time | Results section and Supplementary Information |
|  |  | *Cross-sectional study—*Report numbers of outcome events or summary measures | Results section and Supplementary Information |
| Main results | 16 | (a) Give unadjusted estimates and, if applicable, confounder-adjusted estimates and their precision (e.g., 95% confidence interval). Make clear which confounders were adjusted for and why they were included | Results section, table 4, 5 and Supplementary Information |
|  |  | (*b*) Report category boundaries when continuous variables were categorised | Methods section |
|  |  | (*c*) If relevant, consider translating estimates of relative risk into absolute risk for a meaningful time period | Not relevant |
| Other analyses | 17 | Report other analyses done—e.g. analyses of subgroups and interactions, and sensitivity analyses | No other analyses done |
| Discussion | | | |
| Key results | 18 | Summarise key results with reference to study objectives | Discussion section |
| Limitations | 19 | Discuss limitations of the study, taking into account sources of potential bias or imprecision  Discuss both direction and magnitude of any potential bias |  |
| Interpretation | 20 | Give a cautious overall interpretation of results considering objectives, limitations, multiplicity of analyses, results from similar studies, and other relevant evidence |  |
| Generalizability | 21 | Discuss the generalisability (external validity) of the study results |  |
| Other information | | | |
| Funding | 22 | Give the source of funding and the role of the funders for the present study and, if applicable, for the original study on which the present article is based | Declaration of interest paragraph |

*Give information separately for cases and controls in case-control studies and, if applicable, for exposed and unexposed groups in cohort and cross-sectional studies

# Methods

## Development phase

### Study population and recruitment

We advertised for adult chronic-phase CML patients willing to participate in online video or telephone interviews, either individually or in groups, hereafter referred to as sessions, through [www.cmycml.nl](http://www.cmycml.nl). We stated we wanted to discuss TKI-related toxicity monitoring. Fifty-nine applicants were purposefully sampled to reflect differences in sex, age, time since diagnosis, education, and hospital type where treatment took place. To address the low number of low-educated applicants, we also recruited through the hematologists involved in this research (NB, JJ, EP).

We recruited hematology nurse specialists (in training) and hematologists working with CML patients though convenience sampling (until saturation) via the CMyLife network. We prioritised CML nurse specialists, assuming they discuss toxicity symptoms more in more detail than hematologists, whose primary focus is disease control. Once the CML nurse specialist pool was exhausted we began inviting hematologists. We invited nine nurse specialists and four hematologists in person, by phone, email, or through their professional society.

### Sessions

All sessions were conducted online due to initial COVID restrictions and later for convenience, though telephone or live interviews were offered as alternatives. Initially, we preferred patient focus groups, assuming that interaction would provide valuable insights. However, after three focus groups, we switched to individual interviews, as nurse specialists and hematologists suggested that sexual symptoms were relevant and better discussed in one-on-one sessions.

Two researchers (YS, AC) conducted all sessions using semi-structured interview guides (appendix p 7), focusing on comprehensibility, comprehensiveness, and relevance as main focal points for content validity [4], and acceptability, workflow and graphic concepts. The core team developed the guides, and the patient version was pilot-tested on a patient representative. For the item-list and workflow, participants received the test questionnaire 2-5 days prior to the session—patients were asked to complete it, and professionals to review it. Participants were informed that the questionnaire was intended for routine use within the CMyLife-platform. Informed consent was obtained prior to sessions, which were audio-recorded and transcribed verbatim, anonymizing data. Transcripts were not returned for comments or corrections.

### Qualitative data analysis

Two researchers (either YS, JT, AC and/or HS) independently coded text fragments using inductive coding in Atlas.ti, reaching consensus through discussion. Pre-set topics were comprehensibility, comprehensiveness, relevance, workflow and graphic concepts. Codes were categorised into pre-set topics or new topics as needed. Saturation could not be assessed in the development phase due to the focus group format.

### Consensus meeting

Major design decisions were made in a consensus meeting with involvement of patient representatives, individual patients, hematologists, researchers and the CMyLife team. The consensus meeting was held right after the development phase, with the goal to consolidate proposals– proposals that originated from participants perspectives in the qualitative analysis - and lay down the principles for future decision making. Participants of the consensus meeting were a patient representative, four patients, three hematologists, and five team members (researchers and members of the CMyLife team). Patients and hematologists had also participated in the development phase.

## Qualitative validation phase of the custom questionnaire

Study population and recruitment, sessions and qualitative data analysis were similar to the methodology of the development phase, with the exception of saturation. In the validation phase, saturation was defined as no new topics emerging in the last two sessions after the sixth session, with assessment and discussion after each session. We chose two sessions after the sixth session to start saturation assessment, as it is unlikely saturation is reached within fewer than eight sessions.

## Quantitative validation phase

### Recruitment and surveys

At **T1**, we administered the EORTC IL234 (appendix p 8) along with feedback questions on completion time, preferred timing, willingness to use, questionnaire attitude, comprehensiveness, comprehensibility, and other feedback. At **T2**, patients completed the IL234 and provided open-text feedback. At **T3**, we administered 17 questionnaires (EORTC IL254-270) along with 17 filter questions to assess whether a questionnaire applied. In addition, feedback was requested on completion time, questionnaire and filter-questions attitude, comprehensiveness and comprehensibility, and other feedback. At **T4**, 24 questionnaires (EORTC IL255, 258-261, 263-266, 281-295) were administered, along with 24 filter questions to assess whether a questionnaire applied, an anchoring question for each item (see responsiveness below), and feedback questions.

### Analyses

Missing data were excluded. Response proportions and feedback were analysed descriptively. **Scale construction**: We constructed symptom scales post hoc using T1 data and exploratory parallel factor analysis with oblimin rotation. Analysis was performed if the Kaiser-Meyer-Olkin test of sampling adequacy was ≥0.5 and Bartlett’s test of sphericity p<0.05. Items were excluded if they had only low (<0.32) or high (>0.90) inter-item correlations, low prevalence (<10%), or were gender-specific. Scales (factors) were retained and iteratively constructed based on a combination of: eigen value>1, scree plot patterns, interpretability, and factor loadings >0.30. Internal consistency was assessed using Cronbach’s alpha, with values >0.70 [5] considered acceptable. **Validity filter-items**: at T1, we evaluated the use of two overarching EORTC items, for skin and eye problems, as filter-questions. They were considered valid if ≤5% of patients who responded ‘not at all’ or ‘a little bit’ to the overarching item responded ‘quite a bit’ or ‘very much’ on any corresponding detailed item – indicating that patients interpreted the overarching item as inclusive of its sub-items. At T3, we used custom filter questions. These were considered preliminarily valid if <10% of patients who reported no symptoms in a given category at T3 had reported ‘quite a bit’ or ‘very much’ for any relevant item at T1. At T4, we revised the custom filter questions and administered all detailed items to every patients, regardless of their response to the filter question. Validity was assessed by comparing responses to each filter question with its corresponding items, using the same threshold as at T3. **Redundancy** was defined as a high inter-item Spearman’s rank correlation (>0.9) [5], and such items were considered for removal. **Item-level reliability** was assessed using weighted kappa, comparing T1-T2 scores for patients on the same TKI and dosage. We assumed that if symptoms were TKI-related, reliable items would yield consistent responses. A weighted kappa ≥0.40 was considered fair to good and ≥0.75 excellent [5]. **Measurement error** was defined as the percentage positive agreement between T1-T2 scores in patients on the same TKI and dosage. In the absence of a gold standard, **responsiveness** was assessed using self-reported change via item-specific anchor questions (‘Is that different from three months ago?’). If the Spearman’s rank correlation between self-reported change and mean symptom severity change from T3 to T4 was acceptable (≥0.30 [6]), we compared changes across patients who reported ‘improvement’, ‘stability’ or ‘deterioration’ using the Kruskal-Wallis rank-sum test.

## Pilot-data

**(Comparative) analyses**: missing items in scales were ignored if ≥50% of items were available, as all inter-total correlations exceeded 0.47. Item and scales scores were linearly transformed [7]. We described symptom prevalence and severity, and assessed differences in mean scores across current treatments with >30 patients using linear models adjusted for age, gender, year of diagnosis, time on current TKI, and TKI dosage. Data from T1 were chosen, as T1 provided the most respondents, except for six items introduced at T4 (Pale/cold fingers/toes; Nail problems; Other nose problems; Back pain; Airway infections; and Other infections). TKI dosage was categorised as below norm or at/above norm. Norm-dosages (milligram/day) were: asciminib 80, bosutinib 400, dasatinib 100, imatinib 400, nilotinib 600, and ponatinib 45. Analyses were exploratory and tested for robustness to multiple comparisons using the Benjamini-Hochberg procedure [8]. We conducted random-effects **meta-analyses** of mean individual symptom severities, comparing TKI subgroups using both our pilot data and systematically identified studies that used EORTC instruments in ≥30 patients treated with dasatinib, imatinib or nilotinib. **Cluster analysis**: To create a simplified indicator for toxicity symptoms we applied K-means clustering. To this end symptom severity scores for all points in time were used. To be able to apply K-means clustering missing values had to be dealt with. Symptoms only applicable for one gender were imputed with a score of 0 for the other gender. For six symptoms the fraction missing was very high (>75%), these were excluded from the cluster analyses. This concerned Nose problems, Back pain, Pale cold skin, Nails, Airway infections and Other infections. For all other symptoms, missing values were imputated using the mice package (v3.17.0), using all measurements as predictors for the missing values. Applying the silhouette procedure, two clusters were found to be optimal (Packages cluster (v2.1.8) and factorextra (v1.0.7)).

### Qualitative validation of the graphical overview

To validate the graphical overview, we purposefully sampled patients who participated in the longitudinal quantitative validation, ensuring diversity in age, gender, current treatment, and missing answers. These patients were invited by email. Patients received their own longitudinal data before the interview.

# Topics semi-structured interview guides

Each session, and each topic within a session, ended with an open question for other feedback, and whether participants wanted to raise additional issues. Prompts used were: Could you elaborate?; Could you give me an example?; How do you feel about … ? What needs to be different/left out/added?

## Development

Acceptability, comprehensibility, comprehensiveness, relevance

- Feedback on introduction of questionnaire
- Time needed for completion, opinion on number of items, type of questions
- Comprehensibility (patients only)
- Were there questions you preferred not to answer? (patients only)
- Order of questions
- Missing questions
- Relevance of questions
- Willingness to complete questionnaire (patients only)
- Open text field

Administration, accessibility and privacy

- Opinion on administration in app, feedback on mock-up screens app
- Frequency of administration
- Patient-types to administer to
- Preferences for invites and reminders (patients only)
- Other languages
- Out-of-app administration, low health literacy
- Privacy concerns

Graphical concepts

- Preferences for information in overview, graph types
- Feedback on multiple prototypes (design, scoring (thinking aloud method), legend, sharing)
- Interpretation: check of different prototype mock-up graphs (change over time, in relation to TKI-dosage)

## Validation

Acceptability, comprehensibility, comprehensiveness, relevance

- Feedback on introduction of questionnaire
- Time needed for completion, opinion on number of items, type of questions
- Comprehensibility (patients only)
- Order of questions, missing questions
- Willingness to complete questionnaire (patients only)
- Open text field

Graphical concept symptoms overview (own data, patients only)

- Feedback on concept, design, also per item (legend, graphs, icons, layout)
- Comprehensibility, comprehensiveness, perceived usefulness

# IL234

Table 3 The 72 items included in the EORTC IL234, with potential filter items in dark blue, items pertaining to potential EORTC filter questions in light blue, and the 25 items added during the development and qualitative validation phases in light grey

| Tiredness | Burning eyes | Vomiting |
| --- | --- | --- |
| Needing to rest | Discomfort eyes bright light | Diarrhoea |
| Lacking energy | Blurred vision | Constipation |
| Lethargy | Red eyes | Abdominal pains/cramps |
| Weakness | Hearing problems | Problems gas/flatulence |
| Drowsiness | Nose bleeds | Frequent urination |
| Trouble sleeping | Dry mouth | Muscle cramps |
| Headaches | Pain/soreness mouth | Aches/pains muscles/joints |
| Dizziness | Teeth problems | Muscle weakness |
| Difficulty remembering | Bleeding gums | Trouble walking pain |
| Difficulty concentrating | Different taste food/drink | Tingling/numbness hands/feet |
| Trouble thinking clear | Lack appetite | Difficulty small objects |
| Easy bruising | Depressed | Swelling body parts |
| Skin problems | Irritable | Pain |
| Skin colour change | Tense | Fevers/chills |
| Itchy skin | Worrying | Excessive sweating |
| Skin rash | Afraid | Problems tolerating heat/cold |
| Dry/flaking/cracked skin | Palpitations | Hot flushes |
| Sore/painful skin | Pain in chest | Sore/enlarged nipples/breasts |
| Wound healing problems | Shortness of breath | Less interest sex |
| Hair loss | Coughing | Less sexual enjoyment |
| Swelling face/eyes | Bloated abdomen | Pain during/after sexual activity |
| Eye problems | Acid indigestion/heartburn | Difficulty getting/maintaining erection |
| Watery eyes | Nausea | Dry vagina |

# Detailed results development phase

Themes that arose from the perspectives of participants with regard to the development of the questionnaire were: (1) Comprehensibility; (2) Comprehensiveness; (3) (Personal) relevance; (4) Frequency of administration; (5) Order of items; (6) Open text-box; (7) Recall period; and (8) Graphical concepts. There were no issues with the instructions. Five items had issues with **(1) Comprehensibility** (Table 4): two items were retained; one item was added; one alternative item was chosen. The first item was retained because, though patients did not understand a Flemish Dutch word, the comprehension of the item was not affected; for one item there was no alternative in the EORTC Item library but an additional item was added to be tested in the validation phase; for one item an alternative was chosen; two items were retained as screening items though participants indicated that the meaning was vague to them. Regarding **(2) Comprehensiveness**, 18 topics were raised (Table 5). For 8 topics suitable items were available in the EORTC Item Library, for which 12 items were added. Sexuality was considered a missing topic by professionals, who suggested to add four screening items. None of the patient participants directly acknowledged sexuality as a missing topic. For eight topics no suitable items were available in the EORTC Item library. One missing topic was weight change, however, professionals indicated that they would prefer to have the actual weight of patients. Lastly, the missing topic of tooth pain was considered covered by the overarching pain item. With regard to **(3) (Personal) relevance**, most patients were enthusiastic and considered the list appropriate, though some patients and professionals mentioned it was a (too) long list (further reported on in the Results, and in detail in Table 6). With regard to the **(4) Frequency of administration**, perspectives differed again (further reported on in the Results, and in detail in Table 7). Some patients would like to fill in symptoms on a weekly base, while others found a once-a-year administration sufficient. Concerning the **(5) Order of items**, this is reported on in the Results, and in detail in Table 8. Another topic that patients came up with was that an **(6) Open text-box**, reported on in the Results, and in detail in Table 9. Patients mentioned in the chat that the **(7) Recall period** of a week is rather short. With regard to the **(8) Graphical concepts**, this is reported on in the Results.

# Consensus meeting

The design decisions made/principles laid down were:

- Comprehensibility:
  - Choose items from the EORTC Item Library with a more clear phrasing, if comprehensibility or user experience is affected
- Comprehensiveness:
  - Add items identified as missing, for which a suitable item is available in the EORTC Item library
  - Do not develop new items because of time and budget constraints, instead check the EORTC Item Library yearly
  - Add the actual weight of patients in the electronic administration in some way
- (Personal) relevance:
  - Comprehensiveness is considered more important than brevity by patients and therefore no items will be left out at this stage
  - Future evaluation on the prevalence of items and patient participation may alter this decision
  - Quantitative evaluation might indicate which items are not discriminative enough and can be left out
  - Enable to decline invitation to administration, with reason for decline
  - Investigate options for personalisation e.g. (more) filter questions as several EORTC items are included already (validate these in quantitatively) or categories with filter questions, or only administer items scored on previously (computer adaptive testing)
- Frequency of administration:
  - Link administration to BCR::ABL1 monitoring
  - Administer more frequently at the start of a (new) TKI
  - Also administer during treatment-free remission
  - Enable on-demand administration on patients’ own initiative, and enable reminders set by patients
- Order of items:
  - Put most prevalent symptoms first
  - Keep similar items together
- Open text-box:
  - Enable open text-box at the end
- Graphical representation:
  - Line graph is preferred option for individual graphs
  - All items in separate line graphs in the overview, per category, is the preferred option for the overview
  - Enable graph and open text-box sharing with clinicians

Table 4 Comprehensibility issues raised in the development phase, with illustrative quotes, rationale and decisive action

|  | **Issue** | **Illustrative quote** | **Action** | **Rationale** |
| --- | --- | --- | --- | --- |
| 1 | Flemish term for acid indigestion possibly not understood by patients | [indigestion] yes, a lot of people don't know what that is. They know about heartburn, but I think a lot of people think: I have no idea [Pr] | Item retained | Word ‘indigestion’ not Netherlands Dutch. Comprehensibility item by patients not affected; change if suitable alternative is available |
| 2 | Swelling unclear in Dutch, fluid retention preferred | ‘Have you had swelling in a certain part of the body’, I think it is very difficult for people to say, yes, what do we mean by that? [Pr]  Uhm, and I thought fluid retention might be a better term [Pr] | Item retained and more specific item on swelling around eyes/in face added | Swelling is very general in Dutch and not necessarily associated with oedema. Fluid retention would be a better phrase. No suitable alternative item available, but specific item on swelling of eyes and face available |
| 3 | “Pain on chest’ is common in Dutch, not ‘in chest’ | […] but I would call that pain on the chest […] not in the chest [Pr] | Alternative item chosen | Better suitable alternative available |
| 4 | ‘Worrying’ had a vague meaning to professionals | "Are you worried?" I don't really know what that means, because about what or how or what? Yes, of course it is a questionnaire and not qualitative, yes, I understand that, but, uhm, yes, I thought that was vague [Pr] | Left in as a screening item | No more specific item available |
| 5 | ‘Hearing problems’ too general | Problems with hearing, I thought that was a bit general. I think you have to give people something to think about: "I hear that, or I have that, or I find that annoying" [P] | Left in as a screening item | No more specific item available |

Abbreviations: P: patient; Pr: professional

Table 5 Comprehensiveness issues raised in the development phase, with illustrative quotes, rationale and decisive action

|  | **Issue** | **Illustrative quote** | **Action** | **Rationale** |
| --- | --- | --- | --- | --- |
| 1 | Dental pain | But perhaps also dental problems that could be involved […] toothache [P] | None | Considered covered by item pain in general |
| 2 | Bleeding tendency | My blood is thinner so I bleed worse [Pa] | Check yearly | No suitable item available |
| 3 | Change thirst | […] whether people would drink more yes or no […] People also tend to be more thirsty or less thirsty [P] | Check yearly | No suitable item available |
| 4 | Facial mask | […] because with imatinib, for example, I also have that uhm that I sometimes had a very red mask on my face. Uhm, how should I say that, uhm, during exertion or a stressful event, my whole face became really bright and bright red, everything. And that continued for a while until it happened again, until I was alone and calmed down. And then it was over again [P] | Check yearly | No suitable item available |
| 5 | Fertility problems | See sexual dysfunction | Check yearly | No suitable item available |
| 6 | Itching/painful scalp | I regularly hear from patients about itchy scalp. Now I saw that itching was mentioned, as in total, itchy skin. But I don't think people think about the scalp so quickly [Pr]  But yes, my hair is not just hair loss. I think, well, that it could also be that people have pain complaints in their scalp. […] I also know that when I moved my hair back and forth, it was really annoying [P] | Check yearly | No suitable item available |
| 7 | Skin lets loose | Skin lets loose [P, comment in chat of videoconferencing software] | Check yearly | No suitable item available |
| 8 | Sun sensitivity | I miss uh the question about sensitivity to sunlight and UV uh with TKIs. For example, I suffer from this very much. […] I can hardly ... I hardly ever sit in the sun but ... if I do and it's intense then uh, uh I immediately get a rash [P] | Check yearly | No suitable item available in Dutch |
| 9 | Tinnitus | Well, I had tinnitus, among other things. […] I am currently suffering from tinnitus [P] | Check yearly | No suitable item available |
| 10 | Weight change | It is weight loss but also gain. That's why I also ... it is not just fluid, but it is certainly also that women often gain a lot of weight [Pr] | None | Professionals prefer actual weight |
| 11 | Bloated stomach | I also suffer a lot from a swollen stomach  […] a bloated feeling, it is really full [P] | Added | Suitable item available |
| 12 | Cough | Yes, for me I miss coughing as a complaint [P] | Added | Suitable item available |
| 13 | Unintentional flatulence | And uh what I also suffer from is uh flatulence. Uh yes, I'm just going to be very honest and that is really, uh, a side effect that also makes your social contacts very difficult because I just can't stop it, that's real, and it comes at a time when you just don't expect it , just like that, out of the blue [P] | Added | Suitable item available |
| 14 | Mental capacity | what I also miss is a reduced ability to think - it is slightly different from concentration: at the end of the day it is not possible to do something difficult [P] | Added | Suitable item available |
| 15 | Sensitivity cold/heat | I would like to know […] whether anyone also suffers from cold, cold legs and cold arms? […] not being able to withstand cold and not being able to withstand heat [P]  Different temperature (hot-cold) [P, in chat] | 2 items added | Suitable item available |
| 16 | Stamina/strength | Could your fitness level be added [...] Hey, how do you experience your condition at the moment, 'can you climb the stairs without a break?' or uh uh I also wrote something like ‘can you just carry out your daily activities or do you have to skip there’? [Pr] | Added | Item on muscle weakness added, to be checked in validation phase |
| 17 | Tingling hands/feet | Tingling and pricking in toes, hands, feet and fingers and lower legs, really, uh, so bad that it caused me a lot of uh pain [P] | Added | Suitable item available |
| 18 | Sexual dysfunction | What I really miss, especially about complaints that they want to discuss, uh, which is of course always a difficult complaint, is sexual dysfunction. Uh, I definitely see erection and libido disorders with the TKIs. Just like fertility problems are an issue and that is not reflected in this list. While that is also a very complicated subject, one that many people have to talk about [Pr] | 4 Items added | Suitable items available |

Abbreviations: P: patient; Pr: professional

Table 6 Relevance issues raised in the development phase, with illustrative quotes, rationale and decisive action

| **Issue** | **Illustrative quote** | **Action** | **Rationale** |
| --- | --- | --- | --- |
| Appropriate but long list | I just think this is a good list. I have seen a list in the past and I really had my doubts whether they were focused on CML. […]Well, I don't think so with this one. I just think this is a good list [P]  In that respect, I think it is very nice and good that such a list exists. Because then you think, hey, I'm not the only one who has had this problem [P]  And I think it's a nice list, but I notice that I think it's a lot, a big list [P]  I really thought it was a lot, yes, I had it, I also showed it to the doctor yesterday and he also said: this is really way too much [Pr]  So I thought it was quite a lot of questions [Pr] | See below | No items stood out as irrelevant. Also, many participants wished to add missing items |
| Patients value comprehensiveness over brevity | Better too many than too few [P]  I wouldn't mind answering 50 questions, right? You can, it's yes or no, right? I mean, you don't have to answer them all in detail. It's a short moment of ‘yes, I have it’ or, ‘I don't have it’. You don't have to explain it. […] I would prefer completeness [P] | Be as comprehensive as possible | Patients’ wish for comprehensiveness outweighs wish for brevity |
| Personalisation is desired | […] break down into those questions. So indeed what you do with skin problems, and what you do with eyes for example, yes [Pr]  A first question might be uh, do you have any side effects? And then yes or no. Because there are people and they really don't have any problems. And if they answer no, then the rest is not necessary, right? [Pr]  And you can indicate this in advance, but you can of course also do it afterwards. That you say, well, that doesn't apply at all, and this one doesn't apply, well then uh you just keep what applies to you [P] | Evaluate possibilities for personalization:   - Allow to opt out (with reason) - Apply (more) filter questions - Apply computer adaptive testing to administer items scored on previously | Participants express wish for personalization of list, for better user experience |

Abbreviations: P: patient; Pr: professional

Table 7 Frequency of administration issues raised in the development phase, with illustrative quotes, rationale and decisive action

| **Issue** | **Illustrative quote** | **Action** | **Rationale** |
| --- | --- | --- | --- |
| Link administration to BCR::ABL1 monitoring | I might do it at the same time as the BCR-ABL check [Pr]  That's minimal actually, right? That is practical and you can relate it to an objective measure. Disease measure [BCR::ABL1] [Pr]  I would actually find it quite useful if the patient had completed such a questionnaire standardly, every time, before coming to see me [Pr] | Link administration to BCR::ABL1 monitoring |  |
| Administer more frequently at start (new) TKI | Because you have people who can't tolerate anything, and I then put them on a new drug, hey, an unregistered drug, asciminib for example, and then I would also really like to see how that works, how much impact it has [Pr]  I'm still a bit clinging to those 6 months, uhm, where, look, when you're just starting something, yes, you speak to each other more often than every 3 months, right? But for the first year, you could do it every 3 months, and then every six months.[…] because it is precisely in that first year that it is most often you switch and all that, people have side effects [Pr] | Administer more frequently at start (new) TKI | Times around starting, switching and stopping are the most interesting |
| Administer during treatment-free remission | If someone stops, for example, I thought it would be interesting to see that [toxicity symptom monitoring] more often, because people can then get those withdrawal complaints [Pr] | Administer during treatment-free remission | See above |
| Enable on-demand administration on patients’ own initiative | I [would like to] fill it out every month [P]  I would fill it out once a month [P]  Complete 1 x per month or 1 x every 2 months (not more often) [P]  Once a week is fine [P] | Enable on-demand administration on patients’ own initiative | Some patients wish to enter toxicity symptoms very frequently |

Abbreviations: P: patient; Pr: professional

Table 8 Order of items issues raised in the development phase, with illustrative quotes, rationale and decisive action

| **Issue** | **Illustrative quote** | **Action** | **Rationale** |
| --- | --- | --- | --- |
| Keep similar items together | Perhaps because I am currently in training, I miss a logical order, um, because in my view it is completely mixed up. And I think it would be better if you did it in order of the tract anamnesis [Pr] | Reorder and check in validation phase | Some participants felt order of items was not logical (enough) |
| Start with most prevalent symptoms | I can also imagine that you want to do it in order of likelihood of complaints. Uhm, even then I think it's not quite in the right order  I know myself, if I have to answer 48 questions, and the first 20 don't apply to me, then I don't feel like it anymore, then I drop out. Then I'm really not going to finish it. And if I start with the questions that I think are recognizable, recognizable, I have that too, then I am more motivated to finish it. So hence my suggestion to start with uh with the common points [P] | Reorder and check in validation phase | Starting with most prevalent symptoms ensures better user experience |
| Move from physical to emotional items | Sequence […] from physical to emotional [P] | Reorder and check in validation phase | The questionnaire will start with the most prevalent symptoms (fatigue) and keep emotional symptoms such as mood symptoms somewhere in the middle to stress their importance. In the consensus meeting it was preferred that sexuality items would be moved to the middle as well, not to leave them as the last stop during consultations. However, EORTC recommended against as the recall period for most sexuality items is four weeks vs. one week for all other items. If inserted at the end the recall period changes only once |

Abbreviations: P: patient; Pr: professional

Table 9 Open text-box issue raised in the development phase, with illustrative quotes, rationale and decisive action

| **Issue** | **Illustrative quote** | **Action** | **Rationale** |
| --- | --- | --- | --- |
| Provide an open text-box | I missed something when filling it out, but I can't remember that now. So open field would be nice [P]  Perhaps you can add your own question and answer [P]  I would really like to see […] why I had a cramp in my thigh on Thursday evening at seven o'clock. And I want to be able to read that […] on that Thursday evening […] I made a lot of physical effort, which caused the cramp […] I must say that […] what you did that day and the side effect you have might be related. Then you cannot necessarily say that it could be due to the medicines. So then you actually rule out some things [P] | Provide an open text field at the end | Patients indicated they wanted to enter missing symptoms, questions and/or answers, and lifestyle factors that might relate to (the severity of) symptoms |

Abbreviations: P: patient; Pr: professional

Table 10 Recall period issue raised in the development phase, with illustrative quotes, rationale and decisive action

| **Issue** | **Illustrative quote** | **Action** | **Rationale** |
| --- | --- | --- | --- |
| A week is short to look back | I think last week is so short. Might do for a longer time [P]  A week [is] short to look back [P] | None | A week is a generally accepted recall period, also used with the PRO-CTCAE for example |

Abbreviations: P: patient; Pr: professional

# Detailed results qualitative validation

New issues were mentioned within the topics: (1) Comprehensibility; (2) Comprehensiveness; and (3) Graphical overview. A summary of all items added, and the final set after the qualitative validation phase is shown in Table 3. Twelve items had issues with **(1)** **Comprehensibility** (Table 11): eleven items were retained; and one item was switched for a better suitable alternative. Of the eleven retained items, six did not have a better suitable alternative available in the EORTC Item Library, though for one item an additional option was available that might be useful and this item was added. Of the other five items, one item was an overarching item which captured both suggestions of the participant; one item was to be moved to a better suited cluster of items; one item included both complicated and simple phrasing in Dutch and was therefore considered understandable; one item with a double negative phrasing was phrased in that sense to keep all answering options in the same direction (a higher score indicates more symptoms); one item was considered a milder version of the suggestion done by the participant. Regarding **(2) Comprehensiveness**, 21 new topics were raised (Table 12). For 11 topics suitable items were available in the EORTC Item Library, for which 11 items were added. For eight topics no suitable items were available in the EORTC Item library. Two topics were considered interference items, and were not added due to the many items already included. With regard to the **(3) Graphical overview**, patients discussed their own graphical overview: some text was redundant and could be left out; explanation in text and graphical form was required to be able to understand the spark lines; category symbols contributed to easier navigation; inclusion of medication (change) was valuable context; option to highlight symptoms for discussion with professionals would give focus; difference between stable scores and not scoring not visible in spark lines so zooming option is preferred; though colours were perceived as more appealing, they add complexity in design and interpretation; opinions differed on whether to leave out symptoms that only scored ‘not at all’ so this may be a personalised option.

Table 11 New comprehensibility issues raised in the development phase, with illustrative quotes, rationale and decisive action

|  | **Issue** | **Illustrative quote** | **Action** | **Rationale** |
| --- | --- | --- | --- | --- |
| 1 | Short of breath unclear to some patients according to professionals | And short of breath […] Because sometimes people consider shortness of breath [Nl; kortademigheid] and feeling stuffy [Nl: benauwdheid] to be very different things. I think it also depends a bit on the region, what terminology people give to it. […] No, because I even notice that sometimes I ask people: are you short of breath? No, but stuffy. So I thought that was striking [Pr]  You could also start there with whether there are problems with the lungs. They sometimes find the question of shortness of breath very difficult [Pr] | None | No better suitable alternative available; likely to be understood by most patients as this seems a regional problem |
| 2 | Skin discoloration can have different meanings | Skin discoloration, because yeah, that's a bit vague I think, skin discoloration. […] It can either become very white, but you can also get red skin [P] | None | Overarching question that captures both a change in redness and whiteness |
| 3 | Feeling weak not to be positioned with fatigue items | I couldn't deal with that question, because 'weak'... I immediately thought: 'That relates to muscles,' but that has nothing to do with this [P] | To be moved from energy to muscle items in the future | Participant associates feeling weak more with ‘muscles’ than with ‘energy’ |
| 4 | Lacking energy can be better phrased | I would interpret that as listless, so to speak. Yes, it is more clear to me than a lack of energy, yes [P] | Added | Lethargy item available which might suit some patients better |
| 5 | Vague meaning ‘urinating frequently’ | And I thought frequent urination was a bit of a vague thing, because what is ‘often’? I think you should add: 'more than so many times' [Pr] | None | No better suitable alternative available. Comprehension not affected |
| 6 | Constipation difficult phrasing | Just like constipation. That's way too difficult. Did you have difficulty passing stool? [P] | None | Both the difficult and more simple phrasing present in Dutch item, thus comprehension not affected |
| 7 | Problems (with coughing) considered unclear | Well, then I would rather ask 'have you been coughing, have you coughed excessively, other than with a cold?' […] Yes, have you had problems with coughing, then I would think […] what kind of problem could I have? That I can't cough it out? Do you understand? [P] | Alternative item chosen | Better suitable alternative (without problems) available |
| 8 | Tolerating (heat or cold) considered vague | How do you get to [this] question? With ‘tolerating’? I understand that you can't handle the cold or heat very well... but what do you do if you have problems with tolerating it? That's just: ‘I can't stand the cold or the heat’, right? [P] | None | No better suitable alternative available; comprehension not affected |
| 9 | Double negative phrasing considered complicated | It says: ‘are you less interested’? And then: ‘not at all’. Then it means you are not less interested at all. Or are you saying that I am not interested in sex at all? That's a bit unclear. Do you understand what I mean? It's actually a double negative then. That makes it difficult […] because it assumes that you were at least interested in sex [P] | None | The ‘double negative’ ensures that answering options all point in the same direction |
| 10 | Skin rash unclear meaning | What exactly do you mean by rash? […] I wonder what exactly is that? Are those bumps, or something […]? [P] | None | No alternative available. Retained as rash (Nl: uitslag) is a very common word |
| 11 | (Difficulty remembering) things considered vague | I didn't like that question very much. “Have you had trouble remembering things?” […] 'things' is also a somewhat difficult word […] 'Are you forgetful?' perhaps that is a better word [P] | None | Alternative available (forgetfulness), however, trouble remembering is more mild compared to forgetfulness so retained |
| 12 | Hot flushes Flemish phrase incomprehensible | And then further along there came 'vapeurs', yes, that is really Flemish then [Pr] | None | No alternative available. The item is still understandable as the Flemish word is an addition between brackets to the Netherlands Dutch phrasing |

Abbreviations: Nl: Dutch; P: patient; Pr: professional

Table 12 New comprehensiveness issues raised in the development phase, with illustrative quotes, rationale and decisive action

|  | **Issue** | **Illustrative quote** | **Action** | **Rationale** |
| --- | --- | --- | --- | --- |
| 1 | Dry vagina | Then the last question of “do you have difficulty getting/maintaining an erection?”. then I think yes, I can imagine when people have sex, uh, that women can also suffer from, for example, a dry vagina, that that might be annoying [P] | Added | Suitable item available |
| 2 | Leg pain during exercise | And oh yes, there is one more that I missed, uhm, at least I thought I hadn't seen it, pain in my legs after walking for a while. So claudication complaints [P] | Added | Suitable item available |
| 3 | Palpitations | I don't see any palpitations here yet, for example. And that doesn't hurt, but it is a side effect. I didn't really come across that later on either. […] palpitations can feel alarming or be a side effect that makes the hematologist say: maybe we'll do an ECG next time you have it again? [P] | Added | Suitable item available |
| 4 | Muscle stiffness | Only later did I realise that there are side effects that I did not find in your questionnaire, such as stiff muscles, poorer wound healing (for me even all mosquito bites become infected), nosebleeds, menstruation may change [P] | Check yearly | No suitable item available |
| 5 | Slow wound healing | See muscle stiffness | Added | Suitable item available |
| 6 | Skin infections, esp. acne | I suffered very badly from acne. And I hear that more often. […] In terms of painful inflammation under the skin. Particularly under the scalp [P] | Check yearly | No suitable item available |
| 7 | Nose bleeds | See muscle stiffness | Added | Suitable item available |
| 8 | Change menstruation | See muscle stiffness | Check yearly | No suitable item available |
| 9 | Fatigue interference | […] because that lack of energy, or at least, you also have a score list for that. Sometimes a question is also asked in a different way, because can you still do all your activities as you were used to? [Pr] | None | Interference items not to be included due to many items, therefore interference due to pain also left out |
| 10 | Petechiae | You ask about bruises, you're not actually asked about, yes, you could say 'have you had a rash?' But I know my doctor at one point said if you get spot bleeds everywhere, then it's time to really sound the alarm [P] | Check yearly | No suitable item available |
| 11 | Change hair structure | By hair texture I mean I used to have straight hair and now I have curly hair [P] | Check yearly | Not available in Dutch |
| 12 | Change hair colour | We know that hair can also change colour [Pr]  My hair turned grey [P] | Check yearly | Not available in Dutch |
| 13 | Red eyes |  | Added | Suitable item available |
| 14 | Sensitivity sound | One of the things I really notice is that a lot of noise makes me very tired. So when I've been there on a school day, I'm so devastated afterwards because I've heard a lot of noise. So if I'm in a room having lunch with a whole group, I better decide to get out, otherwise I'll be completely devastated. I can't filter that anymore [P] | Check yearly | No suitable item available |
| 15 | Increased appetite | It is a lack of appetite or an increase in appetite, because it also causes an increase [Pr] | Check yearly | No suitable item available |
| 16 | Muscular pain after sex | What I personally experience is that due to more muscle tension, I have really bad muscles for two or three days, different than before. Do you understand what I mean? So I experience more discomfort afterwards [P] | Added | Suitable item available |
| 17 | Anxiety | And then I actually miss it anxiety. […] anxiety and depression […] those are actually often the emotions for which you can also have a diagnosis. This also allows you to do intervention, treatment, and referral, so it is practically relevant to make that distinction [Pr] | Added | Suitable item available |
| 18 | Interference muscle cramps |  | None | Interference items not to be included due to many items |
| 19 | Fine motor skills apraxia | I have polyneuropathy as a result of the medication and that is a very serious obstacle, I notice […] It becomes less easy to button buttons, it becomes more difficult to remove the meat from the packaging, and I also notice that I have become less handy with doing odd jobs, for example [P] | Added | Suitable item available |
| 20 | Teeth problems (tooth growth) | Yes, I'm missing a part about teeth […] but it seems to happen to more people that they have problems with their teeth or molars. Everyone has a cavity now and then and those kinds of things happen from time to time ... but I also notice that my teeth have become weaker […] for example this tooth comes up and the dentist says: “I really don't understand it at all.” [P] | Added | Suitable item available |
| 21 | Bleeding gums | Then I also had something like “well, maybe people have problems with their teeth” […] perhaps also dental problems […] toothache or maybe it's uh, gum bleeding, uhm, sensitive gums [P] | Added | Suitable item for bleeding gums available |

Abbreviations: P: patient; Pr: professional

# Detailed results quantitative validation

**Comprehensiveness**: At T1 and T2, 15 new issues were raised: for eight issues no suitable item was available (in Dutch), for six issues a suitable item was available and added; for one issue a better alternative was available and this item was switched (Table 13). At T3 no missing issues were raised. At T4, one missing issue was raised (dry eyes). This item had been included in the potential filter item from EORTC, which had been eliminated at T4 for lack of validity as a filter question.

Table 13 New comprehensiveness issues raised in the development phase, with remark made, rationale and decisive action

|  | Remark | Action | Rationale |
| --- | --- | --- | --- |
| 1 | Raynaud’s phenomenon | Added | Suitable item available |
| 2 | Chronic bladder infection | Check yearly | No suitable item available |
| 3 | Easily susceptible to common infectious diseases | Added | Suitable item available |
| 4 | Easily susceptible to upper respiratory tract infection | Added | Suitable item available |
| 5 | Sour taste | Switched items | More suitable item available |
| 6 | Nerve pain | Check yearly | No suitable item available |
| 7 | Absence of menstruation | Check yearly | No suitable item available |
| 8 | Slow heartbeat | Check yearly | No suitable item available |
| 9 | Greasy hair | Check yearly | No suitable item available |
| 10 | Rumbling and grunting intestines | Check yearly | Not available in Dutch |
| 11 | Receding gums | Check yearly | No suitable item available |
| 12 | Back pain | Added | Suitable item available |
| 13 | Problems with nails | Added | Suitable item available |
| 14 | Coughing with mucus | Check yearly | No suitable item available |
| 15 | Sneezing | Added | Suitable item available |
| 16 | Dry eyes | Added | Suitable item available |

# Final set of filter questions

Table 14 Final set of filter questions, translated from Dutch, and status of validation*

| Category name | Filter question ^$^ | Validated | Invalid responses at T4 | Adapted filter question, to be validated | Filters for EORTC |
| --- | --- | --- | --- | --- | --- |
| Fatigue | … fatigue (6 questions: tired, need to rest, lacked energy, lethargic, weak, drowsy) | Yes | 0 | Na | IL281 |
| Head and brain | … head or brain (5 questions: headache, dizziness, remembering, concentrating, thinking clearly) | Yes | 2 (5%) | Na | IL255 |
| Skin | … skin (6 questions: bruises, discoloration, itch, rash, dry/flaky/cracked, sore/painful) | Yes | 2 (6.5%) | Na | IL282 |
| Wound healing | … wound healing (1 question: wound healing) | Yes | 0 | Na | IL283 |
| Hair and nails | … hair or nails (2 questions: hair loss, nail problems) | Yes | 0 | Na | IL284 |
| Eyes | … eyes (5 questions: watery, burning, light sensitive, blurry, red) | Yes^£^ | 1 (3.6%) | … eyes (6 questions: watery, dry, burning, light sensitive, blurry, red) | IL387^£^ |
| Oedema | … oedema (2 questions: swelling eyes/face, swelling body parts) | Yes | 0 | Na | IL286 |
| Hearing | … hearing (1 question: hearing problems) | Yes | 0 | Na | IL258 |
| Nose bleeds and nose problems | … nose bleeds or nose problems (2 questions: nose bleeds, nose problems) | No | 14 (20.6%) | … nose bleeds or nose problems (2 questions: nose bleeds, nose problems (smell, runny/blocked, sneezing, sore, dry)) | IL259 |
| Mouth and teeth | … mouth or teeth (4 questions: dry mouth, pain/sore mouth, teeth problems, bleeding gums) | Yes | 1 (2.4%) | Na | IL260 |
| Food and taste | … food or taste (2 questions: taste, appetite) | Yes | 3 (5%) | Na | IL261 |
| Mood | … mood (6 questions: depressed, irritable, tense, worried, afraid, difficulty sleeping) | Yes | 3 (7.3%) | Na | IL287 |
| Heart | … heart (2 questions: palpitations, pain in chest) | Yes | 0 | Na | IL263 |
| Breathing | … breathing (2 questions: short of breath, coughing) | Yes | 0 | Na | IL264 |
| Stomach and intestines | … stomach or intestines (8 questions: bloated, indigestion, nausea, vomiting, diarrhoea, constipation, abdominal pain/cramps, flatulence) | Yes | 1 (3.7%) | Na | IL265 |
| Urination | … urination (1 question: frequent urination) | Yes | 1 (2.6%) | Na | IL266 |
| Muscles and joints | … muscles or joints (3 questions: muscle cramps, muscle/joint ache/pain, muscle weakness) | Yes | 0 | Na | IL288 |
| Fingers and toes | … fingers or toes (3 questions: pale/cold, tingling/numb, dexterity) | Yes | 1 (2.9%) | Na | IL289 |
| Pain | … pain (3 questions: pain walking, back pain, pain in general) | Yes | 1 (3.2%) | Na | IL290 |
| Fever and sweating | … fever or sweating (2 questions: fever/chills, excessive sweating) | Yes | 3 (5.1%) | Na | IL291 |
| Warmth and cold | … warmth or cold (2 questions: tolerating warmth/cold, hot flushes) | Yes | 3 (7.5%) | Na | IL292 |
| Infection | … infections (2 questions: airway infections, other infections) | Yes | 1 (1.6%) | Na | IL293 |
| Nipples and breasts | … nipples or breasts (1 question: sore/enlarged nipples/breasts) | Yes | 0 | Na | IL294 |
| Sexuality | … sexuality (5 questions: interest, pleasure, pain, problems erection, dry vagina) | No | 6 (12.8%) | … sexuality (5 questions: interest in sex, pleasure from sex, pain, problems erection, dry vagina) | IL295 |

*Validation in Dutch only. Validity defined as: <10% of patients, whom indicated not to have symptoms in a certain category, scored ‘quite a bit’ or ‘very much’ on any of the category items. ^$^All questions started: ‘During the past week: did you have any signs or symptoms of:’. For the Sexuality category this was: ‘During the past 4 weeks: did you have any signs or symptoms of:’. Answering options were: ‘Yes’ and ‘No, I have no symptoms or complaints on this topic’. ^£^ In the final version (EORTC IL387) an item for dry eyes was added. Abbreviations: na: not applicable

# Reliability and measurement error

Table 15 Reliability (weighted kappa) and measurement error (% positive agreement) of 70 items tested in both T1 and T2, in patients on the same TKI and same dosage

| Item | Weighted kappa | | | % positive agreement | | |
| --- | --- | --- | --- | --- | --- | --- |
|  | Weighted kappa | Lower 95% confidence interval | Upper 95% confidence interval | % positive agreement | Lower 95% confidence interval | Upper 95% confidence interval |
| Tired | 0.69 | 0.55 | 0.82 | 64.1 | 53.0 | 73.9 |
| Need to rest | 0.62 | 0.47 | 0.78 | 57.1 | 46.0 | 67.6 |
| Lacked energy | 0.69 | 0.58 | 0.80 | 53.8 | 42.9 | 64.5 |
| Lethargic | 0.57 | 0.42 | 0.73 | 54.5 | 43.5 | 65.2 |
| Weak | 0.50 | 0.31 | 0.69 | 54.7 | 43.4 | 65.4 |
| Drowsy | 0.54 | 0.40 | 0.67 | 48.0 | 37.1 | 59.1 |
| Headaches | 0.51 | 0.29 | 0.74 | 70.4 | 59.0 | 79.8 |
| Dizzy | 0.69 | 0.55 | 0.82 | 80.3 | 69.6 | 87.9 |
| Difficulty remembering | 0.48 | 0.28 | 0.68 | 58.6 | 46.9 | 69.4 |
| Difficulty concentrating | 0.76 | 0.65 | 0.88 | 66.7 | 54.9 | 76.6 |
| Trouble thinking clearly | 0.56 | 0.39 | 0.72 | 63.8 | 52.0 | 74.1 |
| Bruised easily | 0.66 | 0.47 | 0.86 | 72.5 | 61.0 | 81.6 |
| Skin colour change | 0.51 | 0.25 | 0.77 | 76.9 | 65.4 | 85.5 |
| Itchy skin | 0.60 | 0.43 | 0.76 | 59.4 | 47.1 | 70.5 |
| Skin rash | 0.64 | 0.42 | 0.85 | 73.4 | 61.5 | 82.7 |
| Dry/flaking/cracked skin | 0.47 | 0.29 | 0.64 | 45.3 | 33.7 | 57.4 |
| Sore/painful skin | 0.42 | 0.15 | 0.69 | 68.8 | 56.6 | 78.8 |
| Wounds healing problems | 0.45 | 0.11 | 0.78 | 86.4 | 76.1 | 92.7 |
| Hair loss | 0.72 | 0.58 | 0.86 | 76.9 | 65.4 | 85.5 |
| Watery eyes | 0.64 | 0.45 | 0.82 | 61.5 | 49.4 | 72.4 |
| Burning eyes | 0.41 | 0.16 | 0.66 | 53.1 | 41.1 | 64.8 |
| Discomfort bright light | 0.74 | 0.62 | 0.87 | 64.1 | 51.8 | 74.7 |
| Blurred vision | 0.47 | 0.23 | 0.71 | 51.6 | 39.6 | 63.4 |
| Red eyes | 0.82 | 0.69 | 0.95 | 88.7 | 78.5 | 94.4 |
| Swelling face/eyes | 0.76 | 0.65 | 0.86 | 65.2 | 53.1 | 75.5 |
| Swelling body parts | 0.72 | 0.56 | 0.88 | 75.0 | 62.8 | 84.2 |
| Hearing problems | 0.76 | 0.60 | 0.92 | 85.7 | 75.0 | 92.3 |
| Nose bleeds | 0.51 | 0.10 | 0.91 | 85.7 | 75.0 | 92.3 |
| Dry mouth | 0.80 | 0.69 | 0.92 | 68.8 | 56.6 | 78.8 |
| Pain/soreness mouth | 0.64 | 0.47 | 0.81 | 71.9 | 59.9 | 81.4 |
| Teeth problems | 0.69 | 0.53 | 0.85 | 75.0 | 63.2 | 84.0 |
| Bleeding gums | 0.76 | 0.56 | 0.96 | 84.1 | 73.2 | 91.1 |
| Taste change | 0.49 | 0.27 | 0.71 | 68.8 | 56.6 | 78.8 |
| Appetite loss | 0.79 | 0.71 | 0.87 | 73.0 | 61.0 | 82.4 |
| Depressed | 0.66 | 0.51 | 0.80 | 67.2 | 55.0 | 77.4 |
| Irritable | 0.51 | 0.31 | 0.71 | 58.7 | 46.4 | 70.0 |
| Tense | 0.72 | 0.57 | 0.86 | 65.6 | 53.4 | 76.1 |
| Worried | 0.67 | 0.50 | 0.84 | 68.8 | 56.6 | 78.8 |
| Afraid | 0.71 | 0.54 | 0.88 | 77.8 | 66.1 | 86.3 |
| Difficulty sleeping | 0.67 | 0.50 | 0.83 | 58.6 | 46.9 | 69.4 |
| Palpitations | 0.72 | 0.61 | 0.82 | 64.1 | 51.8 | 74.7 |
| Pain in chest | 0.61 | 0.35 | 0.88 | 81.0 | 69.6 | 88.8 |
| Short of breath | 0.69 | 0.55 | 0.83 | 65.1 | 52.8 | 75.7 |
| Coughed | 0.51 | 0.30 | 0.72 | 69.4 | 57.0 | 79.4 |
| Feeling bloated | 0.59 | 0.44 | 0.75 | 50.8 | 38.8 | 62.7 |
| Indigestion | 0.74 | 0.62 | 0.87 | 63.5 | 51.1 | 74.3 |
| Nausea | 0.78 | 0.65 | 0.91 | 71.0 | 58.7 | 80.8 |
| Vomited | 0.40 | -0.09 | 0.88 | 85.7 | 75.0 | 92.3 |
| Diarrhoea | 0.78 | 0.65 | 0.90 | 73.0 | 61.0 | 82.4 |
| Obstipation | 0.73 | 0.55 | 0.90 | 73.0 | 61.0 | 82.4 |
| Abdominal pain/cramps | 0.74 | 0.59 | 0.88 | 67.7 | 55.4 | 78.0 |
| Flatulence | 0.81 | 0.71 | 0.90 | 65.6 | 53.0 | 76.3 |
| Frequent urination | 0.73 | 0.61 | 0.85 | 57.4 | 44.9 | 69.0 |
| Muscle cramps | 0.64 | 0.47 | 0.82 | 50.8 | 38.6 | 62.9 |
| Aches/pains muscles/joints | 0.74 | 0.64 | 0.83 | 50.8 | 38.6 | 62.9 |
| Muscle weakness | 0.48 | 0.26 | 0.70 | 51.7 | 39.3 | 63.8 |
| Tingling/numbness hands/feet | 0.75 | 0.63 | 0.86 | 63.9 | 51.4 | 74.8 |
| Problems finger dexterity | 0.60 | 0.43 | 0.77 | 72.9 | 60.4 | 82.6 |
| Trouble walking pain | 0.70 | 0.53 | 0.87 | 75.4 | 63.3 | 84.5 |
| Pain | 0.80 | 0.68 | 0.92 | 78.3 | 66.4 | 86.9 |
| Fever/chills | 0.62 | 0.39 | 0.85 | 83.3 | 72.0 | 90.7 |
| Sweating excessively | 0.65 | 0.46 | 0.84 | 65.0 | 52.4 | 75.8 |
| Problems tolerating heat/cold | 0.68 | 0.48 | 0.89 | 66.7 | 54.1 | 77.3 |
| Hot flushes | 0.66 | 0.39 | 0.94 | 76.3 | 64.0 | 85.3 |
| Sore/enlarged nipples/breasts | 0.54 | 0.14 | 0.93 | 93.3 | 84.1 | 97.4 |
| Less interest in sex | 0.66 | 0.49 | 0.84 | 67.2 | 54.4 | 77.9 |
| Less sexual enjoyment | 0.42 | 0.19 | 0.64 | 53.4 | 40.8 | 65.7 |
| Pain during/after sex | 0.82 | 0.66 | 0.98 | 86.2 | 75.1 | 92.8 |
| Difficulty getting/maintaining erection | 0.58 | 0.30 | 0.86 | 66.7 | 48.8 | 80.8 |
| Dry vagina | 0.89 | 0.80 | 0.98 | 78.6 | 60.5 | 89.8 |

# Scale construction

Table 16 Constructed scales with included items and Cronbach’s alpha

| **(Sub)scale** | **Included items** | **Number of included items** | **Cronbach’s alpha** |
| --- | --- | --- | --- |
| Fatigue | All fatigue category items | 6 | 0.93 (0.91 – 0.94) |
| Mood problems | All mood category items | 6 | 0.86 (0.83 – 0.88) |
| Muscle/joint pain/cramps | All category items plus items on pain walking and pain in general | 5 | 0.82 (0.79 – 0.85) |
| Skin problems | All skin category items except ‘bruising’ | 5 | 0.77 (0.72 – 0.81) |
| Eye problems | All eye category items | 5 | 0.73 (0.67 – 0.78) |
| Cognitive problems | All category items except headache and dizziness | 3 | 0.88 (0.85 – 0.90) |
| Sexuality problems | All category items except gender-specific items | 3 | 0.80 (0.75 – 0.83) |

# Responsiveness

50 Symptoms/scales were available both in T3-T4 and as anchor item/scales. Five symptoms/scales had only constant data in one of the pairs (either change from T3 to T4 or the anchor item) so correlation could not be evaluated. 25 out of 45 remaining symptoms/scales (56.6%) had an acceptable (≥0.30) association between the anchor and the change score. For these 25 symptoms/scales, groups with stable symptoms, worse, stable and better symptoms were compared (Table 17). The median change across these 25 symptom/scales in improved groups was 29.6%, in stable groups 7.7%, and -18.5% in worsened groups. In 14/25 symptoms/scales the between-group difference was significant. In 21/25 symptoms/scales all mean changes differed in the expected directions, while in four symptoms/scales the improved group improved less than the stable group, though differences were very small (<3%) or only one data point was available for the improved group.

Table 17 Mean change in mean symptom severity scores for patients whom reported improved, stable or worsened symptoms between T3 and T4, for symptoms with an acceptable (>0.30) Spearman’s rank correlation between item/scale score changes and anchor scores

|  | **Mean change symptom severity (95%CI)** | | |  |
| --- | --- | --- | --- | --- |
| **Symptom/scale** | **Improved** | **Stable** | **Worsened** | **p value*** |
| Fatigue (scale) | 18.5 (2.5 to 34.5) | 8.5 (4.5 to 12.5) | 2.5 (-6.7 to 11.8) | 0.076 |
| Cognitive problems(scale) | 29.6 (13.7 to 45.5) | 6.8 (3.1 to 104) | -16.7 (NA) | 0.030 |
| Hair loss | 50.0 (-8.7 to 108.7) | -1.0 (-4.0 to 2.1) | -8.3 (-25.8 to 9.1) | 0.0029 |
| Headaches | 22.2 (-25.6 to 70.0) | 4.5 (-1.1 to 10.1) | -66.6 (NA) | 0.073 |
| Eye problems (scale) | 2.2 (-2.6 to 7.0) | 3.3 (0.5 to 6.1) | -5.2 (-12.4 to 2.0) | 0.17 |
| Swelling face/eyes | 16.7 (-20.8 to 54.1) | 2.6 (-1.7 to 7.0) | -20.0 (-47.5 to 7.6) | 0.095 |
| Hearing problems | 66.6 (NA) | 1.7 (-4.1 to 7.4) | -16.7 (-228.2 to 194.9) | 0.13 |
| Nose problems | NA | 22.2 (15.3 to 29.1) | 0 (0 to 0) | 0.27 |
| Appetite loss | NA | 27.1 (19.2 to 35.0) | -33.3 (NA) | 0.08 |
| Mood problems (scale) | 38.9 (32.0 to 45.8) | 3.7 (-1.8 to 9.2) | -0.7 (-6.5 to 5.0) | <0.01 |
| Pain in chest | 0 (NA) | 11.1 (7.3 to 14.9) | -16.7 (-36.8 to 3.5) | <0.05 |
| Short of breath | 33.3 (-110.1 to 176.8) | 10.7 (5.6 to 15.7) | -9.5 (-23.1 to 4.1) | 0.082 |
| Feeling bloated | NA | 12.3 (7.6 to 17.0) | -19.1 (-37.8 to -0.3) | 0.0038 |
| Indigestion | NA | 4.8 (-0.6 to 10.2) | -29.7 (-57.2 to 3.8) | 0.0051 |
| Vomited | NA | 2.3 (-27.5 to 65.6) | -33.3 (-33.3 to -33.3) | <0.0001 |
| Diarrhoea | 19.0 (-27.5 to 65.6) | 9.3 (5.2 to 13.3) | -33.3 (-33.3 to -33.3) | 0.0065 |
| Abdominal pain/cramps | 33.4 (32.7 to 34.0) | 13.7 (7.3 to 20.1) | -18.5 (-33.9 to -3.1) | 0.0016 |
| Flatulence | 33.4 (NA) | 5.4 (-0.5 to 11.3) | -14.3 (-25.6 to -3.0) | 0.023 |
| Muscle/joints cramps/pain | 5.0 (-3.5 to 13.5) | 8.0 (3.4 to 12.5) | -1.1 (-7.5 to 5.3) | 0.18 |
| Pale/cold fingers/toes | NA | -0.0 (-4.7 to 4.7) | -47.6 (-65.2 to -30.0) | <0.0001 |
| Tingling/numbness hands/feet | NA | 7.4 (0.6 to 14.2) | -18.5 (-29.1 to -7.9) | 0.058 |
| Back pain | 0 (NA) | 11.8 (7.2 to 16.4) | -18.5 (-42.7 to 5.6) | 0.0093 |
| Airway infections | 22.2 (-88.2 to 132.6) | 10.6 (4.4 to 16.7) | -50.0 (-87.4 to -12.6) | 0.028 |
| Other infections | 50 (-125.7 to 225.7) | 11.1 (2.9 to 19.3) | -66.7 (-183.8 to 50.5) | 0.050 |
| Sore/enlarged nipples/breasts | 100 (NA) | 4.6 (1.3 to 7.9) | NA | 0.023 |
| **Median** | **29**.**6** | **7.7** | **-18.5** |  |

* Kruskal-Wallis rank sum test. Shaded areas indicate a significant (p<0.05) differences between groups. NA: not available

# Prevalence of symptom score categories

Table 18 Prevalence (% with 95%confidence intervals) of symptom scores for seven scales plus 76 single items, in 12 patients on asciminib on T1

| Item/scale | Asciminib | | | | | |
| --- | --- | --- | --- | --- | --- | --- |
|  | Not at all | A little | Quite a bit | A lot | Any severity | Moderate-severe $ |
| Fatigue (scale) | 8.3 (0.2-38.5) | Na | Na | Na | 91.7 (61.5-99.8) | Na |
| Tired | 16.7 (2.1-48.4) | 33.3 (9.9-65.1) | 25.0 (5.5-57.2) | 25.0 (5.5-57.2) | 83.3 (51.6-97.9) | 50.0 (21.1-78.9) |
| Needed rest | 16.7 (2.1-48.4) | 41.7 (15.2-72.3) | 41.7 (15.2-72.3) | 0.0 (0.0-26.5) | 83.3 (51.6-97.9) | 41.7 (15.2-72.3) |
| Lacked energy | 9.1 (0.2-41.3) | 45.5 (16.7-76.6) | 36.4 (10.9-69.2) | 9.1 (0.2-41.3) | 90.9 (58.7-99.8) | 45.5 (16.7-76.6) |
| Felt lethargic | 30.0 (6.7-65.2) | 40.0 (12.2-73.8) | 20.0 (2.5-55.6) | 10.0 (0.3-44.5) | 70.0 (34.8-93.3) | 30.0 (6.7-65.2) |
| Felt weak | 10.0 (0.3-44.5) | 70.0 (34.8-93.3) | 10.0 (0.3-44.5) | 10.0 (0.3-44.5) | 90.0 (55.5-99.7) | 20.0 (2.5-55.6) |
| Sleepy | 20.0 (2.5-55.6) | 70.0 (34.8-93.3) | 0.0 (0.0-30.8) | 10.0 (0.3-44.5) | 80.0 (44.4-97.5) | 10.0 (0.3-44.5) |
| Headaches | 30.0 (6.7-65.2) | 60.0 (26.2-87.8) | 10.0 (0.3-44.5) | 0.0 (0.0-30.8) | 70.0 (34.8-93.3) | 10.0 (0.3-44.5) |
| Dizziness | 44.4 (13.7-78.8) | 22.2 (2.8-60.0) | 22.2 (2.8-60.0) | 11.1 (0.3-48.2) | 55.6 (21.2-86.3) | 33.3 (7.5-70.1) |
| Cognitive problems (scale) | 11.1 (0.3-48.2) | Na | Na | Na | 88.9 (51.8-99.7) | Na |
| Difficulty remembering | 33.3 (7.5-70.1) | 44.4 (13.7-78.8) | 22.2 (2.8-60.0) | 0.0 (0.0-33.6) | 66.7 (29.9-92.5) | 22.2 (2.8-60.0) |
| Difficulty concentrating | 22.2 (2.8-60.0) | 66.7 (29.9-92.5) | 11.1 (0.3-48.2) | 0.0 (0.0-33.6) | 77.8 (40.0-97.2) | 11.1 (0.3-48.2) |
| Trouble thinking clearly | 55.6 (21.2-86.3) | 33.3 (7.5-70.1) | 11.1 (0.3-48.2) | 0.0 (0.0-33.6) | 44.4 (13.7-78.8) | 11.1 (0.3-48.2) |
| Bruised easily | 55.6 (21.2-86.3) | 11.1 (0.3-48.2) | 11.1 (0.3-48.2) | 22.2 (2.8-60.0) | 44.4 (13.7-78.8) | 33.3 (7.5-70.1) |
| Skin problems (scale) | 11.1 (0.3-48.2) | Na | Na | Na | 91.7 (61.5-99.8) | Na |
| Skin colour change | 77.8 (40.0-97.2) | 11.1 (0.3-48.2) | 0.0 (0.0-33.6) | 11.1 (0.3-48.2) | 22.2 (2.8-60.0) | 11.1 (0.3-48.2) |
| Skin itched | 22.2 (2.8-60.0) | 55.6 (21.2-86.3) | 11.1 (0.3-48.2) | 11.1 (0.3-48.2) | 77.8 (40.0-97.2) | 22.2 (2.8-60.0) |
| Skin rash | 22.2 (2.8-60.0) | 66.7 (29.9-92.5) | 0.0 (0.0-33.6) | 11.1 (0.3-48.2) | 77.8 (40.0-97.2) | 11.1 (0.3-48.2) |
| Skin dry/flaky/cracked | 22.2 (2.8-60.0) | 44.4 (13.7-78.8) | 22.2 (2.8-60.0) | 11.1 (0.3-48.2) | 77.8 (40.0-97.2) | 33.3 (7.5-70.1) |
| Skin sore/painful | 55.6 (21.2-86.3) | 33.3 (7.5-70.1) | 11.1 (0.3-48.2) | 0.0 (0.0-33.6) | 44.4 (13.7-78.8) | 11.1 (0.3-48.2) |
| Wounds healing problems | 77.8 (40.0-97.2) | 22.2 (2.8-60.0) | 0.0 (0.0-33.6) | 0.0 (0.0-33.6) | 22.2 (2.8-60.0) | 0.0 (0.0-33.6) |
| Hair loss | 55.6 (21.2-86.3) | 33.3 (7.5-70.1) | 0.0 (0.0-33.6) | 11.1 (0.3-48.2) | 44.4 (13.7-78.8) | 11.1 (0.3-48.2) |
| Nail problems | 67.9 (56.4-78.1) | 20.5 (12.2-31.2) | 9.0 (3.7-17.6) | 2.6 (0.3-9.0) | 32.1 (21.9-43.6) | 11.5 (5.4-20.8) |
| Eye problems (scale) | 22.2 (2.8-60.0) | Na | Na | Na | 77.8 (40.0-97.2) | Na |
| Watery eyes | 33.3 (7.5-70.1) | 22.2 (2.8-60.0) | 44.4 (13.7-78.8) | 0.0 (0.0-33.6) | 66.7 (29.9-92.5) | 44.4 (13.7-78.8) |
| Burning eyes | 33.3 (7.5-70.1) | 22.2 (2.8-60.0) | 44.4 (13.7-78.8) | 0.0 (0.0-33.6) | 66.7 (29.9-92.5) | 44.4 (13.7-78.8) |
| Light sensitive eyes | 55.6 (21.2-86.3) | 0.0 (0.0-33.6) | 33.3 (7.5-70.1) | 11.1 (0.3-48.2) | 44.4 (13.7-78.8) | 44.4 (13.7-78.8) |
| Blurry eyes | 33.3 (7.5-70.1) | 55.6 (21.2-86.3) | 11.1 (0.3-48.2) | 0.0 (0.0-33.6) | 66.7 (29.9-92.5) | 11.1 (0.3-48.2) |
| Red eyes | 75.0 (34.9-96.8) | 25.0 (3.2-65.1) | 0.0 (0.0-36.9) | 0.0 (0.0-36.9) | 25.0 (3.2-65.1) | 0.0 (0.0-36.9) |
| Swelling face/eyes | 77.8 (40.0-97.2) | 11.1 (0.3-48.2) | 0.0 (0.0-33.6) | 11.1 (0.3-48.2) | 22.2 (2.8-60.0) | 11.1 (0.3-48.2) |
| Swelling body parts | 87.5 (47.3-99.7) | 12.5 (0.3-52.7) | 0.0 (0.0-36.9) | 0.0 (0.0-36.9) | 12.5 (0.3-52.7) | 0.0 (0.0-36.9) |
| Hearing problems | 75.0 (34.9-96.8) | 0.0 (0.0-36.9) | 25.0 (3.2-65.1) | 0.0 (0.0-36.9) | 25.0 (3.2-65.1) | 25.0 (3.2-65.1) |
| Nose bleeds | 50.0 (15.7-84.3) | 37.5 (8.5-75.5) | 0.0 (0.0-36.9) | 12.5 (0.3-52.7) | 50.0 (15.7-84.3) | 12.5 (0.3-52.7) |
| Nose problems | 40.0 (5.3-85.3) | 0.0 (0.0-52.2) | 60.0 (14.7-94.7) | 0.0 (0.0-52.2) | 60.0 (14.7-94.7) | 60.0 (14.7-94.7) |
| Dry mouth | 25.0 (3.2-65.1) | 50.0 (15.7-84.3) | 12.5 (0.3-52.7) | 12.5 (0.3-52.7) | 75.0 (34.9-96.8) | 25.0 (3.2-65.1) |
| Pain/soreness mouth | 50.0 (15.7-84.3) | 25.0 (3.2-65.1) | 25.0 (3.2-65.1) | 0.0 (0.0-36.9) | 50.0 (15.7-84.3) | 25.0 (3.2-65.1) |
| Teeth problems | 62.5 (24.5-91.5) | 25.0 (3.2-65.1) | 12.5 (0.3-52.7) | 0.0 (0.0-36.9) | 37.5 (8.5-75.5) | 12.5 (0.3-52.7) |
| Bleeding gums | 87.5 (47.3-99.7) | 0.0 (0.0-36.9) | 12.5 (0.3-52.7) | 0.0 (0.0-36.9) | 12.5 (0.3-52.7) | 12.5 (0.3-52.7) |
| Taste change | 37.5 (8.5-75.5) | 50.0 (15.7-84.3) | 12.5 (0.3-52.7) | 0.0 (0.0-36.9) | 62.5 (24.5-91.5) | 12.5 (0.3-52.7) |
| Appetite loss | 37.5 (8.5-75.5) | 37.5 (8.5-75.5) | 12.5 (0.3-52.7) | 12.5 (0.3-52.7) | 62.5 (24.5-91.5) | 25.0 (3.2-65.1) |
| Mood problems (scale) | 10.0 (0.3-44.5) | Na | Na | Na | 90.0 (55.5-99.7) | Na |
| Depressed | 50.0 (15.7-84.3) | 25.0 (3.2-65.1) | 12.5 (0.3-52.7) | 12.5 (0.3-52.7) | 50.0 (15.7-84.3) | 25.0 (3.2-65.1) |
| Irritable | 25.0 (3.2-65.1) | 50.0 (15.7-84.3) | 12.5 (0.3-52.7) | 12.5 (0.3-52.7) | 75.0 (34.9-96.8) | 25.0 (3.2-65.1) |
| Tense | 25.0 (3.2-65.1) | 37.5 (8.5-75.5) | 25.0 (3.2-65.1) | 12.5 (0.3-52.7) | 75.0 (34.9-96.8) | 37.5 (8.5-75.5) |
| Worried | 25.0 (3.2-65.1) | 25.0 (3.2-65.1) | 37.5 (8.5-75.5) | 12.5 (0.3-52.7) | 75.0 (34.9-96.8) | 50.0 (15.7-84.3) |
| Afraid | 50.0 (15.7-84.3) | 25.0 (3.2-65.1) | 12.5 (0.3-52.7) | 12.5 (0.3-52.7) | 50.0 (15.7-84.3) | 25.0 (3.2-65.1) |
| Insomnia | 30.0 (6.7-65.2) | 30.0 (6.7-65.2) | 20.0 (2.5-55.6) | 20.0 (2.5-55.6) | 70.0 (34.8-93.3) | 40.0 (12.2-73.8) |
| Palpitations | 37.5 (8.5-75.5) | 37.5 (8.5-75.5) | 12.5 (0.3-52.7) | 12.5 (0.3-52.7) | 62.5 (24.5-91.5) | 25.0 (3.2-65.1) |
| Pain in chest | 50.0 (15.7-84.3) | 37.5 (8.5-75.5) | 0.0 (0.0-36.9) | 12.5 (0.3-52.7) | 50.0 (15.7-84.3) | 12.5 (0.3-52.7) |
| Short of breath | 25.0 (3.2-65.1) | 25.0 (3.2-65.1) | 37.5 (8.5-75.5) | 12.5 (0.3-52.7) | 75.0 (34.9-96.8) | 50.0 (15.7-84.3) |
| Coughed | 25.0 (3.2-65.1) | 25.0 (3.2-65.1) | 37.5 (8.5-75.5) | 12.5 (0.3-52.7) | 75.0 (34.9-96.8) | 50.0 (15.7-84.3) |
| Feeling bloated | 50.0 (15.7-84.3) | 0.0 (0.0-36.9) | 12.5 (0.3-52.7) | 37.5 (8.5-75.5) | 50.0 (15.7-84.3) | 50.0 (15.7-84.3) |
| Indigestion | 37.5 (8.5-75.5) | 37.5 (8.5-75.5) | 12.5 (0.3-52.7) | 12.5 (0.3-52.7) | 62.5 (24.5-91.5) | 25.0 (3.2-65.1) |
| Nausea | 50.0 (15.7-84.3) | 12.5 (0.3-52.7) | 37.5 (8.5-75.5) | 0.0 (0.0-36.9) | 50.0 (15.7-84.3) | 37.5 (8.5-75.5) |
| Diarrhoea | 75.0 (34.9-96.8) | 0.0 (0.0-36.9) | 0.0 (0.0-36.9) | 25.0 (3.2-65.1) | 25.0 (3.2-65.1) | 25.0 (3.2-65.1) |
| Abdominal pain/cramps | 37.5 (8.5-75.5) | 12.5 (0.3-52.7) | 37.5 (8.5-75.5) | 12.5 (0.3-52.7) | 62.5 (24.5-91.5) | 50.0 (15.7-84.3) |
| Vomited | 87.5 (47.3-99.7) | 0.0 (0.0-36.9) | 12.5 (0.3-52.7) | 0.0 (0.0-36.9) | 12.5 (0.3-52.7) | 12.5 (0.3-52.7) |
| Obstipation | 37.5 (8.5-75.5) | 50.0 (15.7-84.3) | 0.0 (0.0-36.9) | 12.5 (0.3-52.7) | 62.5 (24.5-91.5) | 12.5 (0.3-52.7) |
| Flatulence | 37.5 (8.5-75.5) | 50.0 (15.7-84.3) | 0.0 (0.0-36.9) | 12.5 (0.3-52.7) | 62.5 (24.5-91.5) | 12.5 (0.3-52.7) |
| Muscle/joint cramps/pain (scale) | 0.0 (0.0-36.9) | Na | Na | Na | 100.0 (63.1-100.0) | Na |
| Frequent urination | 50.0 (15.7-84.3) | 12.5 (0.3-52.7) | 12.5 (0.3-52.7) | 25.0 (3.2-65.1) | 50.0 (15.7-84.3) | 37.5 (8.5-75.5) |
| Muscle cramps | 37.5 (8.5-75.5) | 50.0 (15.7-84.3) | 0.0 (0.0-36.9) | 12.5 (0.3-52.7) | 62.5 (24.5-91.5) | 12.5 (0.3-52.7) |
| Aches/pains muscles/joints | 12.5 (0.3-52.7) | 25.0 (3.2-65.1) | 50.0 (15.7-84.3) | 12.5 (0.3-52.7) | 87.5 (47.3-99.7) | 62.5 (24.5-91.5) |
| Muscle weakness | 42.9 (9.9-81.6) | 42.9 (9.9-81.6) | 14.3 (0.4-57.9) | 0.0 (0.0-41.0) | 57.1 (18.4-90.1) | 14.3 (0.4-57.9) |
| Pale/cold fingers/toes | 80.0 (28.4-99.5) | 0.0 (0.0-52.2) | 0.0 (0.0-52.2) | 20.0 (0.5-71.6) | 20.0 (0.5-71.6) | 20.0 (0.5-71.6) |
| Tingling/numbness hands/feet | 50.0 (15.7-84.3) | 37.5 (8.5-75.5) | 12.5 (0.3-52.7) | 0.0 (0.0-36.9) | 50.0 (15.7-84.3) | 12.5 (0.3-52.7) |
| Problems finger dexterity | 50.0 (15.7-84.3) | 37.5 (8.5-75.5) | 12.5 (0.3-52.7) | 0.0 (0.0-36.9) | 50.0 (15.7-84.3) | 12.5 (0.3-52.7) |
| Trouble walking pain | 37.5 (8.5-75.5) | 62.5 (24.5-91.5) | 0.0 (0.0-36.9) | 0.0 (0.0-36.9) | 62.5 (24.5-91.5) | 0.0 (0.0-36.9) |
| Back pain | 40.0 (5.3-85.3) | 40.0 (5.3-85.3) | 0.0 (0.0-52.2) | 20.0 (0.5-71.6) | 60.0 (14.7-94.7) | 20.0 (0.5-71.6) |
| Pain | 25.0 (3.2-65.1) | 12.5 (0.3-52.7) | 50.0 (15.7-84.3) | 12.5 (0.3-52.7) | 75.0 (34.9-96.8) | 62.5 (24.5-91.5) |
| Fever/chills | 50.0 (15.7-84.3) | 37.5 (8.5-75.5) | 0.0 (0.0-36.9) | 12.5 (0.3-52.7) | 50.0 (15.7-84.3) | 12.5 (0.3-52.7) |
| Sweating excessively | 62.5 (24.5-91.5) | 12.5 (0.3-52.7) | 12.5 (0.3-52.7) | 12.5 (0.3-52.7) | 37.5 (8.5-75.5) | 25.0 (3.2-65.1) |
| Problems tolerating heat/cold | 62.5 (24.5-91.5) | 25.0 (3.2-65.1) | 0.0 (0.0-36.9) | 12.5 (0.3-52.7) | 37.5 (8.5-75.5) | 12.5 (0.3-52.7) |
| Hot flushes | 62.5 (24.5-91.5) | 25.0 (3.2-65.1) | 0.0 (0.0-36.9) | 12.5 (0.3-52.7) | 37.5 (8.5-75.5) | 12.5 (0.3-52.7) |
| Airway infections | 80.0 (28.4-99.5) | 20.0 (0.5-71.6) | 0.0 (0.0-52.2) | 0.0 (0.0-52.2) | 20.0 (0.5-71.6) | 0.0 (0.0-52.2) |
| Other infections | 100.0 (47.8-100.0) | 0.0 (0.0-52.2) | 0.0 (0.0-52.2) | 0.0 (0.0-52.2) | 0.0 (0.0-52.2) | 0.0 (0.0-52.2) |
| Sore/enlarged nipples/breasts | 100.0 (63.1-100.0) | 0.0 (0.0-36.9) | 0.0 (0.0-36.9) | 0.0 (0.0-36.9) | 0.0 (0.0-36.9) | 0.0 (0.0-36.9) |
| Sexuality problems (scale) | 25.0 (3.2-65.1) | Na | Na | Na | 75.0 (34.9-96.8) | Na |
| Less interest sex | 25.0 (3.2-65.1) | 37.5 (8.5-75.5) | 37.5 (8.5-75.5) | 0.0 (0.0-36.9) | 75.0 (34.9-96.8) | 37.5 (8.5-75.5) |
| Less pleasure sex | 37.5 (8.5-75.5) | 37.5 (8.5-75.5) | 25.0 (3.2-65.1) | 0.0 (0.0-36.9) | 62.5 (24.5-91.5) | 25.0 (3.2-65.1) |
| Pain during/after sex | 75.0 (34.9-96.8) | 25.0 (3.2-65.1) | 0.0 (0.0-36.9) | 0.0 (0.0-36.9) | 25.0 (3.2-65.1) | 0.0 (0.0-36.9) |
| Difficult erection | 25.0 (0.6-80.6) | 50.0 (6.8-93.2) | 25.0 (0.6-80.6) | 0.0 (0.0-60.2) | 75.0 (19.4-99.4) | 25.0 (0.6-80.6) |
| Dry vagina | 75.0 (19.4-99.4) | 25.0 (0.6-80.6) | 0.0 (0.0-60.2) | 0.0 (0.0-60.2) | 25.0 (0.6-80.6) | 0.0 (0.0-60.2) |

* At T4 as not available at T1. $ Moderate-severe includes ‘Quite a bit’ and ‘A lot’. Abbreviations: Na: not applicable

Table 19 Prevalence (% with 95%confidence intervals) of symptom scores for seven scales plus 76 single items, in 14 patients on bosutinib on T1

| Item/scale | Bosutinib | | | | | |
| --- | --- | --- | --- | --- | --- | --- |
|  | Not at all | A little | Quite a bit | A lot | Any severity | Moderate-severe $ |
| Fatigue (scale) | 0.0 (0.0-23.2) | Na | Na | Na | 100.0 (76.8-100.0) | Na |
| Tired | 7.1 (0.2-33.9) | 50.0 (23.0-77.0) | 21.4 (4.7-50.8) | 21.4 (4.7-50.8) | 92.9 (66.1-99.8) | 42.9 (17.7-71.1) |
| Needed rest | 0.0 (0.0-23.2) | 71.4 (41.9-91.6) | 28.6 (8.4-58.1) | 0.0 (0.0-23.2) | 100.0 (76.8-100.0) | 28.6 (8.4-58.1) |
| Lacked energy | 7.1 (0.2-33.9) | 35.7 (12.8-64.9) | 42.9 (17.7-71.1) | 14.3 (1.8-42.8) | 92.9 (66.1-99.8) | 57.1 (28.9-82.3) |
| Felt lethargic | 21.4 (4.7-50.8) | 50.0 (23.0-77.0) | 28.6 (8.4-58.1) | 0.0 (0.0-23.2) | 78.6 (49.2-95.3) | 28.6 (8.4-58.1) |
| Felt weak | 46.2 (19.2-74.9) | 15.4 (1.9-45.4) | 30.8 (9.1-61.4) | 7.7 (0.2-36.0) | 53.8 (25.1-80.8) | 38.5 (13.9-68.4) |
| Sleepy | 23.1 (5.0-53.8) | 53.8 (25.1-80.8) | 15.4 (1.9-45.4) | 7.7 (0.2-36.0) | 76.9 (46.2-95.0) | 23.1 (5.0-53.8) |
| Headaches | 72.7 (39.0-94.0) | 18.2 (2.3-51.8) | 9.1 (0.2-41.3) | 0.0 (0.0-28.5) | 27.3 (6.0-61.0) | 9.1 (0.2-41.3) |
| Dizziness | 63.6 (30.8-89.1) | 36.4 (10.9-69.2) | 0.0 (0.0-28.5) | 0.0 (0.0-28.5) | 36.4 (10.9-69.2) | 0.0 (0.0-28.5) |
| Cognitive problems (scale) | 18.2 (2.3-51.8) | Na | Na | Na | 75.0 (34.9-96.8) | 81.8 (48.2-97.7) |
| Difficulty remembering | 27.3 (6.0-61.0) | 36.4 (10.9-69.2) | 27.3 (6.0-61.0) | 9.1 (0.2-41.3) | 72.7 (39.0-94.0) | 36.4 (10.9-69.2) |
| Difficulty concentrating | 36.4 (10.9-69.2) | 27.3 (6.0-61.0) | 18.2 (2.3-51.8) | 18.2 (2.3-51.8) | 63.6 (30.8-89.1) | 36.4 (10.9-69.2) |
| Trouble thinking clearly | 27.3 (6.0-61.0) | 36.4 (10.9-69.2) | 27.3 (6.0-61.0) | 9.1 (0.2-41.3) | 72.7 (39.0-94.0) | 36.4 (10.9-69.2) |
| Bruised easily | 45.5 (16.7-76.6) | 18.2 (2.3-51.8) | 27.3 (6.0-61.0) | 9.1 (0.2-41.3) | 54.5 (23.4-83.3) | 36.4 (10.9-69.2) |
| Skin problems (scale) | 18.2 (2.3-51.8) | Na | Na | Na | 75.0 (34.9-96.8) | 81.8 (48.2-97.7) |
| Skin colour change | 45.5 (16.7-76.6) | 36.4 (10.9-69.2) | 18.2 (2.3-51.8) | 0.0 (0.0-28.5) | 54.5 (23.4-83.3) | 18.2 (2.3-51.8) |
| Skin itched | 45.5 (16.7-76.6) | 27.3 (6.0-61.0) | 27.3 (6.0-61.0) | 0.0 (0.0-28.5) | 54.5 (23.4-83.3) | 27.3 (6.0-61.0) |
| Skin rash | 54.5 (23.4-83.3) | 36.4 (10.9-69.2) | 0.0 (0.0-28.5) | 9.1 (0.2-41.3) | 45.5 (16.7-76.6) | 9.1 (0.2-41.3) |
| Skin dry/flaky/cracked | 18.2 (2.3-51.8) | 27.3 (6.0-61.0) | 54.5 (23.4-83.3) | 0.0 (0.0-28.5) | 81.8 (48.2-97.7) | 54.5 (23.4-83.3) |
| Skin sore/painful | 54.5 (23.4-83.3) | 27.3 (6.0-61.0) | 18.2 (2.3-51.8) | 0.0 (0.0-28.5) | 45.5 (16.7-76.6) | 18.2 (2.3-51.8) |
| Wounds healing problems | 63.6 (30.8-89.1) | 27.3 (6.0-61.0) | 0.0 (0.0-28.5) | 9.1 (0.2-41.3) | 36.4 (10.9-69.2) | 9.1 (0.2-41.3) |
| Hair loss | 27.3 (6.0-61.0) | 54.5 (23.4-83.3) | 9.1 (0.2-41.3) | 9.1 (0.2-41.3) | 72.7 (39.0-94.0) | 18.2 (2.3-51.8) |
| Nail problems | 67.9 (56.4-78.1) | 20.5 (12.2-31.2) | 9.0 (3.7-17.6) | 2.6 (0.3-9.0) | 32.1 (21.9-43.6) | 11.5 (5.4-20.8) |
| Eye problems (scale) | 9.1 (0.2-41.3) | Na | Na | Na | 75.0 (34.9-96.8) | 90.9 (58.7-99.8) |
| Watery eyes | 45.5 (16.7-76.6) | 36.4 (10.9-69.2) | 18.2 (2.3-51.8) | 0.0 (0.0-28.5) | 54.5 (23.4-83.3) | 18.2 (2.3-51.8) |
| Burning eyes | 36.4 (10.9-69.2) | 45.5 (16.7-76.6) | 18.2 (2.3-51.8) | 0.0 (0.0-28.5) | 63.6 (30.8-89.1) | 18.2 (2.3-51.8) |
| Light sensitive eyes | 54.5 (23.4-83.3) | 27.3 (6.0-61.0) | 9.1 (0.2-41.3) | 9.1 (0.2-41.3) | 45.5 (16.7-76.6) | 18.2 (2.3-51.8) |
| Blurry eyes | 45.5 (16.7-76.6) | 45.5 (16.7-76.6) | 0.0 (0.0-28.5) | 9.1 (0.2-41.3) | 54.5 (23.4-83.3) | 9.1 (0.2-41.3) |
| Red eyes | 72.7 (39.0-94.0) | 27.3 (6.0-61.0) | 0.0 (0.0-28.5) | 0.0 (0.0-28.5) | 27.3 (6.0-61.0) | 0.0 (0.0-28.5) |
| Swelling face/eyes | 63.6 (30.8-89.1) | 27.3 (6.0-61.0) | 9.1 (0.2-41.3) | 0.0 (0.0-28.5) | 36.4 (10.9-69.2) | 9.1 (0.2-41.3) |
| Swelling body parts | 45.5 (16.7-76.6) | 18.2 (2.3-51.8) | 36.4 (10.9-69.2) | 0.0 (0.0-28.5) | 54.5 (23.4-83.3) | 36.4 (10.9-69.2) |
| Hearing problems | 60.0 (26.2-87.8) | 20.0 (2.5-55.6) | 10.0 (0.3-44.5) | 10.0 (0.3-44.5) | 40.0 (12.2-73.8) | 20.0 (2.5-55.6) |
| Nose bleeds | 81.8 (48.2-97.7) | 9.1 (0.2-41.3) | 9.1 (0.2-41.3) | 0.0 (0.0-28.5) | 18.2 (2.3-51.8) | 9.1 (0.2-41.3) |
| Nose problems | 40.0 (5.3-85.3) | 0.0 (0.0-52.2) | 40.0 (5.3-85.3) | 20.0 (0.5-71.6) | 60.0 (14.7-94.7) | 60.0 (14.7-94.7) |
| Dry mouth | 45.5 (16.7-76.6) | 36.4 (10.9-69.2) | 18.2 (2.3-51.8) | 0.0 (0.0-28.5) | 54.5 (23.4-83.3) | 18.2 (2.3-51.8) |
| Pain/soreness mouth | 72.7 (39.0-94.0) | 18.2 (2.3-51.8) | 0.0 (0.0-28.5) | 9.1 (0.2-41.3) | 27.3 (6.0-61.0) | 9.1 (0.2-41.3) |
| Teeth problems | 70.0 (34.8-93.3) | 0.0 (0.0-30.8) | 30.0 (6.7-65.2) | 0.0 (0.0-30.8) | 30.0 (6.7-65.2) | 30.0 (6.7-65.2) |
| Bleeding gums | 70.0 (34.8-93.3) | 20.0 (2.5-55.6) | 0.0 (0.0-30.8) | 10.0 (0.3-44.5) | 30.0 (6.7-65.2) | 10.0 (0.3-44.5) |
| Taste change | 63.6 (30.8-89.1) | 18.2 (2.3-51.8) | 9.1 (0.2-41.3) | 9.1 (0.2-41.3) | 36.4 (10.9-69.2) | 18.2 (2.3-51.8) |
| Appetite loss | 63.6 (30.8-89.1) | 27.3 (6.0-61.0) | 0.0 (0.0-28.5) | 9.1 (0.2-41.3) | 36.4 (10.9-69.2) | 9.1 (0.2-41.3) |
| Mood problems (scale) | 25.0 (5.5-57.2) | Na | Na | Na | 75.0 (34.9-96.8) | 75.0 (42.8-94.5) |
| Depressed | 45.5 (16.7-76.6) | 45.5 (16.7-76.6) | 9.1 (0.2-41.3) | 0.0 (0.0-28.5) | 54.5 (23.4-83.3) | 9.1 (0.2-41.3) |
| Irritable | 45.5 (16.7-76.6) | 36.4 (10.9-69.2) | 18.2 (2.3-51.8) | 0.0 (0.0-28.5) | 54.5 (23.4-83.3) | 18.2 (2.3-51.8) |
| Tense | 60.0 (26.2-87.8) | 10.0 (0.3-44.5) | 30.0 (6.7-65.2) | 0.0 (0.0-30.8) | 40.0 (12.2-73.8) | 30.0 (6.7-65.2) |
| Worried | 40.0 (12.2-73.8) | 30.0 (6.7-65.2) | 30.0 (6.7-65.2) | 0.0 (0.0-30.8) | 60.0 (26.2-87.8) | 30.0 (6.7-65.2) |
| Afraid | 22.2 (2.8-60.0) | 66.7 (29.9-92.5) | 11.1 (0.3-48.2) | 0.0 (0.0-33.6) | 77.8 (40.0-97.2) | 11.1 (0.3-48.2) |
| Insomnia | 58.3 (27.7-84.8) | 16.7 (2.1-48.4) | 25.0 (5.5-57.2) | 0.0 (0.0-26.5) | 41.7 (15.2-72.3) | 25.0 (5.5-57.2) |
| Palpitations | 54.5 (23.4-83.3) | 36.4 (10.9-69.2) | 0.0 (0.0-28.5) | 9.1 (0.2-41.3) | 45.5 (16.7-76.6) | 9.1 (0.2-41.3) |
| Pain in chest | 63.6 (30.8-89.1) | 27.3 (6.0-61.0) | 9.1 (0.2-41.3) | 0.0 (0.0-28.5) | 36.4 (10.9-69.2) | 9.1 (0.2-41.3) |
| Short of breath | 45.5 (16.7-76.6) | 27.3 (6.0-61.0) | 18.2 (2.3-51.8) | 9.1 (0.2-41.3) | 54.5 (23.4-83.3) | 27.3 (6.0-61.0) |
| Coughed | 45.5 (16.7-76.6) | 27.3 (6.0-61.0) | 27.3 (6.0-61.0) | 0.0 (0.0-28.5) | 54.5 (23.4-83.3) | 27.3 (6.0-61.0) |
| Feeling bloated | 36.4 (10.9-69.2) | 18.2 (2.3-51.8) | 27.3 (6.0-61.0) | 18.2 (2.3-51.8) | 63.6 (30.8-89.1) | 45.5 (16.7-76.6) |
| Indigestion | 45.5 (16.7-76.6) | 18.2 (2.3-51.8) | 9.1 (0.2-41.3) | 27.3 (6.0-61.0) | 54.5 (23.4-83.3) | 36.4 (10.9-69.2) |
| Nausea | 70.0 (34.8-93.3) | 10.0 (0.3-44.5) | 10.0 (0.3-44.5) | 10.0 (0.3-44.5) | 30.0 (6.7-65.2) | 20.0 (2.5-55.6) |
| Diarrhoea | 54.5 (23.4-83.3) | 9.1 (0.2-41.3) | 18.2 (2.3-51.8) | 18.2 (2.3-51.8) | 45.5 (16.7-76.6) | 36.4 (10.9-69.2) |
| Abdominal pain/cramps | 50.0 (18.7-81.3) | 0.0 (0.0-30.8) | 20.0 (2.5-55.6) | 30.0 (6.7-65.2) | 50.0 (18.7-81.3) | 50.0 (18.7-81.3) |
| Vomited | 72.7 (39.0-94.0) | 18.2 (2.3-51.8) | 9.1 (0.2-41.3) | 0.0 (0.0-28.5) | 27.3 (6.0-61.0) | 9.1 (0.2-41.3) |
| Obstipation | 60.0 (26.2-87.8) | 30.0 (6.7-65.2) | 0.0 (0.0-30.8) | 10.0 (0.3-44.5) | 40.0 (12.2-73.8) | 10.0 (0.3-44.5) |
| Flatulence | 0.0 (0.0-30.8) | 30.0 (6.7-65.2) | 30.0 (6.7-65.2) | 40.0 (12.2-73.8) | 100.0 (69.2-100.0) | 70.0 (34.8-93.3) |
| Muscle/joint cramps/pain (scale) | 18.2 (2.3-51.8) | Na | Na | Na | 81.8 (48.2-97.7) | Na |
| Frequent urination | 30.0 (6.7-65.2) | 50.0 (18.7-81.3) | 20.0 (2.5-55.6) | 0.0 (0.0-30.8) | 70.0 (34.8-93.3) | 20.0 (2.5-55.6) |
| Muscle cramps | 27.3 (6.0-61.0) | 36.4 (10.9-69.2) | 9.1 (0.2-41.3) | 27.3 (6.0-61.0) | 72.7 (39.0-94.0) | 36.4 (10.9-69.2) |
| Aches/pains muscles/joints | 30.0 (6.7-65.2) | 0.0 (0.0-30.8) | 30.0 (6.7-65.2) | 40.0 (12.2-73.8) | 70.0 (34.8-93.3) | 70.0 (34.8-93.3) |
| Muscle weakness | 36.4 (10.9-69.2) | 36.4 (10.9-69.2) | 18.2 (2.3-51.8) | 9.1 (0.2-41.3) | 63.6 (30.8-89.1) | 27.3 (6.0-61.0) |
| Pale/cold fingers/toes | 60.0 (14.7-94.7) | 0.0 (0.0-52.2) | 0.0 (0.0-52.2) | 40.0 (5.3-85.3) | 40.0 (5.3-85.3) | 40.0 (5.3-85.3) |
| Tingling/numbness hands/feet | 50.0 (18.7-81.3) | 40.0 (12.2-73.8) | 0.0 (0.0-30.8) | 10.0 (0.3-44.5) | 50.0 (18.7-81.3) | 10.0 (0.3-44.5) |
| Problems finger dexterity | 70.0 (34.8-93.3) | 30.0 (6.7-65.2) | 0.0 (0.0-30.8) | 0.0 (0.0-30.8) | 30.0 (6.7-65.2) | 0.0 (0.0-30.8) |
| Trouble walking pain | 27.3 (6.0-61.0) | 18.2 (2.3-51.8) | 27.3 (6.0-61.0) | 27.3 (6.0-61.0) | 72.7 (39.0-94.0) | 54.5 (23.4-83.3) |
| Back pain | 60.0 (14.7-94.7) | 20.0 (0.5-71.6) | 20.0 (0.5-71.6) | 0.0 (0.0-52.2) | 40.0 (5.3-85.3) | 20.0 (0.5-71.6) |
| Pain | 54.5 (23.4-83.3) | 36.4 (10.9-69.2) | 9.1 (0.2-41.3) | 0.0 (0.0-28.5) | 45.5 (16.7-76.6) | 9.1 (0.2-41.3) |
| Fever/chills | 72.7 (39.0-94.0) | 18.2 (2.3-51.8) | 9.1 (0.2-41.3) | 0.0 (0.0-28.5) | 27.3 (6.0-61.0) | 9.1 (0.2-41.3) |
| Sweating excessively | 63.6 (30.8-89.1) | 9.1 (0.2-41.3) | 27.3 (6.0-61.0) | 0.0 (0.0-28.5) | 36.4 (10.9-69.2) | 27.3 (6.0-61.0) |
| Problems tolerating heat/cold | 40.0 (12.2-73.8) | 40.0 (12.2-73.8) | 20.0 (2.5-55.6) | 0.0 (0.0-30.8) | 60.0 (26.2-87.8) | 20.0 (2.5-55.6) |
| Hot flushes | 60.0 (26.2-87.8) | 30.0 (6.7-65.2) | 10.0 (0.3-44.5) | 0.0 (0.0-30.8) | 40.0 (12.2-73.8) | 10.0 (0.3-44.5) |
| Airway infections | 100.0 (47.8-100.0) | 0.0 (0.0-52.2) | 0.0 (0.0-52.2) | 0.0 (0.0-52.2) | 0.0 (0.0-52.2) | 0.0 (0.0-52.2) |
| Other infections | 80.0 (28.4-99.5) | 0.0 (0.0-52.2) | 20.0 (0.5-71.6) | 0.0 (0.0-52.2) | 20.0 (0.5-71.6) | 20.0 (0.5-71.6) |
| Sore/enlarged nipples/breasts | 100.0 (71.5-100.0) | 0.0 (0.0-28.5) | 0.0 (0.0-28.5) | 0.0 (0.0-28.5) | 0.0 (0.0-28.5) | 0.0 (0.0-28.5) |
| Sexuality problems (scale) | 30.0 (6.7-65.2) | Na | Na | Na | 70.0 (34.8-93.3) | Na |
| Less interest sex | 30.0 (6.7-65.2) | 20.0 (2.5-55.6) | 30.0 (6.7-65.2) | 20.0 (2.5-55.6) | 70.0 (34.8-93.3) | 50.0 (18.7-81.3) |
| Less pleasure sex | 40.0 (12.2-73.8) | 40.0 (12.2-73.8) | 10.0 (0.3-44.5) | 10.0 (0.3-44.5) | 60.0 (26.2-87.8) | 20.0 (2.5-55.6) |
| Pain during/after sex | 70.0 (34.8-93.3) | 20.0 (2.5-55.6) | 10.0 (0.3-44.5) | 0.0 (0.0-30.8) | 30.0 (6.7-65.2) | 10.0 (0.3-44.5) |
| Difficult erection | 50.0 (1.3-98.7) | 0.0 (0.0-84.2) | 50.0 (1.3-98.7) | 0.0 (0.0-84.2) | 50.0 (1.3-98.7) | 50.0 (1.3-98.7) |
| Dry vagina | 37.5 (8.5-75.5) | 37.5 (8.5-75.5) | 25.0 (3.2-65.1) | 0.0 (0.0-36.9) | 62.5 (24.5-91.5) | 25.0 (3.2-65.1) |

* At T4 as not available at T1. $ Moderate-severe includes ‘Quite a bit’ and ‘A lot’. Abbreviations: Na: not applicable

Table 20 Prevalence (% with 95%confidence intervals) of symptom scores for seven scales plus 76 single items, in 70 patients on dasatinib on T1

| Item/scale | Dasatinib | | | | | |
| --- | --- | --- | --- | --- | --- | --- |
|  | Not at all | A little | Quite a bit | A lot | Any severity | Moderate-severe $ |
| Fatigue (scale) | 8.6 (3.2-17.7) | Na | Na | Na | 91.4 (82.3-96.8) | Na |
| Tired | 11.4 (5.1-21.3) | 28.6 (18.4-40.6) | 38.6 (27.2-51.0) | 21.4 (12.5-32.9) | 88.6 (78.7-94.9) | 60.0 (47.6-71.5) |
| Needed rest | 17.1 (9.2-28.0) | 38.6 (27.2-51.0) | 30.0 (19.6-42.1) | 14.3 (7.1-24.7) | 82.9 (72.0-90.8) | 44.3 (32.4-56.7) |
| Lacked energy | 16.4 (8.5-27.5) | 29.9 (19.3-42.3) | 32.8 (21.8-45.4) | 20.9 (11.9-32.6) | 83.6 (72.5-91.5) | 53.7 (41.1-66.0) |
| Felt lethargic | 19.7 (10.6-31.8) | 49.2 (36.1-62.3) | 18.0 (9.4-30.0) | 13.1 (5.8-24.2) | 80.3 (68.2-89.4) | 31.1 (19.9-44.3) |
| Felt weak | 29.5 (18.5-42.6) | 41.0 (28.6-54.3) | 16.4 (8.2-28.1) | 13.1 (5.8-24.2) | 70.5 (57.4-81.5) | 29.5 (18.5-42.6) |
| Sleepy | 21.3 (11.9-33.7) | 42.6 (30.0-55.9) | 26.2 (15.8-39.1) | 9.8 (3.7-20.2) | 78.7 (66.3-88.1) | 36.1 (24.2-49.4) |
| Headaches | 50.8 (37.5-64.1) | 32.2 (20.6-45.6) | 11.9 (4.9-22.9) | 5.1 (1.1-14.1) | 49.2 (35.9-62.5) | 16.9 (8.4-29.0) |
| Dizziness | 63.8 (50.1-76.0) | 29.3 (18.1-42.7) | 5.2 (1.1-14.4) | 1.7 (0.0-9.2) | 36.2 (24.0-49.9) | 6.9 (1.9-16.7) |
| Cognitive problems (scale) | 22.4 (12.5-35.3) | Na | Na | Na | 77.6 (64.7-87.5) | Na |
| Difficulty remembering | 31.0 (19.5-44.5) | 44.8 (31.7-58.5) | 19.0 (9.9-31.4) | 5.2 (1.1-14.4) | 69.0 (55.5-80.5) | 24.1 (13.9-37.2) |
| Difficulty concentrating | 45.6 (32.4-59.3) | 24.6 (14.1-37.8) | 22.8 (12.7-35.8) | 7.0 (1.9-17.0) | 54.4 (40.7-67.6) | 29.8 (18.4-43.4) |
| Trouble thinking clearly | 37.9 (25.5-51.6) | 43.1 (30.2-56.8) | 15.5 (7.3-27.4) | 3.4 (0.4-11.9) | 62.1 (48.4-74.5) | 19.0 (9.9-31.4) |
| Bruised easily | 70.2 (56.6-81.6) | 22.8 (12.7-35.8) | 5.3 (1.1-14.6) | 1.8 (0.0-9.4) | 29.8 (18.4-43.4) | 7.0 (1.9-17.0) |
| Skin problems (scale) | 14.3 (6.4-26.2) | Na | Na | Na | 85.7 (73.8-93.6) | Na |
| Skin colour change | 58.2 (44.1-71.3) | 21.8 (11.8-35.0) | 20.0 (10.4-33.0) | 0.0 (0.0-6.5) | 41.8 (28.7-55.9) | 20.0 (10.4-33.0) |
| Skin itched | 28.6 (17.3-42.2) | 35.7 (23.4-49.6) | 32.1 (20.3-46.0) | 3.6 (0.4-12.3) | 71.4 (57.8-82.7) | 35.7 (23.4-49.6) |
| Skin rash | 34.5 (22.2-48.6) | 40.0 (27.0-54.1) | 18.2 (9.1-30.9) | 7.3 (2.0-17.6) | 65.5 (51.4-77.8) | 25.5 (14.7-39.0) |
| Skin dry/flaky/cracked | 41.1 (28.1-55.0) | 32.1 (20.3-46.0) | 23.2 (13.0-36.4) | 3.6 (0.4-12.3) | 58.9 (45.0-71.9) | 26.8 (15.8-40.3) |
| Skin sore/painful | 51.8 (38.0-65.3) | 32.1 (20.3-46.0) | 14.3 (6.4-26.2) | 1.8 (0.0-9.6) | 48.2 (34.7-62.0) | 16.1 (7.6-28.3) |
| Wounds healing problems | 77.2 (64.2-87.3) | 14.0 (6.3-25.8) | 7.0 (1.9-17.0) | 1.8 (0.0-9.4) | 22.8 (12.7-35.8) | 8.8 (2.9-19.3) |
| Hair loss | 46.4 (33.0-60.3) | 28.6 (17.3-42.2) | 23.2 (13.0-36.4) | 1.8 (0.0-9.6) | 53.6 (39.7-67.0) | 25.0 (14.4-38.4) |
| Nail problems | 67.9 (56.4-78.1) | 20.5 (12.2-31.2) | 9.0 (3.7-17.6) | 2.6 (0.3-9.0) | 32.1 (21.9-43.6) | 11.5 (5.4-20.8) |
| Eye problems (scale) | 18.2 (9.1-30.9) | Na | Na | Na | 81.8 (69.1-90.9) | Na |
| Watery eyes | 61.1 (46.9-74.1) | 14.8 (6.6-27.1) | 18.5 (9.3-31.4) | 5.6 (1.2-15.4) | 38.9 (25.9-53.1) | 24.1 (13.5-37.6) |
| Burning eyes | 47.2 (33.3-61.4) | 24.5 (13.8-38.3) | 26.4 (15.3-40.3) | 1.9 (0.0-10.1) | 52.8 (38.6-66.7) | 28.3 (16.8-42.3) |
| Light sensitive eyes | 46.3 (32.6-60.4) | 25.9 (15.0-39.7) | 18.5 (9.3-31.4) | 9.3 (3.1-20.3) | 53.7 (39.6-67.4) | 27.8 (16.5-41.6) |
| Blurry eyes | 44.4 (30.9-58.6) | 37.0 (24.3-51.3) | 16.7 (7.9-29.3) | 1.9 (0.0-9.9) | 55.6 (41.4-69.1) | 18.5 (9.3-31.4) |
| Red eyes | 83.3 (70.7-92.1) | 13.0 (5.4-24.9) | 3.7 (0.5-12.7) | 0.0 (0.0-6.6) | 16.7 (7.9-29.3) | 3.7 (0.5-12.7) |
| Swelling face/eyes | 65.5 (51.4-77.8) | 16.4 (7.8-28.8) | 10.9 (4.1-22.2) | 7.3 (2.0-17.6) | 34.5 (22.2-48.6) | 18.2 (9.1-30.9) |
| Swelling body parts | 57.4 (43.2-70.8) | 29.6 (18.0-43.6) | 11.1 (4.2-22.6) | 1.9 (0.0-9.9) | 42.6 (29.2-56.8) | 13.0 (5.4-24.9) |
| Hearing problems | 80.4 (67.6-89.8) | 16.1 (7.6-28.3) | 3.6 (0.4-12.3) | 0.0 (0.0-6.4) | 19.6 (10.2-32.4) | 3.6 (0.4-12.3) |
| Nose bleeds | 91.1 (80.4-97.0) | 8.9 (3.0-19.6) | 0.0 (0.0-6.4) | 0.0 (0.0-6.4) | 8.9 (3.0-19.6) | 0.0 (0.0-6.4) |
| Nose problems | 50.0 (27.2-72.8) | 25.0 (8.7-49.1) | 25.0 (8.7-49.1) | 0.0 (0.0-16.8) | 50.0 (27.2-72.8) | 25.0 (8.7-49.1) |
| Dry mouth | 60.7 (46.8-73.5) | 26.8 (15.8-40.3) | 7.1 (2.0-17.3) | 5.4 (1.1-14.9) | 39.3 (26.5-53.2) | 12.5 (5.2-24.1) |
| Pain/soreness mouth | 76.8 (63.6-87.0) | 19.6 (10.2-32.4) | 1.8 (0.0-9.6) | 1.8 (0.0-9.6) | 23.2 (13.0-36.4) | 3.6 (0.4-12.3) |
| Teeth problems | 80.0 (67.0-89.6) | 14.5 (6.5-26.7) | 5.5 (1.1-15.1) | 0.0 (0.0-6.5) | 20.0 (10.4-33.0) | 5.5 (1.1-15.1) |
| Bleeding gums | 85.7 (73.8-93.6) | 10.7 (4.0-21.9) | 3.6 (0.4-12.3) | 0.0 (0.0-6.4) | 14.3 (6.4-26.2) | 3.6 (0.4-12.3) |
| Taste change | 80.0 (67.0-89.6) | 12.7 (5.3-24.5) | 5.5 (1.1-15.1) | 1.8 (0.0-9.7) | 20.0 (10.4-33.0) | 7.3 (2.0-17.6) |
| Appetite loss | 75.0 (61.6-85.6) | 17.9 (8.9-30.4) | 7.1 (2.0-17.3) | 0.0 (0.0-6.4) | 25.0 (14.4-38.4) | 7.1 (2.0-17.3) |
| Mood problems (scale) | 15.3 (7.2-27.0) | Na | Na | Na | 84.7 (73.0-92.8) | Na |
| Depressed | 52.7 (38.8-66.3) | 25.5 (14.7-39.0) | 16.4 (7.8-28.8) | 5.5 (1.1-15.1) | 47.3 (33.7-61.2) | 21.8 (11.8-35.0) |
| Irritable | 49.1 (35.4-62.9) | 32.7 (20.7-46.7) | 16.4 (7.8-28.8) | 1.8 (0.0-9.7) | 50.9 (37.1-64.6) | 18.2 (9.1-30.9) |
| Tense | 49.1 (35.4-62.9) | 38.2 (25.4-52.3) | 9.1 (3.0-20.0) | 3.6 (0.4-12.5) | 50.9 (37.1-64.6) | 12.7 (5.3-24.5) |
| Worried | 44.6 (31.3-58.5) | 37.5 (24.9-51.5) | 12.5 (5.2-24.1) | 5.4 (1.1-14.9) | 55.4 (41.5-68.7) | 17.9 (8.9-30.4) |
| Afraid | 78.2 (65.0-88.2) | 9.1 (3.0-20.0) | 9.1 (3.0-20.0) | 3.6 (0.4-12.5) | 21.8 (11.8-35.0) | 12.7 (5.3-24.5) |
| Insomnia | 44.1 (31.2-57.6) | 32.2 (20.6-45.6) | 13.6 (6.0-25.0) | 10.2 (3.8-20.8) | 55.9 (42.4-68.8) | 23.7 (13.6-36.6) |
| Palpitations | 53.6 (39.7-67.0) | 26.8 (15.8-40.3) | 16.1 (7.6-28.3) | 3.6 (0.4-12.3) | 46.4 (33.0-60.3) | 19.6 (10.2-32.4) |
| Pain in chest | 80.4 (67.6-89.8) | 17.9 (8.9-30.4) | 1.8 (0.0-9.6) | 0.0 (0.0-6.4) | 19.6 (10.2-32.4) | 1.8 (0.0-9.6) |
| Short of breath | 50.0 (36.3-63.7) | 39.3 (26.5-53.2) | 8.9 (3.0-19.6) | 1.8 (0.0-9.6) | 50.0 (36.3-63.7) | 10.7 (4.0-21.9) |
| Coughed | 73.2 (59.7-84.2) | 17.9 (8.9-30.4) | 5.4 (1.1-14.9) | 3.6 (0.4-12.3) | 26.8 (15.8-40.3) | 8.9 (3.0-19.6) |
| Feeling bloated | 37.5 (24.9-51.5) | 37.5 (24.9-51.5) | 19.6 (10.2-32.4) | 5.4 (1.1-14.9) | 62.5 (48.5-75.1) | 25.0 (14.4-38.4) |
| Indigestion | 63.6 (49.6-76.2) | 20.0 (10.4-33.0) | 10.9 (4.1-22.2) | 5.5 (1.1-15.1) | 36.4 (23.8-50.4) | 16.4 (7.8-28.8) |
| Nausea | 67.9 (54.0-79.7) | 19.6 (10.2-32.4) | 8.9 (3.0-19.6) | 3.6 (0.4-12.3) | 32.1 (20.3-46.0) | 12.5 (5.2-24.1) |
| Diarrhoea | 60.7 (46.8-73.5) | 32.1 (20.3-46.0) | 3.6 (0.4-12.3) | 3.6 (0.4-12.3) | 39.3 (26.5-53.2) | 7.1 (2.0-17.3) |
| Abdominal pain/cramps | 49.1 (35.4-62.9) | 36.4 (23.8-50.4) | 5.5 (1.1-15.1) | 9.1 (3.0-20.0) | 50.9 (37.1-64.6) | 14.5 (6.5-26.7) |
| Vomited | 92.9 (82.7-98.0) | 5.4 (1.1-14.9) | 1.8 (0.0-9.6) | 0.0 (0.0-6.4) | 7.1 (2.0-17.3) | 1.8 (0.0-9.6) |
| Obstipation | 57.1 (43.2-70.3) | 26.8 (15.8-40.3) | 7.1 (2.0-17.3) | 8.9 (3.0-19.6) | 42.9 (29.7-56.8) | 16.1 (7.6-28.3) |
| Flatulence | 40.0 (27.0-54.1) | 25.5 (14.7-39.0) | 25.5 (14.7-39.0) | 9.1 (3.0-20.0) | 60.0 (45.9-73.0) | 34.5 (22.2-48.6) |
| Muscle/joint cramps/pain (scale) | 18.2 (9.1-30.9) | Na | Na | Na | 81.8 (69.1-90.9) | Na |
| Frequent urination | 40.0 (27.0-54.1) | 25.5 (14.7-39.0) | 21.8 (11.8-35.0) | 12.7 (5.3-24.5) | 60.0 (45.9-73.0) | 34.5 (22.2-48.6) |
| Muscle cramps | 48.1 (34.3-62.2) | 35.2 (22.7-49.4) | 14.8 (6.6-27.1) | 1.9 (0.0-9.9) | 51.9 (37.8-65.7) | 16.7 (7.9-29.3) |
| Aches/pains muscles/joints | 23.6 (13.2-37.0) | 36.4 (23.8-50.4) | 27.3 (16.1-41.0) | 12.7 (5.3-24.5) | 76.4 (63.0-86.8) | 40.0 (27.0-54.1) |
| Muscle weakness | 60.0 (45.9-73.0) | 27.3 (16.1-41.0) | 9.1 (3.0-20.0) | 3.6 (0.4-12.5) | 40.0 (27.0-54.1) | 12.7 (5.3-24.5) |
| Pale/cold fingers/toes | 45.0 (23.1-68.5) | 10.0 (1.2-31.7) | 25.0 (8.7-49.1) | 20.0 (5.7-43.7) | 55.0 (31.5-76.9) | 45.0 (23.1-68.5) |
| Tingling/numbness hands/feet | 41.8 (28.7-55.9) | 25.5 (14.7-39.0) | 18.2 (9.1-30.9) | 14.5 (6.5-26.7) | 58.2 (44.1-71.3) | 32.7 (20.7-46.7) |
| Problems finger dexterity | 75.9 (62.4-86.5) | 14.8 (6.6-27.1) | 7.4 (2.1-17.9) | 1.9 (0.0-9.9) | 24.1 (13.5-37.6) | 9.3 (3.1-20.3) |
| Trouble walking pain | 67.3 (53.3-79.3) | 18.2 (9.1-30.9) | 10.9 (4.1-22.2) | 3.6 (0.4-12.5) | 32.7 (20.7-46.7) | 14.5 (6.5-26.7) |
| Back pain | 30.0 (11.9-54.3) | 45.0 (23.1-68.5) | 25.0 (8.7-49.1) | 0.0 (0.0-16.8) | 70.0 (45.7-88.1) | 25.0 (8.7-49.1) |
| Pain | 45.5 (32.0-59.4) | 36.4 (23.8-50.4) | 14.5 (6.5-26.7) | 3.6 (0.4-12.5) | 54.5 (40.6-68.0) | 18.2 (9.1-30.9) |
| Fever/chills | 72.2 (58.4-83.5) | 20.4 (10.6-33.5) | 7.4 (2.1-17.9) | 0.0 (0.0-6.6) | 27.8 (16.5-41.6) | 7.4 (2.1-17.9) |
| Sweating excessively | 67.3 (53.3-79.3) | 21.8 (11.8-35.0) | 10.9 (4.1-22.2) | 0.0 (0.0-6.5) | 32.7 (20.7-46.7) | 10.9 (4.1-22.2) |
| Problems tolerating heat/cold | 50.9 (37.1-64.6) | 36.4 (23.8-50.4) | 9.1 (3.0-20.0) | 3.6 (0.4-12.5) | 49.1 (35.4-62.9) | 12.7 (5.3-24.5) |
| Hot flushes | 65.5 (51.4-77.8) | 21.8 (11.8-35.0) | 9.1 (3.0-20.0) | 3.6 (0.4-12.5) | 34.5 (22.2-48.6) | 12.7 (5.3-24.5) |
| Airway infections | 85.0 (62.1-96.8) | 10.0 (1.2-31.7) | 5.0 (0.1-24.9) | 0.0 (0.0-16.8) | 15.0 (3.2-37.9) | 5.0 (0.1-24.9) |
| Other infections | 85.0 (62.1-96.8) | 10.0 (1.2-31.7) | 0.0 (0.0-16.8) | 5.0 (0.1-24.9) | 15.0 (3.2-37.9) | 5.0 (0.1-24.9) |
| Sore/enlarged nipples/breasts | 92.6 (82.1-97.9) | 7.4 (2.1-17.9) | 0.0 (0.0-6.6) | 0.0 (0.0-6.6) | 7.4 (2.1-17.9) | 0.0 (0.0-6.6) |
| Sexuality problems (scale) | 24.5 (13.8-38.3) | Na | Na | Na | 75.5 (61.7-86.2) | Na |
| Less interest sex | 32.1 (19.9-46.3) | 32.1 (19.9-46.3) | 22.6 (12.3-36.2) | 13.2 (5.5-25.3) | 67.9 (53.7-80.1) | 35.8 (23.1-50.2) |
| Less pleasure sex | 39.2 (25.8-53.9) | 37.3 (24.1-51.9) | 17.6 (8.4-30.9) | 5.9 (1.2-16.2) | 60.8 (46.1-74.2) | 23.5 (12.8-37.5) |
| Pain during/after sex | 76.5 (62.5-87.2) | 15.7 (7.0-28.6) | 2.0 (0.0-10.4) | 5.9 (1.2-16.2) | 23.5 (12.8-37.5) | 7.8 (2.2-18.9) |
| Difficult erection | 61.9 (38.4-81.9) | 28.6 (11.3-52.2) | 0.0 (0.0-16.1) | 9.5 (1.2-30.4) | 38.1 (18.1-61.6) | 9.5 (1.2-30.4) |
| Dry vagina | 51.9 (31.9-71.3) | 14.8 (4.2-33.7) | 22.2 (8.6-42.3) | 11.1 (2.4-29.2) | 48.1 (28.7-68.1) | 33.3 (16.5-54.0) |

* At T4 as not available at T1. $ Moderate-severe includes ‘Quite a bit’ and ‘A lot’. Abbreviations: Na: not applicable

Table 21 Prevalence (% with 95%confidence intervals) of scores for seven scales plus 76 single items, in 84 patients on imatinib on T1

| Item/scale | Imatinib | | | | | |
| --- | --- | --- | --- | --- | --- | --- |
|  | Not at all | A little | Quite a bit | A lot | Any severity | Moderate-severe $ |
| Fatigue (scale) | 10.7 (5.0-19.4) | Na | Na | Na | 89.3 (80.6-95.0) | Na |
| Tired | 13.1 (6.7-22.2) | 38.1 (27.7-49.3) | 32.1 (22.4-43.2) | 16.7 (9.4-26.4) | 86.9 (77.8-93.3) | 48.8 (37.7-60.0) |
| Needed rest | 23.8 (15.2-34.3) | 35.7 (25.6-46.9) | 34.5 (24.5-45.7) | 6.0 (2.0-13.3) | 76.2 (65.7-84.8) | 40.5 (29.9-51.7) |
| Lacked energy | 20.7 (12.6-31.1) | 31.7 (21.9-42.9) | 36.6 (26.2-48.0) | 11.0 (5.1-19.8) | 79.3 (68.9-87.4) | 47.6 (36.4-58.9) |
| Felt lethargic | 30.4 (20.5-41.8) | 35.4 (25.0-47.0) | 30.4 (20.5-41.8) | 3.8 (0.8-10.7) | 69.6 (58.2-79.5) | 34.2 (23.9-45.7) |
| Felt weak | 39.5 (28.4-51.4) | 36.8 (26.1-48.7) | 23.7 (14.7-34.8) | 0.0 (0.0-4.7) | 60.5 (48.6-71.6) | 23.7 (14.7-34.8) |
| Sleepy | 28.9 (19.1-40.5) | 44.7 (33.3-56.6) | 22.4 (13.6-33.4) | 3.9 (0.8-11.1) | 71.1 (59.5-80.9) | 26.3 (16.9-37.7) |
| Headaches | 70.3 (58.5-80.3) | 21.6 (12.9-32.7) | 8.1 (3.0-16.8) | 0.0 (0.0-4.9) | 29.7 (19.7-41.5) | 8.1 (3.0-16.8) |
| Dizziness | 68.9 (57.1-79.2) | 28.4 (18.5-40.1) | 2.7 (0.3-9.4) | 0.0 (0.0-4.9) | 31.1 (20.8-42.9) | 2.7 (0.3-9.4) |
| Cognitive problems (scale) | 33.8 (23.2-45.7) | Na | Na | Na | 66.2 (54.3-76.8) | Na |
| Difficulty remembering | 45.9 (34.3-57.9) | 44.6 (33.0-56.6) | 8.1 (3.0-16.8) | 1.4 (0.0-7.3) | 54.1 (42.1-65.7) | 9.5 (3.9-18.5) |
| Difficulty concentrating | 50.0 (38.1-61.9) | 33.8 (23.2-45.7) | 14.9 (7.7-25.0) | 1.4 (0.0-7.3) | 50.0 (38.1-61.9) | 16.2 (8.7-26.6) |
| Trouble thinking clearly | 54.2 (42.0-66.0) | 37.5 (26.4-49.7) | 6.9 (2.3-15.5) | 1.4 (0.0-7.5) | 45.8 (34.0-58.0) | 8.3 (3.1-17.3) |
| Bruised easily | 51.4 (39.3-63.3) | 33.3 (22.7-45.4) | 13.9 (6.9-24.1) | 1.4 (0.0-7.5) | 48.6 (36.7-60.7) | 15.3 (7.9-25.7) |
| Skin problems (scale) | 21.1 (12.3-32.4) | Na | Na | Na | 78.9 (67.6-87.7) | Na |
| Skin colour change | 61.4 (49.0-72.8) | 30.0 (19.6-42.1) | 5.7 (1.6-14.0) | 2.9 (0.3-9.9) | 38.6 (27.2-51.0) | 8.6 (3.2-17.7) |
| Skin itched | 46.5 (34.5-58.7) | 43.7 (31.9-56.0) | 5.6 (1.6-13.8) | 4.2 (0.9-11.9) | 53.5 (41.3-65.5) | 9.9 (4.1-19.3) |
| Skin rash | 64.8 (52.5-75.8) | 22.5 (13.5-34.0) | 11.3 (5.0-21.0) | 1.4 (0.0-7.6) | 35.2 (24.2-47.5) | 12.7 (6.0-22.7) |
| Skin dry/flaky/cracked | 48.6 (36.4-60.8) | 38.6 (27.2-51.0) | 11.4 (5.1-21.3) | 1.4 (0.0-7.7) | 51.4 (39.2-63.6) | 12.9 (6.1-23.0) |
| Skin sore/painful | 50.7 (38.6-62.8) | 39.4 (28.0-51.7) | 9.9 (4.1-19.3) | 0.0 (0.0-5.1) | 49.3 (37.2-61.4) | 9.9 (4.1-19.3) |
| Wounds healing problems | 73.6 (61.9-83.3) | 23.6 (14.4-35.1) | 1.4 (0.0-7.5) | 1.4 (0.0-7.5) | 26.4 (16.7-38.1) | 2.8 (0.3-9.7) |
| Hair loss | 72.2 (60.4-82.1) | 22.2 (13.3-33.6) | 4.2 (0.9-11.7) | 1.4 (0.0-7.5) | 27.8 (17.9-39.6) | 5.6 (1.5-13.6) |
| Nail problems | 67.9 (56.4-78.1) | 20.5 (12.2-31.2) | 9.0 (3.7-17.6) | 2.6 (0.3-9.0) | 32.1 (21.9-43.6) | 11.5 (5.4-20.8) |
| Eye problems (scale) | 11.3 (5.0-21.0) | Na | Na | Na | 88.7 (79.0-95.0) | Na |
| Watery eyes | 35.2 (24.2-47.5) | 29.6 (19.3-41.6) | 28.2 (18.1-40.1) | 7.0 (2.3-15.7) | 64.8 (52.5-75.8) | 35.2 (24.2-47.5) |
| Burning eyes | 35.2 (24.2-47.5) | 46.5 (34.5-58.7) | 16.9 (9.0-27.7) | 1.4 (0.0-7.6) | 64.8 (52.5-75.8) | 18.3 (10.1-29.3) |
| Light sensitive eyes | 35.7 (24.6-48.1) | 45.7 (33.7-58.1) | 14.3 (7.1-24.7) | 4.3 (0.9-12.0) | 64.3 (51.9-75.4) | 18.6 (10.3-29.7) |
| Blurry eyes | 40.0 (28.5-52.4) | 44.3 (32.4-56.7) | 15.7 (8.1-26.4) | 0.0 (0.0-5.1) | 60.0 (47.6-71.5) | 15.7 (8.1-26.4) |
| Red eyes | 60.0 (47.6-71.5) | 25.7 (16.0-37.6) | 11.4 (5.1-21.3) | 2.9 (0.3-9.9) | 40.0 (28.5-52.4) | 14.3 (7.1-24.7) |
| Swelling face/eyes | 30.6 (20.2-42.5) | 30.6 (20.2-42.5) | 30.6 (20.2-42.5) | 8.3 (3.1-17.3) | 69.4 (57.5-79.8) | 38.9 (27.6-51.1) |
| Swelling body parts | 52.9 (40.4-65.2) | 30.9 (20.2-43.3) | 13.2 (6.2-23.6) | 2.9 (0.4-10.2) | 47.1 (34.8-59.6) | 16.2 (8.4-27.1) |
| Hearing problems | 70.8 (58.9-81.0) | 19.4 (11.1-30.5) | 6.9 (2.3-15.5) | 2.8 (0.3-9.7) | 29.2 (19.0-41.1) | 9.7 (4.0-19.0) |
| Nose bleeds | 90.1 (80.7-95.9) | 9.9 (4.1-19.3) | 0.0 (0.0-5.1) | 0.0 (0.0-5.1) | 9.9 (4.1-19.3) | 0.0 (0.0-5.1) |
| Nose problems | 54.2 (32.8-74.4) | 29.2 (12.6-51.1) | 16.7 (4.7-37.4) | 0.0 (0.0-14.2) | 45.8 (25.6-67.2) | 16.7 (4.7-37.4) |
| Dry mouth | 52.9 (40.6-64.9) | 25.7 (16.0-37.6) | 14.3 (7.1-24.7) | 7.1 (2.4-15.9) | 47.1 (35.1-59.4) | 21.4 (12.5-32.9) |
| Pain/soreness mouth | 76.1 (64.5-85.4) | 19.7 (11.2-30.9) | 2.8 (0.3-9.8) | 1.4 (0.0-7.6) | 23.9 (14.6-35.5) | 4.2 (0.9-11.9) |
| Teeth problems | 70.4 (58.4-80.7) | 22.5 (13.5-34.0) | 4.2 (0.9-11.9) | 2.8 (0.3-9.8) | 29.6 (19.3-41.6) | 7.0 (2.3-15.7) |
| Bleeding gums | 73.2 (61.4-83.1) | 23.9 (14.6-35.5) | 2.8 (0.3-9.8) | 0.0 (0.0-5.1) | 26.8 (16.9-38.6) | 2.8 (0.3-9.8) |
| Taste change | 70.4 (58.4-80.7) | 18.3 (10.1-29.3) | 5.6 (1.6-13.8) | 5.6 (1.6-13.8) | 29.6 (19.3-41.6) | 11.3 (5.0-21.0) |
| Appetite loss | 67.1 (54.9-77.9) | 18.6 (10.3-29.7) | 10.0 (4.1-19.5) | 4.3 (0.9-12.0) | 32.9 (22.1-45.1) | 14.3 (7.1-24.7) |
| Mood problems (scale) | 21.3 (12.7-32.3) | Na | Na | Na | 78.7 (67.7-87.3) | Na |
| Depressed | 58.6 (46.2-70.2) | 24.3 (14.8-36.0) | 11.4 (5.1-21.3) | 5.7 (1.6-14.0) | 41.4 (29.8-53.8) | 17.1 (9.2-28.0) |
| Irritable | 55.7 (43.3-67.6) | 27.1 (17.2-39.1) | 11.4 (5.1-21.3) | 5.7 (1.6-14.0) | 44.3 (32.4-56.7) | 17.1 (9.2-28.0) |
| Tense | 52.9 (40.6-64.9) | 28.6 (18.4-40.6) | 11.4 (5.1-21.3) | 7.1 (2.4-15.9) | 47.1 (35.1-59.4) | 18.6 (10.3-29.7) |
| Worried | 40.0 (28.5-52.4) | 44.3 (32.4-56.7) | 12.9 (6.1-23.0) | 2.9 (0.3-9.9) | 60.0 (47.6-71.5) | 15.7 (8.1-26.4) |
| Afraid | 71.4 (59.4-81.6) | 18.6 (10.3-29.7) | 7.1 (2.4-15.9) | 2.9 (0.3-9.9) | 28.6 (18.4-40.6) | 10.0 (4.1-19.5) |
| Insomnia | 40.5 (29.3-52.6) | 28.4 (18.5-40.1) | 21.6 (12.9-32.7) | 9.5 (3.9-18.5) | 59.5 (47.4-70.7) | 31.1 (20.8-42.9) |
| Palpitations | 54.3 (41.9-66.3) | 31.4 (20.9-43.6) | 14.3 (7.1-24.7) | 0.0 (0.0-5.1) | 45.7 (33.7-58.1) | 14.3 (7.1-24.7) |
| Pain in chest | 79.4 (67.9-88.3) | 17.6 (9.5-28.8) | 1.5 (0.0-7.9) | 1.5 (0.0-7.9) | 20.6 (11.7-32.1) | 2.9 (0.4-10.2) |
| Short of breath | 44.3 (32.4-56.7) | 32.9 (22.1-45.1) | 20.0 (11.4-31.3) | 2.9 (0.3-9.9) | 55.7 (43.3-67.6) | 22.9 (13.7-34.4) |
| Coughed | 69.1 (56.7-79.8) | 23.5 (14.1-35.4) | 5.9 (1.6-14.4) | 1.5 (0.0-7.9) | 30.9 (20.2-43.3) | 7.4 (2.4-16.3) |
| Feeling bloated | 54.3 (41.9-66.3) | 27.1 (17.2-39.1) | 15.7 (8.1-26.4) | 2.9 (0.3-9.9) | 45.7 (33.7-58.1) | 18.6 (10.3-29.7) |
| Indigestion | 58.0 (45.5-69.8) | 23.2 (13.9-34.9) | 14.5 (7.2-25.0) | 4.3 (0.9-12.2) | 42.0 (30.2-54.5) | 18.8 (10.4-30.1) |
| Nausea | 48.5 (36.2-61.0) | 42.6 (30.7-55.2) | 8.8 (3.3-18.2) | 0.0 (0.0-5.3) | 51.5 (39.0-63.8) | 8.8 (3.3-18.2) |
| Diarrhoea | 47.8 (35.6-60.2) | 24.6 (15.1-36.5) | 14.5 (7.2-25.0) | 13.0 (6.1-23.3) | 52.2 (39.8-64.4) | 27.5 (17.5-39.6) |
| Abdominal pain/cramps | 49.3 (37.0-61.6) | 36.2 (25.0-48.7) | 14.5 (7.2-25.0) | 0.0 (0.0-5.2) | 50.7 (38.4-63.0) | 14.5 (7.2-25.0) |
| Vomited | 78.3 (66.7-87.3) | 20.3 (11.6-31.7) | 1.4 (0.0-7.8) | 0.0 (0.0-5.2) | 21.7 (12.7-33.3) | 1.4 (0.0-7.8) |
| Obstipation | 78.3 (66.7-87.3) | 17.4 (9.3-28.4) | 4.3 (0.9-12.2) | 0.0 (0.0-5.2) | 21.7 (12.7-33.3) | 4.3 (0.9-12.2) |
| Flatulence | 27.9 (17.7-40.1) | 41.2 (29.4-53.8) | 23.5 (14.1-35.4) | 7.4 (2.4-16.3) | 72.1 (59.9-82.3) | 30.9 (20.2-43.3) |
| Muscle/joint cramps/pain (scale) | 10.3 (4.2-20.1) | Na | Na | Na | 89.7 (79.9-95.8) | Na |
| Frequent urination | 42.6 (30.7-55.2) | 29.4 (19.0-41.7) | 23.5 (14.1-35.4) | 4.4 (0.9-12.4) | 57.4 (44.8-69.3) | 27.9 (17.7-40.1) |
| Muscle cramps | 25.0 (15.3-37.0) | 35.3 (24.1-47.8) | 23.5 (14.1-35.4) | 16.2 (8.4-27.1) | 75.0 (63.0-84.7) | 39.7 (28.0-52.3) |
| Aches/pains muscles/joints | 23.5 (14.1-35.4) | 30.9 (20.2-43.3) | 33.8 (22.8-46.3) | 11.8 (5.2-21.9) | 76.5 (64.6-85.9) | 45.6 (33.5-58.1) |
| Muscle weakness | 52.2 (39.7-64.6) | 28.4 (18.0-40.7) | 19.4 (10.8-30.9) | 0.0 (0.0-5.4) | 47.8 (35.4-60.3) | 19.4 (10.8-30.9) |
| Pale/cold fingers/toes | 54.2 (32.8-74.4) | 20.8 (7.1-42.2) | 16.7 (4.7-37.4) | 8.3 (1.0-27.0) | 45.8 (25.6-67.2) | 25.0 (9.8-46.7) |
| Tingling/numbness hands/feet | 45.6 (33.5-58.1) | 33.8 (22.8-46.3) | 16.2 (8.4-27.1) | 4.4 (0.9-12.4) | 54.4 (41.9-66.5) | 20.6 (11.7-32.1) |
| Problems finger dexterity | 67.6 (55.2-78.5) | 25.0 (15.3-37.0) | 4.4 (0.9-12.4) | 2.9 (0.4-10.2) | 32.4 (21.5-44.8) | 7.4 (2.4-16.3) |
| Trouble walking pain | 60.3 (47.7-72.0) | 23.5 (14.1-35.4) | 7.4 (2.4-16.3) | 8.8 (3.3-18.2) | 39.7 (28.0-52.3) | 16.2 (8.4-27.1) |
| Back pain | 58.3 (36.6-77.9) | 37.5 (18.8-59.4) | 0.0 (0.0-14.2) | 4.2 (0.1-21.1) | 41.7 (22.1-63.4) | 4.2 (0.1-21.1) |
| Pain | 47.1 (34.8-59.6) | 38.2 (26.7-50.8) | 10.3 (4.2-20.1) | 4.4 (0.9-12.4) | 52.9 (40.4-65.2) | 14.7 (7.3-25.4) |
| Fever/chills | 77.9 (66.2-87.1) | 19.1 (10.6-30.5) | 1.5 (0.0-7.9) | 1.5 (0.0-7.9) | 22.1 (12.9-33.8) | 2.9 (0.4-10.2) |
| Sweating excessively | 61.8 (49.2-73.3) | 26.5 (16.5-38.6) | 10.3 (4.2-20.1) | 1.5 (0.0-7.9) | 38.2 (26.7-50.8) | 11.8 (5.2-21.9) |
| Problems tolerating heat/cold | 52.9 (40.4-65.2) | 33.8 (22.8-46.3) | 8.8 (3.3-18.2) | 4.4 (0.9-12.4) | 47.1 (34.8-59.6) | 13.2 (6.2-23.6) |
| Hot flushes | 82.4 (71.2-90.5) | 14.7 (7.3-25.4) | 1.5 (0.0-7.9) | 1.5 (0.0-7.9) | 17.6 (9.5-28.8) | 2.9 (0.4-10.2) |
| Airway infections | 66.7 (44.7-84.4) | 16.7 (4.7-37.4) | 4.2 (0.1-21.1) | 12.5 (2.7-32.4) | 33.3 (15.6-55.3) | 16.7 (4.7-37.4) |
| Other infections | 87.5 (67.6-97.3) | 4.2 (0.1-21.1) | 4.2 (0.1-21.1) | 4.2 (0.1-21.1) | 12.5 (2.7-32.4) | 8.3 (1.0-27.0) |
| Sore/enlarged nipples/breasts | 86.6 (76.0-93.7) | 13.4 (6.3-24.0) | 0.0 (0.0-5.4) | 0.0 (0.0-5.4) | 13.4 (6.3-24.0) | 0.0 (0.0-5.4) |
| Sexuality problems (scale) | 40.0 (28.0-52.9) | Na | Na | Na | 60.0 (47.1-72.0) | Na |
| Less interest sex | 43.1 (30.8-56.0) | 24.6 (14.8-36.9) | 20.0 (11.1-31.8) | 12.3 (5.5-22.8) | 56.9 (44.0-69.2) | 32.3 (21.2-45.1) |
| Less pleasure sex | 53.1 (40.2-65.7) | 17.2 (8.9-28.7) | 20.3 (11.3-32.2) | 9.4 (3.5-19.3) | 46.9 (34.3-59.8) | 29.7 (18.9-42.4) |
| Pain during/after sex | 77.8 (65.5-87.3) | 15.9 (7.9-27.3) | 3.2 (0.4-11.0) | 3.2 (0.4-11.0) | 22.2 (12.7-34.5) | 6.3 (1.8-15.5) |
| Difficult erection | 61.8 (43.6-77.8) | 23.5 (10.7-41.2) | 11.8 (3.3-27.5) | 2.9 (0.1-15.3) | 38.2 (22.2-56.4) | 14.7 (5.0-31.1) |
| Dry vagina | 55.2 (35.7-73.6) | 20.7 (8.0-39.7) | 20.7 (8.0-39.7) | 3.4 (0.1-17.8) | 44.8 (26.4-64.3) | 24.1 (10.3-43.5) |

* At T4 as not available at T1. $ Moderate-severe includes ‘Quite a bit’ and ‘A lot’. Abbreviations: Na: not applicable

Table 22 Prevalence (% with 95%confidence intervals) of symptom scores for seven scales plus 76 single items, in 39 patients on nilotinib on T1

| Item/scale | Nilotinib | | | | | |
| --- | --- | --- | --- | --- | --- | --- |
|  | Not at all | A little | Quite a bit | A lot | Any severity | Moderate-severe $ |
| Fatigue (scale) | 5.1 (0.6-17.3) | Na | Na | Na | 94.9 (82.7-99.4) | Na |
| Tired | 12.8 (4.3-27.4) | 41.0 (25.6-57.9) | 38.5 (23.4-55.4) | 7.7 (1.6-20.9) | 87.2 (72.6-95.7) | 46.2 (30.1-62.8) |
| Needed rest | 12.8 (4.3-27.4) | 38.5 (23.4-55.4) | 43.6 (27.8-60.4) | 5.1 (0.6-17.3) | 87.2 (72.6-95.7) | 48.7 (32.4-65.2) |
| Lacked energy | 17.9 (7.5-33.5) | 38.5 (23.4-55.4) | 35.9 (21.2-52.8) | 7.7 (1.6-20.9) | 82.1 (66.5-92.5) | 43.6 (27.8-60.4) |
| Felt lethargic | 43.2 (27.1-60.5) | 35.1 (20.2-52.5) | 21.6 (9.8-38.2) | 0.0 (0.0-9.5) | 56.8 (39.5-72.9) | 21.6 (9.8-38.2) |
| Felt weak | 36.8 (21.8-54.0) | 44.7 (28.6-61.7) | 18.4 (7.7-34.3) | 0.0 (0.0-9.3) | 63.2 (46.0-78.2) | 18.4 (7.7-34.3) |
| Sleepy | 21.6 (9.8-38.2) | 40.5 (24.8-57.9) | 37.8 (22.5-55.2) | 0.0 (0.0-9.5) | 78.4 (61.8-90.2) | 37.8 (22.5-55.2) |
| Headaches | 80.6 (64.0-91.8) | 16.7 (6.4-32.8) | 2.8 (0.1-14.5) | 0.0 (0.0-9.7) | 19.4 (8.2-36.0) | 2.8 (0.1-14.5) |
| Dizziness | 69.4 (51.9-83.7) | 25.0 (12.1-42.2) | 5.6 (0.7-18.7) | 0.0 (0.0-9.7) | 30.6 (16.3-48.1) | 5.6 (0.7-18.7) |
| Cognitive problems (scale) | 40.0 (23.9-57.9) | Na | Na | Na | 60.0 (42.1-76.1) | Na |
| Difficulty remembering | 48.6 (31.4-66.0) | 45.7 (28.8-63.4) | 5.7 (0.7-19.2) | 0.0 (0.0-10.0) | 51.4 (34.0-68.6) | 5.7 (0.7-19.2) |
| Difficulty concentrating | 57.1 (39.4-73.7) | 37.1 (21.5-55.1) | 5.7 (0.7-19.2) | 0.0 (0.0-10.0) | 42.9 (26.3-60.6) | 5.7 (0.7-19.2) |
| Trouble thinking clearly | 60.0 (42.1-76.1) | 37.1 (21.5-55.1) | 2.9 (0.1-14.9) | 0.0 (0.0-10.0) | 40.0 (23.9-57.9) | 2.9 (0.1-14.9) |
| Bruised easily | 71.4 (53.7-85.4) | 28.6 (14.6-46.3) | 0.0 (0.0-10.0) | 0.0 (0.0-10.0) | 28.6 (14.6-46.3) | 0.0 (0.0-10.0) |
| Skin problems (scale) | 17.1 (6.6-33.6) | Na | Na | Na | 82.9 (66.4-93.4) | Na |
| Skin colour change | 77.1 (59.9-89.6) | 20.0 (8.4-36.9) | 2.9 (0.1-14.9) | 0.0 (0.0-10.0) | 22.9 (10.4-40.1) | 2.9 (0.1-14.9) |
| Skin itched | 37.1 (21.5-55.1) | 22.9 (10.4-40.1) | 20.0 (8.4-36.9) | 20.0 (8.4-36.9) | 62.9 (44.9-78.5) | 40.0 (23.9-57.9) |
| Skin rash | 50.0 (32.4-67.6) | 29.4 (15.1-47.5) | 17.6 (6.8-34.5) | 2.9 (0.1-15.3) | 50.0 (32.4-67.6) | 20.6 (8.7-37.9) |
| Skin dry/flaky/cracked | 34.3 (19.1-52.2) | 34.3 (19.1-52.2) | 22.9 (10.4-40.1) | 8.6 (1.8-23.1) | 65.7 (47.8-80.9) | 31.4 (16.9-49.3) |
| Skin sore/painful | 74.3 (56.7-87.5) | 17.1 (6.6-33.6) | 5.7 (0.7-19.2) | 2.9 (0.1-14.9) | 25.7 (12.5-43.3) | 8.6 (1.8-23.1) |
| Wounds healing problems | 97.1 (85.1-99.9) | 2.9 (0.1-14.9) | 0.0 (0.0-10.0) | 0.0 (0.0-10.0) | 2.9 (0.1-14.9) | 0.0 (0.0-10.0) |
| Hair loss | 50.0 (32.4-67.6) | 35.3 (19.7-53.5) | 5.9 (0.7-19.7) | 8.8 (1.9-23.7) | 50.0 (32.4-67.6) | 14.7 (5.0-31.1) |
| Nail problems | 67.9 (56.4-78.1) | 20.5 (12.2-31.2) | 9.0 (3.7-17.6) | 2.6 (0.3-9.0) | 32.1 (21.9-43.6) | 11.5 (5.4-20.8) |
| Eye problems (scale) | 8.8 (1.9-23.7) | Na | Na | Na | 91.2 (76.3-98.1) | Na |
| Watery eyes | 45.5 (28.1-63.6) | 45.5 (28.1-63.6) | 9.1 (1.9-24.3) | 0.0 (0.0-10.6) | 54.5 (36.4-71.9) | 9.1 (1.9-24.3) |
| Burning eyes | 42.4 (25.5-60.8) | 45.5 (28.1-63.6) | 9.1 (1.9-24.3) | 3.0 (0.1-15.8) | 57.6 (39.2-74.5) | 12.1 (3.4-28.2) |
| Light sensitive eyes | 40.6 (23.7-59.4) | 31.2 (16.1-50.0) | 25.0 (11.5-43.4) | 3.1 (0.1-16.2) | 59.4 (40.6-76.3) | 28.1 (13.7-46.7) |
| Blurry eyes | 42.4 (25.5-60.8) | 45.5 (28.1-63.6) | 9.1 (1.9-24.3) | 3.0 (0.1-15.8) | 57.6 (39.2-74.5) | 12.1 (3.4-28.2) |
| Red eyes | 84.8 (68.1-94.9) | 9.1 (1.9-24.3) | 6.1 (0.7-20.2) | 0.0 (0.0-10.6) | 15.2 (5.1-31.9) | 6.1 (0.7-20.2) |
| Swelling face/eyes | 73.5 (55.6-87.1) | 17.6 (6.8-34.5) | 8.8 (1.9-23.7) | 0.0 (0.0-10.3) | 26.5 (12.9-44.4) | 8.8 (1.9-23.7) |
| Swelling body parts | 84.4 (67.2-94.7) | 9.4 (2.0-25.0) | 6.2 (0.8-20.8) | 0.0 (0.0-10.9) | 15.6 (5.3-32.8) | 6.2 (0.8-20.8) |
| Hearing problems | 81.8 (64.5-93.0) | 12.1 (3.4-28.2) | 3.0 (0.1-15.8) | 3.0 (0.1-15.8) | 18.2 (7.0-35.5) | 6.1 (0.7-20.2) |
| Nose bleeds | 84.8 (68.1-94.9) | 12.1 (3.4-28.2) | 0.0 (0.0-10.6) | 3.0 (0.1-15.8) | 15.2 (5.1-31.9) | 3.0 (0.1-15.8) |
| Nose problems | 64.3 (35.1-87.2) | 14.3 (1.8-42.8) | 21.4 (4.7-50.8) | 0.0 (0.0-23.2) | 35.7 (12.8-64.9) | 21.4 (4.7-50.8) |
| Dry mouth | 63.6 (45.1-79.6) | 24.2 (11.1-42.3) | 6.1 (0.7-20.2) | 6.1 (0.7-20.2) | 36.4 (20.4-54.9) | 12.1 (3.4-28.2) |
| Pain/soreness mouth | 87.9 (71.8-96.6) | 3.0 (0.1-15.8) | 9.1 (1.9-24.3) | 0.0 (0.0-10.6) | 12.1 (3.4-28.2) | 9.1 (1.9-24.3) |
| Teeth problems | 90.9 (75.7-98.1) | 6.1 (0.7-20.2) | 3.0 (0.1-15.8) | 0.0 (0.0-10.6) | 9.1 (1.9-24.3) | 3.0 (0.1-15.8) |
| Bleeding gums | 84.4 (67.2-94.7) | 15.6 (5.3-32.8) | 0.0 (0.0-10.9) | 0.0 (0.0-10.9) | 15.6 (5.3-32.8) | 0.0 (0.0-10.9) |
| Taste change | 81.8 (64.5-93.0) | 15.2 (5.1-31.9) | 3.0 (0.1-15.8) | 0.0 (0.0-10.6) | 18.2 (7.0-35.5) | 3.0 (0.1-15.8) |
| Appetite loss | 84.8 (68.1-94.9) | 9.1 (1.9-24.3) | 6.1 (0.7-20.2) | 0.0 (0.0-10.6) | 15.2 (5.1-31.9) | 6.1 (0.7-20.2) |
| Mood problems (scale) | 21.6 (9.8-38.2) | Na | Na | Na | 78.4 (61.8-90.2) | Na |
| Depressed | 75.8 (57.7-88.9) | 21.2 (9.0-38.9) | 3.0 (0.1-15.8) | 0.0 (0.0-10.6) | 24.2 (11.1-42.3) | 3.0 (0.1-15.8) |
| Irritable | 60.6 (42.1-77.1) | 33.3 (18.0-51.8) | 6.1 (0.7-20.2) | 0.0 (0.0-10.6) | 39.4 (22.9-57.9) | 6.1 (0.7-20.2) |
| Tense | 63.6 (45.1-79.6) | 33.3 (18.0-51.8) | 3.0 (0.1-15.8) | 0.0 (0.0-10.6) | 36.4 (20.4-54.9) | 3.0 (0.1-15.8) |
| Worried | 48.5 (30.8-66.5) | 45.5 (28.1-63.6) | 6.1 (0.7-20.2) | 0.0 (0.0-10.6) | 51.5 (33.5-69.2) | 6.1 (0.7-20.2) |
| Afraid | 81.2 (63.6-92.8) | 18.8 (7.2-36.4) | 0.0 (0.0-10.9) | 0.0 (0.0-10.9) | 18.8 (7.2-36.4) | 0.0 (0.0-10.9) |
| Insomnia | 43.2 (27.1-60.5) | 29.7 (15.9-47.0) | 24.3 (11.8-41.2) | 2.7 (0.1-14.2) | 56.8 (39.5-72.9) | 27.0 (13.8-44.1) |
| Palpitations | 69.7 (51.3-84.4) | 18.2 (7.0-35.5) | 9.1 (1.9-24.3) | 3.0 (0.1-15.8) | 30.3 (15.6-48.7) | 12.1 (3.4-28.2) |
| Pain in chest | 87.9 (71.8-96.6) | 6.1 (0.7-20.2) | 6.1 (0.7-20.2) | 0.0 (0.0-10.6) | 12.1 (3.4-28.2) | 6.1 (0.7-20.2) |
| Short of breath | 66.7 (48.2-82.0) | 24.2 (11.1-42.3) | 9.1 (1.9-24.3) | 0.0 (0.0-10.6) | 33.3 (18.0-51.8) | 9.1 (1.9-24.3) |
| Coughed | 63.6 (45.1-79.6) | 24.2 (11.1-42.3) | 12.1 (3.4-28.2) | 0.0 (0.0-10.6) | 36.4 (20.4-54.9) | 12.1 (3.4-28.2) |
| Feeling bloated | 66.7 (48.2-82.0) | 24.2 (11.1-42.3) | 9.1 (1.9-24.3) | 0.0 (0.0-10.6) | 33.3 (18.0-51.8) | 9.1 (1.9-24.3) |
| Indigestion | 75.8 (57.7-88.9) | 15.2 (5.1-31.9) | 9.1 (1.9-24.3) | 0.0 (0.0-10.6) | 24.2 (11.1-42.3) | 9.1 (1.9-24.3) |
| Nausea | 75.0 (56.6-88.5) | 18.8 (7.2-36.4) | 6.2 (0.8-20.8) | 0.0 (0.0-10.9) | 25.0 (11.5-43.4) | 6.2 (0.8-20.8) |
| Diarrhoea | 84.4 (67.2-94.7) | 12.5 (3.5-29.0) | 0.0 (0.0-10.9) | 3.1 (0.1-16.2) | 15.6 (5.3-32.8) | 3.1 (0.1-16.2) |
| Abdominal pain/cramps | 65.6 (46.8-81.4) | 21.9 (9.3-40.0) | 9.4 (2.0-25.0) | 3.1 (0.1-16.2) | 34.4 (18.6-53.2) | 12.5 (3.5-29.0) |
| Vomited | 93.8 (79.2-99.2) | 6.2 (0.8-20.8) | 0.0 (0.0-10.9) | 0.0 (0.0-10.9) | 6.2 (0.8-20.8) | 0.0 (0.0-10.9) |
| Obstipation | 68.8 (50.0-83.9) | 15.6 (5.3-32.8) | 6.2 (0.8-20.8) | 9.4 (2.0-25.0) | 31.2 (16.1-50.0) | 15.6 (5.3-32.8) |
| Flatulence | 40.6 (23.7-59.4) | 25.0 (11.5-43.4) | 31.2 (16.1-50.0) | 3.1 (0.1-16.2) | 59.4 (40.6-76.3) | 34.4 (18.6-53.2) |
| Muscle/joint cramps/pain (scale) | 25.0 (11.5-43.4) | Na | Na | Na | 75.0 (56.6-88.5) | Na |
| Frequent urination | 53.1 (34.7-70.9) | 18.8 (7.2-36.4) | 25.0 (11.5-43.4) | 3.1 (0.1-16.2) | 46.9 (29.1-65.3) | 28.1 (13.7-46.7) |
| Muscle cramps | 34.4 (18.6-53.2) | 43.8 (26.4-62.3) | 15.6 (5.3-32.8) | 6.2 (0.8-20.8) | 65.6 (46.8-81.4) | 21.9 (9.3-40.0) |
| Aches/pains muscles/joints | 40.6 (23.7-59.4) | 25.0 (11.5-43.4) | 25.0 (11.5-43.4) | 9.4 (2.0-25.0) | 59.4 (40.6-76.3) | 34.4 (18.6-53.2) |
| Muscle weakness | 68.8 (50.0-83.9) | 28.1 (13.7-46.7) | 3.1 (0.1-16.2) | 0.0 (0.0-10.9) | 31.2 (16.1-50.0) | 3.1 (0.1-16.2) |
| Pale/cold fingers/toes | 78.6 (49.2-95.3) | 21.4 (4.7-50.8) | 0.0 (0.0-23.2) | 0.0 (0.0-23.2) | 21.4 (4.7-50.8) | 0.0 (0.0-23.2) |
| Tingling/numbness hands/feet | 56.2 (37.7-73.6) | 37.5 (21.1-56.3) | 6.2 (0.8-20.8) | 0.0 (0.0-10.9) | 43.8 (26.4-62.3) | 6.2 (0.8-20.8) |
| Problems finger dexterity | 81.2 (63.6-92.8) | 18.8 (7.2-36.4) | 0.0 (0.0-10.9) | 0.0 (0.0-10.9) | 18.8 (7.2-36.4) | 0.0 (0.0-10.9) |
| Trouble walking pain | 54.8 (36.0-72.7) | 35.5 (19.2-54.6) | 6.5 (0.8-21.4) | 3.2 (0.1-16.7) | 45.2 (27.3-64.0) | 9.7 (2.0-25.8) |
| Back pain | 42.9 (17.7-71.1) | 35.7 (12.8-64.9) | 7.1 (0.2-33.9) | 14.3 (1.8-42.8) | 57.1 (28.9-82.3) | 21.4 (4.7-50.8) |
| Pain | 62.5 (43.7-78.9) | 21.9 (9.3-40.0) | 12.5 (3.5-29.0) | 3.1 (0.1-16.2) | 37.5 (21.1-56.3) | 15.6 (5.3-32.8) |
| Fever/chills | 87.5 (71.0-96.5) | 12.5 (3.5-29.0) | 0.0 (0.0-10.9) | 0.0 (0.0-10.9) | 12.5 (3.5-29.0) | 0.0 (0.0-10.9) |
| Sweating excessively | 53.1 (34.7-70.9) | 28.1 (13.7-46.7) | 15.6 (5.3-32.8) | 3.1 (0.1-16.2) | 46.9 (29.1-65.3) | 18.8 (7.2-36.4) |
| Problems tolerating heat/cold | 71.9 (53.3-86.3) | 15.6 (5.3-32.8) | 9.4 (2.0-25.0) | 3.1 (0.1-16.2) | 28.1 (13.7-46.7) | 12.5 (3.5-29.0) |
| Hot flushes | 78.1 (60.0-90.7) | 9.4 (2.0-25.0) | 6.2 (0.8-20.8) | 6.2 (0.8-20.8) | 21.9 (9.3-40.0) | 12.5 (3.5-29.0) |
| Airway infections | 85.7 (57.2-98.2) | 14.3 (1.8-42.8) | 0.0 (0.0-23.2) | 0.0 (0.0-23.2) | 14.3 (1.8-42.8) | 0.0 (0.0-23.2) |
| Other infections | 92.9 (66.1-99.8) | 7.1 (0.2-33.9) | 0.0 (0.0-23.2) | 0.0 (0.0-23.2) | 7.1 (0.2-33.9) | 0.0 (0.0-23.2) |
| Sore/enlarged nipples/breasts | 87.5 (71.0-96.5) | 9.4 (2.0-25.0) | 3.1 (0.1-16.2) | 0.0 (0.0-10.9) | 12.5 (3.5-29.0) | 3.1 (0.1-16.2) |
| Sexuality problems (scale) | 35.5 (19.2-54.6) | Na | Na | Na | 64.5 (45.4-80.8) | Na |
| Less interest sex | 35.5 (19.2-54.6) | 32.3 (16.7-51.4) | 22.6 (9.6-41.1) | 9.7 (2.0-25.8) | 64.5 (45.4-80.8) | 32.3 (16.7-51.4) |
| Less pleasure sex | 45.2 (27.3-64.0) | 25.8 (11.9-44.6) | 16.1 (5.5-33.7) | 12.9 (3.6-29.8) | 54.8 (36.0-72.7) | 29.0 (14.2-48.0) |
| Pain during/after sex | 80.0 (61.4-92.3) | 10.0 (2.1-26.5) | 10.0 (2.1-26.5) | 0.0 (0.0-11.6) | 20.0 (7.7-38.6) | 10.0 (2.1-26.5) |
| Difficult erection | 25.0 (5.5-57.2) | 41.7 (15.2-72.3) | 16.7 (2.1-48.4) | 16.7 (2.1-48.4) | 75.0 (42.8-94.5) | 33.3 (9.9-65.1) |
| Dry vagina | 35.3 (14.2-61.7) | 41.2 (18.4-67.1) | 11.8 (1.5-36.4) | 11.8 (1.5-36.4) | 64.7 (38.3-85.8) | 23.5 (6.8-49.9) |

* At T4 as not available at T1. $ Moderate-severe includes ‘Quite a bit’ and ‘A lot’. Abbreviations: Na: not applicable

Table 23 Prevalence (% with 95%confidence intervals) of symptom scores for seven scales plus 76 single items, in 8 patients on ponatinib on T1

| Item/scale | Ponatinib | | | | | |
| --- | --- | --- | --- | --- | --- | --- |
|  | Not at all | A little | Quite a bit | A lot | Any severity | Moderate-severe $ |
| Fatigue (scale) | 12.5 (0.3-52.7) | Na | Na | Na | 87.5 (47.3-99.7) | Na |
| Tired | 12.5 (0.3-52.7) | 37.5 (8.5-75.5) | 25.0 (3.2-65.1) | 25.0 (3.2-65.1) | 87.5 (47.3-99.7) | 50.0 (15.7-84.3) |
| Needed rest | 12.5 (0.3-52.7) | 50.0 (15.7-84.3) | 37.5 (8.5-75.5) | 0.0 (0.0-36.9) | 87.5 (47.3-99.7) | 37.5 (8.5-75.5) |
| Lacked energy | 14.3 (0.4-57.9) | 42.9 (9.9-81.6) | 28.6 (3.7-71.0) | 14.3 (0.4-57.9) | 85.7 (42.1-99.6) | 42.9 (9.9-81.6) |
| Felt lethargic | 37.5 (8.5-75.5) | 50.0 (15.7-84.3) | 12.5 (0.3-52.7) | 0.0 (0.0-36.9) | 62.5 (24.5-91.5) | 12.5 (0.3-52.7) |
| Felt weak | 37.5 (8.5-75.5) | 37.5 (8.5-75.5) | 25.0 (3.2-65.1) | 0.0 (0.0-36.9) | 62.5 (24.5-91.5) | 25.0 (3.2-65.1) |
| Sleepy | 14.3 (0.4-57.9) | 28.6 (3.7-71.0) | 57.1 (18.4-90.1) | 0.0 (0.0-41.0) | 85.7 (42.1-99.6) | 57.1 (18.4-90.1) |
| Headaches | 85.7 (42.1-99.6) | 14.3 (0.4-57.9) | 0.0 (0.0-41.0) | 0.0 (0.0-41.0) | 14.3 (0.4-57.9) | 0.0 (0.0-41.0) |
| Dizziness | 71.4 (29.0-96.3) | 28.6 (3.7-71.0) | 0.0 (0.0-41.0) | 0.0 (0.0-41.0) | 28.6 (3.7-71.0) | 0.0 (0.0-41.0) |
| Cognitive problems (scale) | 14.3 (0.4-57.9) | Na | Na | Na | 85.7 (42.1-99.6) | Na |
| Difficulty remembering | 28.6 (3.7-71.0) | 57.1 (18.4-90.1) | 14.3 (0.4-57.9) | 0.0 (0.0-41.0) | 71.4 (29.0-96.3) | 14.3 (0.4-57.9) |
| Difficulty concentrating | 14.3 (0.4-57.9) | 71.4 (29.0-96.3) | 14.3 (0.4-57.9) | 0.0 (0.0-41.0) | 85.7 (42.1-99.6) | 14.3 (0.4-57.9) |
| Trouble thinking clearly | 28.6 (3.7-71.0) | 71.4 (29.0-96.3) | 0.0 (0.0-41.0) | 0.0 (0.0-41.0) | 71.4 (29.0-96.3) | 0.0 (0.0-41.0) |
| Bruised easily | 85.7 (42.1-99.6) | 14.3 (0.4-57.9) | 0.0 (0.0-41.0) | 0.0 (0.0-41.0) | 14.3 (0.4-57.9) | 0.0 (0.0-41.0) |
| Skin problems (scale) | 0.0 (0.0-41.0) | Na | Na | Na | 100.0 (59.0-100.0) | Na |
| Skin colour change | 57.1 (18.4-90.1) | 42.9 (9.9-81.6) | 0.0 (0.0-41.0) | 0.0 (0.0-41.0) | 42.9 (9.9-81.6) | 0.0 (0.0-41.0) |
| Skin itched | 14.3 (0.4-57.9) | 57.1 (18.4-90.1) | 28.6 (3.7-71.0) | 0.0 (0.0-41.0) | 85.7 (42.1-99.6) | 28.6 (3.7-71.0) |
| Skin rash | 57.1 (18.4-90.1) | 14.3 (0.4-57.9) | 28.6 (3.7-71.0) | 0.0 (0.0-41.0) | 42.9 (9.9-81.6) | 28.6 (3.7-71.0) |
| Skin dry/flaky/cracked | 0.0 (0.0-41.0) | 71.4 (29.0-96.3) | 28.6 (3.7-71.0) | 0.0 (0.0-41.0) | 100.0 (59.0-100.0) | 28.6 (3.7-71.0) |
| Skin sore/painful | 42.9 (9.9-81.6) | 42.9 (9.9-81.6) | 14.3 (0.4-57.9) | 0.0 (0.0-41.0) | 57.1 (18.4-90.1) | 14.3 (0.4-57.9) |
| Wounds healing problems | 83.3 (35.9-99.6) | 16.7 (0.4-64.1) | 0.0 (0.0-45.9) | 0.0 (0.0-45.9) | 16.7 (0.4-64.1) | 0.0 (0.0-45.9) |
| Hair loss | 71.4 (29.0-96.3) | 28.6 (3.7-71.0) | 0.0 (0.0-41.0) | 0.0 (0.0-41.0) | 28.6 (3.7-71.0) | 0.0 (0.0-41.0) |
| Nail problems | 67.9 (56.4-78.1) | 20.5 (12.2-31.2) | 9.0 (3.7-17.6) | 2.6 (0.3-9.0) | 32.1 (21.9-43.6) | 11.5 (5.4-20.8) |
| Eye problems (scale) | 14.3 (0.4-57.9) | Na | Na | Na | 85.7 (42.1-99.6) | Na |
| Watery eyes | 57.1 (18.4-90.1) | 28.6 (3.7-71.0) | 14.3 (0.4-57.9) | 0.0 (0.0-41.0) | 42.9 (9.9-81.6) | 14.3 (0.4-57.9) |
| Burning eyes | 28.6 (3.7-71.0) | 71.4 (29.0-96.3) | 0.0 (0.0-41.0) | 0.0 (0.0-41.0) | 71.4 (29.0-96.3) | 0.0 (0.0-41.0) |
| Light sensitive eyes | 85.7 (42.1-99.6) | 14.3 (0.4-57.9) | 0.0 (0.0-41.0) | 0.0 (0.0-41.0) | 14.3 (0.4-57.9) | 0.0 (0.0-41.0) |
| Blurry eyes | 28.6 (3.7-71.0) | 57.1 (18.4-90.1) | 14.3 (0.4-57.9) | 0.0 (0.0-41.0) | 71.4 (29.0-96.3) | 14.3 (0.4-57.9) |
| Red eyes | 85.7 (42.1-99.6) | 0.0 (0.0-41.0) | 14.3 (0.4-57.9) | 0.0 (0.0-41.0) | 14.3 (0.4-57.9) | 14.3 (0.4-57.9) |
| Swelling face/eyes | 85.7 (42.1-99.6) | 14.3 (0.4-57.9) | 0.0 (0.0-41.0) | 0.0 (0.0-41.0) | 14.3 (0.4-57.9) | 0.0 (0.0-41.0) |
| Swelling body parts | 83.3 (35.9-99.6) | 16.7 (0.4-64.1) | 0.0 (0.0-45.9) | 0.0 (0.0-45.9) | 16.7 (0.4-64.1) | 0.0 (0.0-45.9) |
| Hearing problems | 100.0 (59.0-100.0) | 0.0 (0.0-41.0) | 0.0 (0.0-41.0) | 0.0 (0.0-41.0) | 0.0 (0.0-41.0) | 0.0 (0.0-41.0) |
| Nose bleeds | 100.0 (59.0-100.0) | 0.0 (0.0-41.0) | 0.0 (0.0-41.0) | 0.0 (0.0-41.0) | 0.0 (0.0-41.0) | 0.0 (0.0-41.0) |
| Nose problems | 0.0 (0.0-97.5) | 100.0 (2.5-100.0) | 0.0 (0.0-97.5) | 0.0 (0.0-97.5) | 100.0 (2.5-100.0) | 0.0 (0.0-97.5) |
| Dry mouth | 14.3 (0.4-57.9) | 71.4 (29.0-96.3) | 14.3 (0.4-57.9) | 0.0 (0.0-41.0) | 85.7 (42.1-99.6) | 14.3 (0.4-57.9) |
| Pain/soreness mouth | 85.7 (42.1-99.6) | 14.3 (0.4-57.9) | 0.0 (0.0-41.0) | 0.0 (0.0-41.0) | 14.3 (0.4-57.9) | 0.0 (0.0-41.0) |
| Teeth problems | 42.9 (9.9-81.6) | 57.1 (18.4-90.1) | 0.0 (0.0-41.0) | 0.0 (0.0-41.0) | 57.1 (18.4-90.1) | 0.0 (0.0-41.0) |
| Bleeding gums | 57.1 (18.4-90.1) | 42.9 (9.9-81.6) | 0.0 (0.0-41.0) | 0.0 (0.0-41.0) | 42.9 (9.9-81.6) | 0.0 (0.0-41.0) |
| Taste change | 83.3 (35.9-99.6) | 0.0 (0.0-45.9) | 16.7 (0.4-64.1) | 0.0 (0.0-45.9) | 16.7 (0.4-64.1) | 16.7 (0.4-64.1) |
| Appetite loss | 66.7 (22.3-95.7) | 33.3 (4.3-77.7) | 0.0 (0.0-45.9) | 0.0 (0.0-45.9) | 33.3 (4.3-77.7) | 0.0 (0.0-45.9) |
| Mood problems (scale) | 28.6 (3.7-71.0) | Na | Na | Na | 71.4 (29.0-96.3) | Na |
| Depressed | 66.7 (22.3-95.7) | 33.3 (4.3-77.7) | 0.0 (0.0-45.9) | 0.0 (0.0-45.9) | 33.3 (4.3-77.7) | 0.0 (0.0-45.9) |
| Irritable | 50.0 (11.8-88.2) | 50.0 (11.8-88.2) | 0.0 (0.0-45.9) | 0.0 (0.0-45.9) | 50.0 (11.8-88.2) | 0.0 (0.0-45.9) |
| Tense | 16.7 (0.4-64.1) | 83.3 (35.9-99.6) | 0.0 (0.0-45.9) | 0.0 (0.0-45.9) | 83.3 (35.9-99.6) | 0.0 (0.0-45.9) |
| Worried | 16.7 (0.4-64.1) | 83.3 (35.9-99.6) | 0.0 (0.0-45.9) | 0.0 (0.0-45.9) | 83.3 (35.9-99.6) | 0.0 (0.0-45.9) |
| Afraid | 66.7 (22.3-95.7) | 33.3 (4.3-77.7) | 0.0 (0.0-45.9) | 0.0 (0.0-45.9) | 33.3 (4.3-77.7) | 0.0 (0.0-45.9) |
| Insomnia | 42.9 (9.9-81.6) | 57.1 (18.4-90.1) | 0.0 (0.0-41.0) | 0.0 (0.0-41.0) | 57.1 (18.4-90.1) | 0.0 (0.0-41.0) |
| Palpitations | 50.0 (11.8-88.2) | 50.0 (11.8-88.2) | 0.0 (0.0-45.9) | 0.0 (0.0-45.9) | 50.0 (11.8-88.2) | 0.0 (0.0-45.9) |
| Pain in chest | 83.3 (35.9-99.6) | 16.7 (0.4-64.1) | 0.0 (0.0-45.9) | 0.0 (0.0-45.9) | 16.7 (0.4-64.1) | 0.0 (0.0-45.9) |
| Short of breath | 83.3 (35.9-99.6) | 16.7 (0.4-64.1) | 0.0 (0.0-45.9) | 0.0 (0.0-45.9) | 16.7 (0.4-64.1) | 0.0 (0.0-45.9) |
| Coughed | 100.0 (54.1-100.0) | 0.0 (0.0-45.9) | 0.0 (0.0-45.9) | 0.0 (0.0-45.9) | 0.0 (0.0-45.9) | 0.0 (0.0-45.9) |
| Feeling bloated | 33.3 (4.3-77.7) | 33.3 (4.3-77.7) | 33.3 (4.3-77.7) | 0.0 (0.0-45.9) | 66.7 (22.3-95.7) | 33.3 (4.3-77.7) |
| Indigestion | 16.7 (0.4-64.1) | 66.7 (22.3-95.7) | 16.7 (0.4-64.1) | 0.0 (0.0-45.9) | 83.3 (35.9-99.6) | 16.7 (0.4-64.1) |
| Nausea | 66.7 (22.3-95.7) | 16.7 (0.4-64.1) | 16.7 (0.4-64.1) | 0.0 (0.0-45.9) | 33.3 (4.3-77.7) | 16.7 (0.4-64.1) |
| Diarrhoea | 83.3 (35.9-99.6) | 16.7 (0.4-64.1) | 0.0 (0.0-45.9) | 0.0 (0.0-45.9) | 16.7 (0.4-64.1) | 0.0 (0.0-45.9) |
| Abdominal pain/cramps | 50.0 (11.8-88.2) | 50.0 (11.8-88.2) | 0.0 (0.0-45.9) | 0.0 (0.0-45.9) | 50.0 (11.8-88.2) | 0.0 (0.0-45.9) |
| Vomited | 100.0 (47.8-100.0) | 0.0 (0.0-52.2) | 0.0 (0.0-52.2) | 0.0 (0.0-52.2) | 0.0 (0.0-52.2) | 0.0 (0.0-52.2) |
| Obstipation | 50.0 (11.8-88.2) | 50.0 (11.8-88.2) | 0.0 (0.0-45.9) | 0.0 (0.0-45.9) | 50.0 (11.8-88.2) | 0.0 (0.0-45.9) |
| Flatulence | 50.0 (11.8-88.2) | 33.3 (4.3-77.7) | 16.7 (0.4-64.1) | 0.0 (0.0-45.9) | 50.0 (11.8-88.2) | 16.7 (0.4-64.1) |
| Muscle/joint cramps/pain (scale) | 0.0 (0.0-45.9) | Na | Na | Na | 100.0 (54.1-100.0) | Na |
| Frequent urination | 16.7 (0.4-64.1) | 50.0 (11.8-88.2) | 33.3 (4.3-77.7) | 0.0 (0.0-45.9) | 83.3 (35.9-99.6) | 33.3 (4.3-77.7) |
| Muscle cramps | 50.0 (11.8-88.2) | 33.3 (4.3-77.7) | 16.7 (0.4-64.1) | 0.0 (0.0-45.9) | 50.0 (11.8-88.2) | 16.7 (0.4-64.1) |
| Aches/pains muscles/joints | 0.0 (0.0-45.9) | 66.7 (22.3-95.7) | 33.3 (4.3-77.7) | 0.0 (0.0-45.9) | 100.0 (54.1-100.0) | 33.3 (4.3-77.7) |
| Muscle weakness | 83.3 (35.9-99.6) | 16.7 (0.4-64.1) | 0.0 (0.0-45.9) | 0.0 (0.0-45.9) | 16.7 (0.4-64.1) | 0.0 (0.0-45.9) |
| Pale/cold fingers/toes | 0.0 (0.0-97.5) | 100.0 (2.5-100.0) | 0.0 (0.0-97.5) | 0.0 (0.0-97.5) | 100.0 (2.5-100.0) | 0.0 (0.0-97.5) |
| Tingling/numbness hands/feet | 16.7 (0.4-64.1) | 66.7 (22.3-95.7) | 16.7 (0.4-64.1) | 0.0 (0.0-45.9) | 83.3 (35.9-99.6) | 16.7 (0.4-64.1) |
| Problems finger dexterity | 66.7 (22.3-95.7) | 33.3 (4.3-77.7) | 0.0 (0.0-45.9) | 0.0 (0.0-45.9) | 33.3 (4.3-77.7) | 0.0 (0.0-45.9) |
| Trouble walking pain | 66.7 (22.3-95.7) | 33.3 (4.3-77.7) | 0.0 (0.0-45.9) | 0.0 (0.0-45.9) | 33.3 (4.3-77.7) | 0.0 (0.0-45.9) |
| Back pain | 100.0 (2.5-100.0) | 0.0 (0.0-97.5) | 0.0 (0.0-97.5) | 0.0 (0.0-97.5) | 0.0 (0.0-97.5) | 0.0 (0.0-97.5) |
| Pain | 33.3 (4.3-77.7) | 66.7 (22.3-95.7) | 0.0 (0.0-45.9) | 0.0 (0.0-45.9) | 66.7 (22.3-95.7) | 0.0 (0.0-45.9) |
| Fever/chills | 66.7 (22.3-95.7) | 33.3 (4.3-77.7) | 0.0 (0.0-45.9) | 0.0 (0.0-45.9) | 33.3 (4.3-77.7) | 0.0 (0.0-45.9) |
| Sweating excessively | 66.7 (22.3-95.7) | 33.3 (4.3-77.7) | 0.0 (0.0-45.9) | 0.0 (0.0-45.9) | 33.3 (4.3-77.7) | 0.0 (0.0-45.9) |
| Problems tolerating heat/cold | 66.7 (22.3-95.7) | 33.3 (4.3-77.7) | 0.0 (0.0-45.9) | 0.0 (0.0-45.9) | 33.3 (4.3-77.7) | 0.0 (0.0-45.9) |
| Hot flushes | 66.7 (22.3-95.7) | 16.7 (0.4-64.1) | 16.7 (0.4-64.1) | 0.0 (0.0-45.9) | 33.3 (4.3-77.7) | 16.7 (0.4-64.1) |
| Airway infections | 100.0 (2.5-100.0) | 0.0 (0.0-97.5) | 0.0 (0.0-97.5) | 0.0 (0.0-97.5) | 0.0 (0.0-97.5) | 0.0 (0.0-97.5) |
| Other infections | 100.0 (2.5-100.0) | 0.0 (0.0-97.5) | 0.0 (0.0-97.5) | 0.0 (0.0-97.5) | 0.0 (0.0-97.5) | 0.0 (0.0-97.5) |
| Sore/enlarged nipples/breasts | 100.0 (54.1-100.0) | 0.0 (0.0-45.9) | 0.0 (0.0-45.9) | 0.0 (0.0-45.9) | 0.0 (0.0-45.9) | 0.0 (0.0-45.9) |
| Sexuality problems (scale) | 0.0 (0.0-45.9) | Na | Na | Na | 100.0 (54.1-100.0) | Na |
| Less interest sex | 0.0 (0.0-45.9) | 66.7 (22.3-95.7) | 33.3 (4.3-77.7) | 0.0 (0.0-45.9) | 100.0 (54.1-100.0) | 33.3 (4.3-77.7) |
| Less pleasure sex | 33.3 (4.3-77.7) | 33.3 (4.3-77.7) | 33.3 (4.3-77.7) | 0.0 (0.0-45.9) | 66.7 (22.3-95.7) | 33.3 (4.3-77.7) |
| Pain during/after sex | 83.3 (35.9-99.6) | 0.0 (0.0-45.9) | 16.7 (0.4-64.1) | 0.0 (0.0-45.9) | 16.7 (0.4-64.1) | 16.7 (0.4-64.1) |
| Difficult erection | 66.7 (9.4-99.2) | 33.3 (0.8-90.6) | 0.0 (0.0-70.8) | 0.0 (0.0-70.8) | 33.3 (0.8-90.6) | 0.0 (0.0-70.8) |
| Dry vagina | 0.0 (0.0-97.5) | 100.0 (2.5-100.0) | 0.0 (0.0-97.5) | 0.0 (0.0-97.5) | 100.0 (2.5-100.0) | 0.0 (0.0-97.5) |

* At T4 as not available at T1. $ Moderate-severe includes ‘Quite a bit’ and ‘A lot’. Abbreviations: Na: not applicable

Table 24 Prevalence (% with 95%confidence intervals) of symptom scores for seven scales plus 76 single items, in 40 patients in treatment-free remission on T1

| Item/scale | Treatment-free remission | | | | | |
| --- | --- | --- | --- | --- | --- | --- |
|  | Not at all | A little | Quite a bit | A lot | Any severity | Moderate-severe $ |
| Fatigue (scale) | 10.0 (2.8-23.7) | Na | Na | Na | 90.0 (76.3-97.2) | Na |
| Tired | 12.5 (4.2-26.8) | 30.0 (16.6-46.5) | 40.0 (24.9-56.7) | 17.5 (7.3-32.8) | 87.5 (73.2-95.8) | 57.5 (40.9-73.0) |
| Needed rest | 20.0 (9.1-35.6) | 42.5 (27.0-59.1) | 25.0 (12.7-41.2) | 12.5 (4.2-26.8) | 80.0 (64.4-90.9) | 37.5 (22.7-54.2) |
| Lacked energy | 15.4 (5.9-30.5) | 30.8 (17.0-47.6) | 38.5 (23.4-55.4) | 15.4 (5.9-30.5) | 84.6 (69.5-94.1) | 53.8 (37.2-69.9) |
| Felt lethargic | 34.2 (19.6-51.4) | 42.1 (26.3-59.2) | 18.4 (7.7-34.3) | 5.3 (0.6-17.7) | 65.8 (48.6-80.4) | 23.7 (11.4-40.2) |
| Felt weak | 47.2 (30.4-64.5) | 38.9 (23.1-56.5) | 11.1 (3.1-26.1) | 2.8 (0.1-14.5) | 52.8 (35.5-69.6) | 13.9 (4.7-29.5) |
| Sleepy | 25.0 (12.1-42.2) | 52.8 (35.5-69.6) | 11.1 (3.1-26.1) | 11.1 (3.1-26.1) | 75.0 (57.8-87.9) | 22.2 (10.1-39.2) |
| Headaches | 72.2 (54.8-85.8) | 13.9 (4.7-29.5) | 11.1 (3.1-26.1) | 2.8 (0.1-14.5) | 27.8 (14.2-45.2) | 13.9 (4.7-29.5) |
| Dizziness | 71.4 (53.7-85.4) | 22.9 (10.4-40.1) | 2.9 (0.1-14.9) | 2.9 (0.1-14.9) | 28.6 (14.6-46.3) | 5.7 (0.7-19.2) |
| Cognitive problems (scale) | 30.6 (16.3-48.1) | Na | Na | Na | 69.4 (51.9-83.7) | Na |
| Difficulty remembering | 45.7 (28.8-63.4) | 42.9 (26.3-60.6) | 8.6 (1.8-23.1) | 2.9 (0.1-14.9) | 54.3 (36.6-71.2) | 11.4 (3.2-26.7) |
| Difficulty concentrating | 45.7 (28.8-63.4) | 37.1 (21.5-55.1) | 11.4 (3.2-26.7) | 5.7 (0.7-19.2) | 54.3 (36.6-71.2) | 17.1 (6.6-33.6) |
| Trouble thinking clearly | 52.8 (35.5-69.6) | 38.9 (23.1-56.5) | 5.6 (0.7-18.7) | 2.8 (0.1-14.5) | 47.2 (30.4-64.5) | 8.3 (1.8-22.5) |
| Bruised easily | 83.3 (67.2-93.6) | 13.9 (4.7-29.5) | 2.8 (0.1-14.5) | 0.0 (0.0-9.7) | 16.7 (6.4-32.8) | 2.8 (0.1-14.5) |
| Skin problems (scale) | 17.1 (6.6-33.6) | Na | Na | Na | 82.9 (66.4-93.4) | Na |
| Skin colour change | 74.3 (56.7-87.5) | 22.9 (10.4-40.1) | 2.9 (0.1-14.9) | 0.0 (0.0-10.0) | 25.7 (12.5-43.3) | 2.9 (0.1-14.9) |
| Skin itched | 47.1 (29.8-64.9) | 29.4 (15.1-47.5) | 20.6 (8.7-37.9) | 2.9 (0.1-15.3) | 52.9 (35.1-70.2) | 23.5 (10.7-41.2) |
| Skin rash | 60.0 (42.1-76.1) | 25.7 (12.5-43.3) | 8.6 (1.8-23.1) | 5.7 (0.7-19.2) | 40.0 (23.9-57.9) | 14.3 (4.8-30.3) |
| Skin dry/flaky/cracked | 37.1 (21.5-55.1) | 31.4 (16.9-49.3) | 31.4 (16.9-49.3) | 0.0 (0.0-10.0) | 62.9 (44.9-78.5) | 31.4 (16.9-49.3) |
| Skin sore/painful | 68.6 (50.7-83.1) | 20.0 (8.4-36.9) | 8.6 (1.8-23.1) | 2.9 (0.1-14.9) | 31.4 (16.9-49.3) | 11.4 (3.2-26.7) |
| Wounds healing problems | 86.1 (70.5-95.3) | 8.3 (1.8-22.5) | 2.8 (0.1-14.5) | 2.8 (0.1-14.5) | 13.9 (4.7-29.5) | 5.6 (0.7-18.7) |
| Hair loss | 69.4 (51.9-83.7) | 19.4 (8.2-36.0) | 11.1 (3.1-26.1) | 0.0 (0.0-9.7) | 30.6 (16.3-48.1) | 11.1 (3.1-26.1) |
| Nail problems | 67.9 (56.4-78.1) | 20.5 (12.2-31.2) | 9.0 (3.7-17.6) | 2.6 (0.3-9.0) | 32.1 (21.9-43.6) | 11.5 (5.4-20.8) |
| Eye problems (scale) | 34.3 (19.1-52.2) | Na | Na | Na | 65.7 (47.8-80.9) | Na |
| Watery eyes | 68.6 (50.7-83.1) | 17.1 (6.6-33.6) | 14.3 (4.8-30.3) | 0.0 (0.0-10.0) | 31.4 (16.9-49.3) | 14.3 (4.8-30.3) |
| Burning eyes | 58.8 (40.7-75.4) | 29.4 (15.1-47.5) | 5.9 (0.7-19.7) | 5.9 (0.7-19.7) | 41.2 (24.6-59.3) | 11.8 (3.3-27.5) |
| Light sensitive eyes | 57.1 (39.4-73.7) | 14.3 (4.8-30.3) | 22.9 (10.4-40.1) | 5.7 (0.7-19.2) | 42.9 (26.3-60.6) | 28.6 (14.6-46.3) |
| Blurry eyes | 61.8 (43.6-77.8) | 29.4 (15.1-47.5) | 2.9 (0.1-15.3) | 5.9 (0.7-19.7) | 38.2 (22.2-56.4) | 8.8 (1.9-23.7) |
| Red eyes | 91.2 (76.3-98.1) | 5.9 (0.7-19.7) | 2.9 (0.1-15.3) | 0.0 (0.0-10.3) | 8.8 (1.9-23.7) | 2.9 (0.1-15.3) |
| Swelling face/eyes | 79.4 (62.1-91.3) | 8.8 (1.9-23.7) | 5.9 (0.7-19.7) | 5.9 (0.7-19.7) | 20.6 (8.7-37.9) | 11.8 (3.3-27.5) |
| Swelling body parts | 48.4 (30.2-66.9) | 35.5 (19.2-54.6) | 12.9 (3.6-29.8) | 3.2 (0.1-16.7) | 51.6 (33.1-69.8) | 16.1 (5.5-33.7) |
| Hearing problems | 73.5 (55.6-87.1) | 8.8 (1.9-23.7) | 8.8 (1.9-23.7) | 8.8 (1.9-23.7) | 26.5 (12.9-44.4) | 17.6 (6.8-34.5) |
| Nose bleeds | 88.2 (72.5-96.7) | 8.8 (1.9-23.7) | 2.9 (0.1-15.3) | 0.0 (0.0-10.3) | 11.8 (3.3-27.5) | 2.9 (0.1-15.3) |
| Nose problems | 25.0 (3.2-65.1) | 62.5 (24.5-91.5) | 12.5 (0.3-52.7) | 0.0 (0.0-36.9) | 75.0 (34.9-96.8) | 12.5 (0.3-52.7) |
| Dry mouth | 61.8 (43.6-77.8) | 23.5 (10.7-41.2) | 14.7 (5.0-31.1) | 0.0 (0.0-10.3) | 38.2 (22.2-56.4) | 14.7 (5.0-31.1) |
| Pain/soreness mouth | 73.5 (55.6-87.1) | 23.5 (10.7-41.2) | 2.9 (0.1-15.3) | 0.0 (0.0-10.3) | 26.5 (12.9-44.4) | 2.9 (0.1-15.3) |
| Teeth problems | 73.5 (55.6-87.1) | 20.6 (8.7-37.9) | 5.9 (0.7-19.7) | 0.0 (0.0-10.3) | 26.5 (12.9-44.4) | 5.9 (0.7-19.7) |
| Bleeding gums | 84.8 (68.1-94.9) | 9.1 (1.9-24.3) | 6.1 (0.7-20.2) | 0.0 (0.0-10.6) | 15.2 (5.1-31.9) | 6.1 (0.7-20.2) |
| Taste change | 91.2 (76.3-98.1) | 5.9 (0.7-19.7) | 2.9 (0.1-15.3) | 0.0 (0.0-10.3) | 8.8 (1.9-23.7) | 2.9 (0.1-15.3) |
| Appetite loss | 90.9 (75.7-98.1) | 6.1 (0.7-20.2) | 3.0 (0.1-15.8) | 0.0 (0.0-10.6) | 9.1 (1.9-24.3) | 3.0 (0.1-15.8) |
| Mood problems (scale) | 16.7 (6.4-32.8) | Na | Na | Na | 83.3 (67.2-93.6) | Na |
| Depressed | 51.5 (33.5-69.2) | 45.5 (28.1-63.6) | 3.0 (0.1-15.8) | 0.0 (0.0-10.6) | 48.5 (30.8-66.5) | 3.0 (0.1-15.8) |
| Irritable | 45.5 (28.1-63.6) | 45.5 (28.1-63.6) | 9.1 (1.9-24.3) | 0.0 (0.0-10.6) | 54.5 (36.4-71.9) | 9.1 (1.9-24.3) |
| Tense | 50.0 (31.9-68.1) | 43.8 (26.4-62.3) | 6.2 (0.8-20.8) | 0.0 (0.0-10.9) | 50.0 (31.9-68.1) | 6.2 (0.8-20.8) |
| Worried | 30.3 (15.6-48.7) | 60.6 (42.1-77.1) | 9.1 (1.9-24.3) | 0.0 (0.0-10.6) | 69.7 (51.3-84.4) | 9.1 (1.9-24.3) |
| Afraid | 75.8 (57.7-88.9) | 21.2 (9.0-38.9) | 3.0 (0.1-15.8) | 0.0 (0.0-10.6) | 24.2 (11.1-42.3) | 3.0 (0.1-15.8) |
| Insomnia | 51.4 (34.0-68.6) | 28.6 (14.6-46.3) | 11.4 (3.2-26.7) | 8.6 (1.8-23.1) | 48.6 (31.4-66.0) | 20.0 (8.4-36.9) |
| Palpitations | 57.6 (39.2-74.5) | 36.4 (20.4-54.9) | 6.1 (0.7-20.2) | 0.0 (0.0-10.6) | 42.4 (25.5-60.8) | 6.1 (0.7-20.2) |
| Pain in chest | 87.5 (71.0-96.5) | 12.5 (3.5-29.0) | 0.0 (0.0-10.9) | 0.0 (0.0-10.9) | 12.5 (3.5-29.0) | 0.0 (0.0-10.9) |
| Short of breath | 68.8 (50.0-83.9) | 15.6 (5.3-32.8) | 12.5 (3.5-29.0) | 3.1 (0.1-16.2) | 31.2 (16.1-50.0) | 15.6 (5.3-32.8) |
| Coughed | 71.9 (53.3-86.3) | 21.9 (9.3-40.0) | 3.1 (0.1-16.2) | 3.1 (0.1-16.2) | 28.1 (13.7-46.7) | 6.2 (0.8-20.8) |
| Feeling bloated | 68.8 (50.0-83.9) | 15.6 (5.3-32.8) | 9.4 (2.0-25.0) | 6.2 (0.8-20.8) | 31.2 (16.1-50.0) | 15.6 (5.3-32.8) |
| Indigestion | 68.8 (50.0-83.9) | 15.6 (5.3-32.8) | 12.5 (3.5-29.0) | 3.1 (0.1-16.2) | 31.2 (16.1-50.0) | 15.6 (5.3-32.8) |
| Nausea | 87.1 (70.2-96.4) | 12.9 (3.6-29.8) | 0.0 (0.0-11.2) | 0.0 (0.0-11.2) | 12.9 (3.6-29.8) | 0.0 (0.0-11.2) |
| Diarrhoea | 75.0 (56.6-88.5) | 18.8 (7.2-36.4) | 3.1 (0.1-16.2) | 3.1 (0.1-16.2) | 25.0 (11.5-43.4) | 6.2 (0.8-20.8) |
| Abdominal pain/cramps | 68.8 (50.0-83.9) | 28.1 (13.7-46.7) | 3.1 (0.1-16.2) | 0.0 (0.0-10.9) | 31.2 (16.1-50.0) | 3.1 (0.1-16.2) |
| Vomited | 100.0 (89.1-100.0) | 0.0 (0.0-10.9) | 0.0 (0.0-10.9) | 0.0 (0.0-10.9) | 0.0 (0.0-10.9) | 0.0 (0.0-10.9) |
| Obstipation | 74.2 (55.4-88.1) | 22.6 (9.6-41.1) | 0.0 (0.0-11.2) | 3.2 (0.1-16.7) | 25.8 (11.9-44.6) | 3.2 (0.1-16.7) |
| Flatulence | 56.2 (37.7-73.6) | 25.0 (11.5-43.4) | 15.6 (5.3-32.8) | 3.1 (0.1-16.2) | 43.8 (26.4-62.3) | 18.8 (7.2-36.4) |
| Muscle/joint cramps/pain (scale) | 6.2 (0.8-20.8) | Na | Na | Na | 93.8 (79.2-99.2) | Na |
| Frequent urination | 50.0 (31.9-68.1) | 31.2 (16.1-50.0) | 15.6 (5.3-32.8) | 3.1 (0.1-16.2) | 50.0 (31.9-68.1) | 18.8 (7.2-36.4) |
| Muscle cramps | 25.0 (11.5-43.4) | 50.0 (31.9-68.1) | 18.8 (7.2-36.4) | 6.2 (0.8-20.8) | 75.0 (56.6-88.5) | 25.0 (11.5-43.4) |
| Aches/pains muscles/joints | 6.2 (0.8-20.8) | 40.6 (23.7-59.4) | 40.6 (23.7-59.4) | 12.5 (3.5-29.0) | 93.8 (79.2-99.2) | 53.1 (34.7-70.9) |
| Muscle weakness | 46.9 (29.1-65.3) | 43.8 (26.4-62.3) | 9.4 (2.0-25.0) | 0.0 (0.0-10.9) | 53.1 (34.7-70.9) | 9.4 (2.0-25.0) |
| Pale/cold fingers/toes | 37.5 (8.5-75.5) | 62.5 (24.5-91.5) | 0.0 (0.0-36.9) | 0.0 (0.0-36.9) | 62.5 (24.5-91.5) | 0.0 (0.0-36.9) |
| Tingling/numbness hands/feet | 68.8 (50.0-83.9) | 25.0 (11.5-43.4) | 6.2 (0.8-20.8) | 0.0 (0.0-10.9) | 31.2 (16.1-50.0) | 6.2 (0.8-20.8) |
| Problems finger dexterity | 61.3 (42.2-78.2) | 29.0 (14.2-48.0) | 6.5 (0.8-21.4) | 3.2 (0.1-16.7) | 38.7 (21.8-57.8) | 9.7 (2.0-25.8) |
| Trouble walking pain | 50.0 (31.9-68.1) | 31.2 (16.1-50.0) | 18.8 (7.2-36.4) | 0.0 (0.0-10.9) | 50.0 (31.9-68.1) | 18.8 (7.2-36.4) |
| Back pain | 37.5 (8.5-75.5) | 25.0 (3.2-65.1) | 25.0 (3.2-65.1) | 12.5 (0.3-52.7) | 62.5 (24.5-91.5) | 37.5 (8.5-75.5) |
| Pain | 48.4 (30.2-66.9) | 35.5 (19.2-54.6) | 16.1 (5.5-33.7) | 0.0 (0.0-11.2) | 51.6 (33.1-69.8) | 16.1 (5.5-33.7) |
| Fever/chills | 93.5 (78.6-99.2) | 6.5 (0.8-21.4) | 0.0 (0.0-11.2) | 0.0 (0.0-11.2) | 6.5 (0.8-21.4) | 0.0 (0.0-11.2) |
| Sweating excessively | 58.1 (39.1-75.5) | 29.0 (14.2-48.0) | 12.9 (3.6-29.8) | 0.0 (0.0-11.2) | 41.9 (24.5-60.9) | 12.9 (3.6-29.8) |
| Problems tolerating heat/cold | 61.3 (42.2-78.2) | 29.0 (14.2-48.0) | 9.7 (2.0-25.8) | 0.0 (0.0-11.2) | 38.7 (21.8-57.8) | 9.7 (2.0-25.8) |
| Hot flushes | 71.0 (52.0-85.8) | 25.8 (11.9-44.6) | 3.2 (0.1-16.7) | 0.0 (0.0-11.2) | 29.0 (14.2-48.0) | 3.2 (0.1-16.7) |
| Airway infections | 100.0 (59.0-100.0) | 0.0 (0.0-41.0) | 0.0 (0.0-41.0) | 0.0 (0.0-41.0) | 0.0 (0.0-41.0) | 0.0 (0.0-41.0) |
| Other infections | 87.5 (47.3-99.7) | 12.5 (0.3-52.7) | 0.0 (0.0-36.9) | 0.0 (0.0-36.9) | 12.5 (0.3-52.7) | 0.0 (0.0-36.9) |
| Sore/enlarged nipples/breasts | 90.3 (74.2-98.0) | 9.7 (2.0-25.8) | 0.0 (0.0-11.2) | 0.0 (0.0-11.2) | 9.7 (2.0-25.8) | 0.0 (0.0-11.2) |
| Sexuality problems (scale) | 46.7 (28.3-65.7) | Na | Na | Na | 53.3 (34.3-71.7) | Na |
| Less interest sex | 53.3 (34.3-71.7) | 20.0 (7.7-38.6) | 10.0 (2.1-26.5) | 16.7 (5.6-34.7) | 46.7 (28.3-65.7) | 26.7 (12.3-45.9) |
| Less pleasure sex | 60.0 (40.6-77.3) | 13.3 (3.8-30.7) | 16.7 (5.6-34.7) | 10.0 (2.1-26.5) | 40.0 (22.7-59.4) | 26.7 (12.3-45.9) |
| Pain during/after sex | 73.3 (54.1-87.7) | 20.0 (7.7-38.6) | 0.0 (0.0-11.6) | 6.7 (0.8-22.1) | 26.7 (12.3-45.9) | 6.7 (0.8-22.1) |
| Difficult erection | 71.4 (41.9-91.6) | 21.4 (4.7-50.8) | 7.1 (0.2-33.9) | 0.0 (0.0-23.2) | 28.6 (8.4-58.1) | 7.1 (0.2-33.9) |
| Dry vagina | 40.0 (16.3-67.7) | 26.7 (7.8-55.1) | 20.0 (4.3-48.1) | 13.3 (1.7-40.5) | 60.0 (32.3-83.7) | 33.3 (11.8-61.6) |

* At T4 as not available at T1. $ Moderate-severe includes ‘Quite a bit’ and ‘A lot’. Abbreviations: Na: not applicable

Table 25 Prevalence (% with 95%confidence intervals) of symptom scores for seven scales plus 76 single items, in 227 patients on any TKI on T1

| Item/scale | Any TKI | | | | | |
| --- | --- | --- | --- | --- | --- | --- |
|  | Not at all | A little | Quite a bit | A lot | Any severity | Moderate-severe $ |
| Fatigue (scale) | 8.4 (5.1-12.8) | Na | Na | Na | 91.6 (87.2-94.9) | Na |
| Tired | 12.3 (8.4-17.3) | 36.1 (29.9-42.7) | 33.9 (27.8-40.5) | 17.6 (12.9-23.2) | 87.7 (82.7-91.6) | 51.5 (44.8-58.2) |
| Needed rest | 17.6 (12.9-23.2) | 40.1 (33.7-46.8) | 34.8 (28.6-41.4) | 7.5 (4.4-11.7) | 82.4 (76.8-87.1) | 42.3 (35.8-49.0) |
| Lacked energy | 17.3 (12.5-22.9) | 33.6 (27.4-40.3) | 35.5 (29.1-42.2) | 13.6 (9.4-18.9) | 82.7 (77.1-87.5) | 49.1 (42.3-55.9) |
| Felt lethargic | 29.2 (23.1-35.9) | 41.1 (34.4-48.1) | 23.9 (18.3-30.3) | 5.7 (3.0-9.8) | 70.8 (64.1-76.9) | 29.7 (23.6-36.4) |
| Felt weak | 35.0 (28.5-41.9) | 39.8 (33.1-46.8) | 20.4 (15.1-26.5) | 4.9 (2.4-8.7) | 65.0 (58.1-71.5) | 25.2 (19.5-31.7) |
| Sleepy | 24.0 (18.3-30.5) | 44.6 (37.7-51.7) | 26.0 (20.1-32.6) | 5.4 (2.7-9.4) | 76.0 (69.5-81.7) | 31.4 (25.1-38.2) |
| Headaches | 65.0 (57.9-71.6) | 25.4 (19.5-32.1) | 8.1 (4.7-12.9) | 1.5 (0.3-4.4) | 35.0 (28.4-42.1) | 9.6 (5.9-14.7) |
| Dizziness | 66.2 (59.0-72.8) | 28.2 (22.0-35.1) | 4.6 (2.1-8.6) | 1.0 (0.1-3.7) | 33.8 (27.2-41.0) | 5.6 (2.8-9.9) |
| Cognitive problems (scale) | 28.9 (22.6-35.8) | Na | Na | Na | 71.1 (64.2-77.4) | Na |
| Difficulty remembering | 39.7 (32.8-46.9) | 44.8 (37.7-52.1) | 12.9 (8.5-18.4) | 2.6 (0.8-5.9) | 60.3 (53.1-67.2) | 15.5 (10.7-21.3) |
| Difficulty concentrating | 46.6 (39.4-53.9) | 34.2 (27.5-41.4) | 15.5 (10.7-21.4) | 3.6 (1.5-7.3) | 53.4 (46.1-60.6) | 19.2 (13.9-25.4) |
| Trouble thinking clearly | 47.9 (40.7-55.2) | 40.1 (33.1-47.4) | 9.9 (6.1-15.0) | 2.1 (0.6-5.2) | 52.1 (44.8-59.3) | 12.0 (7.7-17.4) |
| Bruised easily | 61.8 (54.5-68.7) | 26.7 (20.6-33.6) | 8.9 (5.3-13.9) | 2.6 (0.9-6.0) | 38.2 (31.3-45.5) | 11.5 (7.4-16.9) |
| Skin problems (scale) | 16.9 (11.9-23.1) | Na | Na | Na | 83.1 (76.9-88.1) | Na |
| Skin colour change | 63.1 (55.8-70.0) | 25.7 (19.6-32.6) | 9.6 (5.8-14.8) | 1.6 (0.3-4.6) | 36.9 (30.0-44.2) | 11.2 (7.1-16.7) |
| Skin itched | 37.0 (30.1-44.3) | 37.6 (30.6-44.9) | 18.5 (13.3-24.8) | 6.9 (3.7-11.5) | 63.0 (55.7-69.9) | 25.4 (19.4-32.2) |
| Skin rash | 50.3 (42.9-57.6) | 31.6 (25.0-38.7) | 13.9 (9.3-19.7) | 4.3 (1.9-8.3) | 49.7 (42.4-57.1) | 18.2 (12.9-24.5) |
| Skin dry/flaky/cracked | 38.8 (31.8-46.2) | 36.7 (29.8-44.0) | 20.7 (15.2-27.2) | 3.7 (1.5-7.5) | 61.2 (53.8-68.2) | 24.5 (18.5-31.3) |
| Skin sore/painful | 55.6 (48.2-62.8) | 32.3 (25.7-39.4) | 11.1 (7.0-16.5) | 1.1 (0.1-3.8) | 44.4 (37.2-51.8) | 12.2 (7.9-17.7) |
| Wounds healing problems | 78.9 (72.5-84.5) | 16.8 (11.8-22.9) | 2.6 (0.9-6.0) | 1.6 (0.3-4.5) | 21.1 (15.5-27.5) | 4.2 (1.8-8.1) |
| Hair loss | 57.1 (49.8-64.3) | 29.1 (22.7-36.1) | 10.1 (6.2-15.3) | 3.7 (1.5-7.5) | 42.9 (35.7-50.2) | 13.8 (9.2-19.5) |
| Nail problems | 67.9 (56.4-78.1) | 20.5 (12.2-31.2) | 9.0 (3.7-17.6) | 2.6 (0.3-9.0) | 32.1 (21.9-43.6) | 11.5 (5.4-20.8) |
| Eye problems (scale) | 13.4 (8.8-19.1) | Na | Na | Na | 86.6 (80.9-91.2) | Na |
| Watery eyes | 45.9 (38.6-53.4) | 28.1 (21.8-35.2) | 21.6 (15.9-28.3) | 4.3 (1.9-8.3) | 54.1 (46.6-61.4) | 25.9 (19.8-32.9) |
| Burning eyes | 39.7 (32.6-47.1) | 39.7 (32.6-47.1) | 19.0 (13.6-25.4) | 1.6 (0.3-4.7) | 60.3 (52.9-67.4) | 20.7 (15.0-27.2) |
| Light sensitive eyes | 43.7 (36.4-51.2) | 32.8 (26.0-40.1) | 17.5 (12.3-23.8) | 6.0 (3.0-10.5) | 56.3 (48.8-63.6) | 23.5 (17.6-30.3) |
| Blurry eyes | 41.3 (34.1-48.8) | 43.5 (36.2-51.0) | 13.6 (9.0-19.4) | 1.6 (0.3-4.7) | 58.7 (51.2-65.9) | 15.2 (10.4-21.2) |
| Red eyes | 73.8 (66.8-80.0) | 18.0 (12.8-24.4) | 7.1 (3.8-11.8) | 1.1 (0.1-3.9) | 26.2 (20.0-33.2) | 8.2 (4.7-13.2) |
| Swelling face/eyes | 54.8 (47.4-62.0) | 22.3 (16.6-29.0) | 17.0 (11.9-23.2) | 5.9 (3.0-10.2) | 45.2 (38.0-52.6) | 22.9 (17.1-29.5) |
| Swelling body parts | 62.0 (54.5-69.1) | 24.6 (18.5-31.6) | 11.7 (7.4-17.4) | 1.7 (0.3-4.8) | 38.0 (30.9-45.5) | 13.4 (8.8-19.3) |
| Hearing problems | 76.3 (69.6-82.3) | 15.6 (10.7-21.6) | 5.9 (3.0-10.3) | 2.2 (0.6-5.4) | 23.7 (17.7-30.4) | 8.1 (4.6-13.0) |
| Nose bleeds | 87.6 (82.0-92.0) | 10.8 (6.7-16.1) | 0.5 (0.0-3.0) | 1.1 (0.1-3.8) | 12.4 (8.0-18.0) | 1.6 (0.3-4.6) |
| Nose problems | 52.2 (39.8-64.4) | 21.7 (12.7-33.3) | 24.6 (15.1-36.5) | 1.4 (0.0-7.8) | 47.8 (35.6-60.2) | 26.1 (16.3-38.1) |
| Dry mouth | 54.1 (46.6-61.4) | 29.2 (22.8-36.3) | 10.8 (6.7-16.2) | 5.9 (3.0-10.4) | 45.9 (38.6-53.4) | 16.8 (11.7-22.9) |
| Pain/soreness mouth | 77.4 (70.7-83.2) | 16.7 (11.6-22.8) | 4.3 (1.9-8.3) | 1.6 (0.3-4.6) | 22.6 (16.8-29.3) | 5.9 (3.0-10.3) |
| Teeth problems | 75.5 (68.7-81.6) | 17.4 (12.2-23.7) | 6.0 (3.0-10.4) | 1.1 (0.1-3.9) | 24.5 (18.4-31.3) | 7.1 (3.8-11.8) |
| Bleeding gums | 78.8 (72.2-84.5) | 17.9 (12.7-24.3) | 2.7 (0.9-6.2) | 0.5 (0.0-3.0) | 21.2 (15.5-27.8) | 3.3 (1.2-7.0) |
| Taste change | 73.9 (66.9-80.1) | 16.8 (11.7-23.1) | 6.0 (3.0-10.4) | 3.3 (1.2-7.0) | 26.1 (19.9-33.1) | 9.2 (5.5-14.4) |
| Appetite loss | 71.2 (64.1-77.6) | 18.5 (13.1-24.9) | 7.6 (4.2-12.4) | 2.7 (0.9-6.2) | 28.8 (22.4-35.9) | 10.3 (6.3-15.7) |
| Mood problems (scale) | 19.5 (14.2-25.7) | Na | Na | Na | 80.5 (74.3-85.8) | Na |
| Depressed | 59.0 (51.5-66.2) | 25.7 (19.5-32.6) | 10.9 (6.8-16.4) | 4.4 (1.9-8.4) | 41.0 (33.8-48.5) | 15.3 (10.4-21.3) |
| Irritable | 52.5 (45.0-59.9) | 32.2 (25.5-39.5) | 12.0 (7.7-17.6) | 3.3 (1.2-7.0) | 47.5 (40.1-55.0) | 15.3 (10.4-21.3) |
| Tense | 51.6 (44.1-59.1) | 33.5 (26.7-40.9) | 10.4 (6.4-15.8) | 4.4 (1.9-8.5) | 48.4 (40.9-55.9) | 14.8 (10.0-20.8) |
| Worried | 41.5 (34.3-49.0) | 42.1 (34.8-49.6) | 13.1 (8.6-18.9) | 3.3 (1.2-7.0) | 58.5 (51.0-65.7) | 16.4 (11.3-22.6) |
| Afraid | 71.7 (64.5-78.1) | 18.9 (13.5-25.4) | 6.7 (3.5-11.4) | 2.8 (0.9-6.4) | 28.3 (21.9-35.5) | 9.4 (5.6-14.7) |
| Insomnia | 42.7 (35.7-49.9) | 30.2 (23.9-37.0) | 19.1 (13.9-25.3) | 8.0 (4.7-12.7) | 57.3 (50.1-64.3) | 27.1 (21.1-33.9) |
| Palpitations | 56.0 (48.5-63.3) | 28.8 (22.4-35.9) | 12.5 (8.1-18.2) | 2.7 (0.9-6.2) | 44.0 (36.7-51.5) | 15.2 (10.4-21.2) |
| Pain in chest | 79.1 (72.5-84.8) | 17.0 (11.9-23.3) | 2.7 (0.9-6.3) | 1.1 (0.1-3.9) | 20.9 (15.2-27.5) | 3.8 (1.6-7.8) |
| Short of breath | 50.5 (43.1-58.0) | 32.1 (25.4-39.3) | 14.7 (9.9-20.6) | 2.7 (0.9-6.2) | 49.5 (42.0-56.9) | 17.4 (12.2-23.7) |
| Coughed | 67.0 (59.7-73.8) | 21.4 (15.7-28.1) | 9.3 (5.5-14.5) | 2.2 (0.6-5.5) | 33.0 (26.2-40.3) | 11.5 (7.3-17.1) |
| Feeling bloated | 49.5 (42.0-56.9) | 28.3 (21.9-35.4) | 16.8 (11.7-23.1) | 5.4 (2.6-9.8) | 50.5 (43.1-58.0) | 22.3 (16.5-29.0) |
| Indigestion | 59.9 (52.4-67.1) | 22.5 (16.7-29.3) | 12.1 (7.7-17.7) | 5.5 (2.7-9.9) | 40.1 (32.9-47.6) | 17.6 (12.3-23.9) |
| Nausea | 61.1 (53.6-68.3) | 27.2 (20.9-34.3) | 10.0 (6.0-15.3) | 1.7 (0.3-4.8) | 38.9 (31.7-46.4) | 11.7 (7.4-17.3) |
| Diarrhoea | 61.0 (53.5-68.1) | 22.5 (16.7-29.3) | 7.7 (4.3-12.6) | 8.8 (5.1-13.9) | 39.0 (31.9-46.5) | 16.5 (11.4-22.7) |
| Abdominal pain/cramps | 51.7 (44.1-59.2) | 31.1 (24.4-38.4) | 11.7 (7.4-17.3) | 5.6 (2.7-10.0) | 48.3 (40.8-55.9) | 17.2 (12.0-23.5) |
| Vomited | 86.2 (80.3-90.9) | 11.6 (7.3-17.2) | 2.2 (0.6-5.6) | 0.0 (0.0-2.0) | 13.8 (9.1-19.7) | 2.2 (0.6-5.6) |
| Obstipation | 66.3 (58.9-73.1) | 23.2 (17.3-30.0) | 5.0 (2.3-9.2) | 5.5 (2.7-9.9) | 33.7 (26.9-41.1) | 10.5 (6.4-15.9) |
| Flatulence | 33.5 (26.7-40.9) | 33.0 (26.1-40.4) | 24.6 (18.5-31.6) | 8.9 (5.2-14.1) | 66.5 (59.1-73.3) | 33.5 (26.7-40.9) |
| Muscle/joint cramps/pain (scale) | 15.0 (10.1-21.1) | Na | Na | Na | 85.0 (78.9-89.9) | Na |
| Frequent urination | 42.5 (35.1-50.1) | 27.4 (21.0-34.5) | 22.9 (17.0-29.8) | 7.3 (3.9-12.1) | 57.5 (49.9-64.9) | 30.2 (23.5-37.5) |
| Muscle cramps | 35.2 (28.2-42.7) | 37.4 (30.3-45.0) | 17.3 (12.1-23.7) | 10.1 (6.1-15.4) | 64.8 (57.3-71.8) | 27.4 (21.0-34.5) |
| Aches/pains muscles/joints | 25.7 (19.5-32.8) | 30.7 (24.1-38.0) | 30.7 (24.1-38.0) | 12.8 (8.3-18.7) | 74.3 (67.2-80.5) | 43.6 (36.2-51.2) |
| Muscle weakness | 57.3 (49.7-64.7) | 28.7 (22.1-35.9) | 12.4 (7.9-18.1) | 1.7 (0.3-4.8) | 42.7 (35.3-50.3) | 14.0 (9.3-20.0) |
| Pale/cold fingers/toes | 58.0 (45.5-69.8) | 15.9 (8.2-26.7) | 13.0 (6.1-23.3) | 13.0 (6.1-23.3) | 42.0 (30.2-54.5) | 26.1 (16.3-38.1) |
| Tingling/numbness hands/feet | 45.8 (38.4-53.4) | 33.5 (26.7-40.9) | 14.0 (9.2-19.9) | 6.7 (3.5-11.4) | 54.2 (46.6-61.6) | 20.7 (15.0-27.3) |
| Problems finger dexterity | 71.9 (64.7-78.4) | 21.9 (16.1-28.7) | 4.5 (2.0-8.7) | 1.7 (0.3-4.8) | 28.1 (21.6-35.3) | 6.2 (3.1-10.8) |
| Trouble walking pain | 58.7 (51.1-66.0) | 25.7 (19.5-32.8) | 8.9 (5.2-14.1) | 6.7 (3.5-11.4) | 41.3 (34.0-48.9) | 15.6 (10.7-21.8) |
| Back pain | 46.4 (34.3-58.8) | 37.7 (26.3-50.2) | 10.1 (4.2-19.8) | 5.8 (1.6-14.2) | 53.6 (41.2-65.7) | 15.9 (8.2-26.7) |
| Pain | 48.3 (40.8-55.9) | 34.4 (27.5-41.9) | 13.3 (8.7-19.2) | 3.9 (1.6-7.8) | 51.7 (44.1-59.2) | 17.2 (12.0-23.5) |
| Fever/chills | 76.0 (69.0-82.0) | 19.6 (14.0-26.1) | 3.4 (1.2-7.2) | 1.1 (0.1-4.0) | 24.0 (18.0-31.0) | 4.5 (1.9-8.6) |
| Sweating excessively | 62.2 (54.7-69.3) | 23.9 (17.9-30.8) | 12.2 (7.8-17.9) | 1.7 (0.3-4.8) | 37.8 (30.7-45.3) | 13.9 (9.2-19.8) |
| Problems tolerating heat/cold | 55.9 (48.3-63.3) | 31.3 (24.6-38.6) | 8.9 (5.2-14.1) | 3.9 (1.6-7.9) | 44.1 (36.7-51.7) | 12.8 (8.3-18.7) |
| Hot flushes | 73.7 (66.7-80.0) | 17.3 (12.1-23.7) | 5.6 (2.7-10.0) | 3.4 (1.2-7.2) | 26.3 (20.0-33.3) | 8.9 (5.2-14.1) |
| Airway infections | 79.7 (68.3-88.4) | 13.0 (6.1-23.3) | 2.9 (0.4-10.1) | 4.3 (0.9-12.2) | 20.3 (11.6-31.7) | 7.2 (2.4-16.1) |
| Other infections | 88.4 (78.4-94.9) | 5.8 (1.6-14.2) | 2.9 (0.4-10.1) | 2.9 (0.4-10.1) | 11.6 (5.1-21.6) | 5.8 (1.6-14.2) |
| Sore/enlarged nipples/breasts | 90.4 (85.1-94.3) | 9.0 (5.2-14.2) | 0.6 (0.0-3.1) | 0.0 (0.0-2.1) | 9.6 (5.7-14.9) | 0.6 (0.0-3.1) |
| Sexuality problems (scale) | 31.8 (24.9-39.3) | Na | Na | Na | 68.2 (60.7-75.1) | Na |
| Less interest sex | 35.3 (28.2-42.9) | 30.1 (23.3-37.5) | 23.1 (17.1-30.1) | 11.6 (7.2-17.3) | 64.7 (57.1-71.8) | 34.7 (27.6-42.3) |
| Less pleasure sex | 45.3 (37.7-53.1) | 27.6 (21.1-35.0) | 18.8 (13.2-25.5) | 8.2 (4.6-13.4) | 54.7 (46.9-62.3) | 27.1 (20.5-34.4) |
| Pain during/after sex | 77.4 (70.3-83.5) | 14.9 (9.9-21.2) | 4.8 (2.1-9.2) | 3.0 (1.0-6.8) | 22.6 (16.5-29.7) | 7.7 (4.2-12.9) |
| Difficult erection | 53.9 (42.1-65.5) | 28.9 (19.1-40.5) | 10.5 (4.7-19.7) | 6.6 (2.2-14.7) | 46.1 (34.5-57.9) | 17.1 (9.4-27.5) |
| Dry vagina | 48.8 (37.9-59.9) | 25.6 (16.8-36.1) | 18.6 (11.0-28.4) | 7.0 (2.6-14.6) | 51.2 (40.1-62.1) | 25.6 (16.8-36.1) |

* At T4 as not available at T1. $ Moderate-severe includes ‘Quite a bit’ and ‘A lot’. Abbreviations: Na: not applicable

# Mean and median symptom scores

Table 26 Mean and median symptoms scores for 7 scales plus 43 single items, in 12 patients on asciminib, and 14 patients on bosutinib at T1

| Item | Asciminib | | | | | | Bosutinib | | | | | |
| --- | --- | --- | --- | --- | --- | --- | --- | --- | --- | --- | --- | --- |
|  | Mean | Lower 95%CI | Higher 95%CI | Median | IQR | Skewness | Mean | Lower 95%CI | Higher 95%CI | Median | IQR | Skewness |
| Fatigue (scale) | 40.2 | 23.8 | 56.7 | 38.9 | 26.4 | 0.5 | 42.8 | 29.0 | 56.6 | 41.6 | 40.2 | 0.4 |
| Headaches | 26.6 | 11.6 | 41.7 | 33.3 | 25.0 | 0.1 | 12.1 | 0.0 | 27.2 | 0.0 | 16.7 | 1.5 |
| Dizzy | 33.3 | 4.7 | 61.9 | 33.3 | 66.6 | 0.6 | 12.1 | 0.8 | 23.4 | 0.0 | 33.3 | 0.6 |
| Cognitive problems (scale) | 25.9 | 9.9 | 41.9 | 22.2 | 22.2 | 0.7 | 39.4 | 18.7 | 60.0 | 33.3 | 55.5 | 0.1 |
| Bruised easily | 33.3 | 0.0 | 67.2 | 0.0 | 66.6 | 0.7 | 33.3 | 8.8 | 57.8 | 33.3 | 66.6 | 0.5 |
| Skin problems (scale) | 28.9 | 11.1 | 46.6 | 26.6 | 20.0 | 0.7 | 27.9 | 12.1 | 43.6 | 26.6 | 33.3 | 0.3 |
| Wounds healing problems | 7.4 | 0.0 | 18.7 | 0.0 | 0.0 | 1.3 | 18.2 | 0.0 | 39.1 | 0.0 | 33.3 | 1.8 |
| Hair loss | 22.2 | 0.0 | 47.8 | 0.0 | 33.3 | 1.5 | 33.3 | 13.3 | 53.3 | 33.3 | 16.7 | 0.9 |
| Nail problems* | 16.7 | 0.0 | 45.9 | 0.0 | 25.0 | 1.1 | 16.7 | 0.0 | 47.2 | 16.7 | 33.3 | 0.0 |
| Eye problems (scale) | 28.5 | 12.1 | 44.8 | 33.3 | 33.3 | -0.3 | 21.8 | 10.3 | 33.3 | 13.3 | 20.0 | 0.6 |
| Swelling face/eyes | 14.8 | 0.0 | 40.8 | 0.0 | 0.0 | 2.1 | 15.1 | 0.0 | 30.5 | 0.0 | 33.3 | 1.1 |
| Swelling body parts | 4.2 | 0.0 | 14.0 | 0.0 | 0.0 | 2.3 | 30.3 | 9.2 | 51.4 | 33.3 | 66.6 | 0.2 |
| Hearing problems | 16.7 | 0.0 | 42.4 | 0.0 | 16.7 | 1.2 | 23.3 | 0.0 | 48.6 | 0.0 | 33.3 | 1.2 |
| Nose bleeds | 25.0 | 0.0 | 53.8 | 16.7 | 33.3 | 1.3 | 9.1 | 0.0 | 23.5 | 0.0 | 0.0 | 2.1 |
| Other nose problems* | 40.0 | 0.0 | 85.3 | 66.6 | 66.6 | -0.4 | 46.6 | 0.0 | 100.0 | 66.6 | 66.6 | -0.1 |
| Dry mouth | 37.5 | 9.9 | 65.1 | 33.3 | 16.7 | 0.7 | 24.2 | 6.6 | 41.8 | 33.3 | 33.3 | 0.5 |
| Pain/soreness mouth | 25.0 | 0.3 | 49.7 | 16.7 | 41.6 | 0.5 | 15.1 | 0.0 | 36.1 | 0.0 | 16.7 | 2.1 |
| Teeth problems | 16.7 | 0.0 | 37.7 | 0.0 | 33.3 | 1.1 | 20.0 | 0.0 | 43.0 | 0.0 | 50.0 | 0.9 |
| Bleeding gums | 8.3 | 0.0 | 28.0 | 0.0 | 0.0 | 2.3 | 16.7 | 0.0 | 39.8 | 0.0 | 25.0 | 1.9 |
| Taste change | 25.0 | 5.3 | 44.7 | 33.3 | 33.3 | 0.3 | 21.2 | 0.0 | 44.2 | 0.0 | 33.3 | 1.4 |
| Appetite loss | 33.3 | 3.5 | 63.1 | 33.3 | 41.6 | 0.8 | 18.2 | 0.0 | 39.1 | 0.0 | 33.3 | 1.8 |
| Mood problems (scale) | 37.2 | 16.2 | 58.2 | 33.3 | 33.3 | 0.8 | 22.8 | 9.2 | 36.5 | 16.7 | 40.2 | 0.5 |
| Palpitations | 33.3 | 3.5 | 63.1 | 33.3 | 41.6 | 0.8 | 21.2 | 0.5 | 41.9 | 0.0 | 33.3 | 1.6 |
| Pain in chest | 25.0 | 0.0 | 53.8 | 16.7 | 33.3 | 1.3 | 15.1 | 0.0 | 30.5 | 0.0 | 33.3 | 1.1 |
| Short of breath | 45.8 | 16.3 | 75.3 | 50.0 | 41.6 | 0.0 | 30.3 | 6.9 | 53.7 | 33.3 | 50.0 | 0.7 |
| Coughed | 45.8 | 16.3 | 75.3 | 50.0 | 41.6 | 0.0 | 27.2 | 7.7 | 46.8 | 33.3 | 50.0 | 0.4 |
| Feeling bloated | 45.8 | 3.9 | 87.8 | 33.3 | 100.0 | 0.1 | 42.4 | 15.7 | 69.1 | 33.3 | 66.6 | 0.2 |
| Indigestion | 33.3 | 3.5 | 63.1 | 33.3 | 41.6 | 0.8 | 39.4 | 9.6 | 69.1 | 33.3 | 83.3 | 0.5 |
| Nausea | 29.1 | 1.5 | 56.7 | 16.7 | 66.6 | 0.3 | 20.0 | 0.0 | 45.6 | 0.0 | 25.0 | 1.4 |
| Vomited | 8.3 | 0.0 | 28.0 | 0.0 | 0.0 | 2.3 | 12.1 | 0.0 | 27.2 | 0.0 | 16.7 | 1.5 |
| Diarrhoea | 25.0 | 0.0 | 63.7 | 0.0 | 25.0 | 1.2 | 33.3 | 5.0 | 61.6 | 0.0 | 66.6 | 0.6 |
| Obstipation | 29.2 | 1.5 | 56.8 | 33.3 | 33.3 | 1.2 | 20.0 | 0.0 | 43.0 | 0.0 | 33.3 | 1.7 |
| Abdominal pain/cramps | 41.6 | 9.2 | 74.1 | 50.0 | 66.6 | 0.1 | 43.3 | 9.5 | 77.1 | 33.3 | 91.7 | 0.2 |
| Flatulence | 29.2 | 1.5 | 56.8 | 33.3 | 33.3 | 1.2 | 70.0 | 49.1 | 90.9 | 66.6 | 58.4 | -0.2 |
| Frequent urination | 37.5 | 0.0 | 75.3 | 16.7 | 75.0 | 0.5 | 30.0 | 12.4 | 47.5 | 33.3 | 25.0 | 0.1 |
| Muscle/joint cramps/pain (scale) | 36.2 | 17.4 | 55.0 | 36.6 | 32.5 | 0.2 | 41.3 | 20.8 | 61.8 | 46.6 | 56.7 | -0.2 |
| Pail/cold fingers/toes* | 20.0 | 0.0 | 75.5 | 0.0 | 0.0 | 1.5 | 40.0 | 0.0 | 100.0 | 0.0 | 100.0 | 0.4 |
| Tingling/numbness hands/feet | 20.8 | 0.1 | 41.5 | 16.7 | 33.3 | 0.7 | 23.3 | 0.7 | 45.9 | 16.7 | 33.3 | 1.5 |
| Problems finger dexterity | 20.8 | 0.1 | 41.5 | 16.7 | 33.3 | 0.7 | 10.0 | 0.0 | 21.5 | 0.0 | 25.0 | 0.9 |
| Back pain* | 33.3 | 0.0 | 84.0 | 33.3 | 33.3 | 0.9 | 20.0 | 0.0 | 57.0 | 0.0 | 33.3 | 0.8 |
| Fever/chills | 25.0 | 0.0 | 53.8 | 16.7 | 33.3 | 1.3 | 12.1 | 0.0 | 27.2 | 0.0 | 16.7 | 1.5 |
| Sweating excessively | 25.0 | 0.0 | 57.4 | 0.0 | 41.6 | 1.1 | 21.2 | 0.5 | 41.9 | 0.0 | 50.0 | 0.8 |
| Problems tolerating heat/cold | 20.8 | 0.0 | 50.4 | 0.0 | 33.3 | 1.6 | 26.6 | 7.8 | 45.4 | 33.3 | 33.3 | 0.3 |
| Hot flushes | 20.8 | 0.0 | 50.4 | 0.0 | 33.3 | 1.6 | 16.7 | 0.0 | 33.5 | 0.0 | 33.3 | 1.0 |
| Airway infections* | 6.7 | 0.0 | 25.2 | 0.0 | 0.0 | 1.5 | 0.0 | NA | NA | 0.0 | 0.0 | NA |
| Other infections* | 0.0 | NA | NA | 0.0 | 0.0 | NA | 13.3 | 0.0 | 50.3 | 0.0 | 0.0 | 1.5 |
| Sore/enlarged nipples/breasts | 0.0 | NA | NA | 0.0 | 0.0 | NA | 0.0 | NA | NA | 0.0 | 0.0 | NA |
| Sexuality problems (scale) | 25.0 | 8.7 | 41.2 | 27.8 | 36.1 | -0.2 | 30.0 | 8.8 | 51.2 | 27.8 | 47.2 | 0.7 |
| Difficulty getting/maintaining erection | 33.3 | 0.0 | 76.6 | 33.3 | 16.7 | 0.0 | 33.3 | 0.0 | 100.0 | 33.3 | 33.3 | 0.0 |
| Dry vagina | 8.3 | 0.0 | 34.8 | 0.0 | 8.3 | 1.2 | 29.1 | 5.9 | 52.4 | 33.3 | 41.6 | 0.2 |

* At T4 as not available at T1. Abbreviations: CI: confidence interval; IQR: inter quartile range. Shaded cells indicate a skewness of ≥2.0. A skewness of <1.0 is seen as excellent, and <2.0 as acceptable [9].

Table 27 Mean and median symptoms scores for 7 scales plus 43 single items, in 70 patients on dasatinib, and 84 patients on imatinib at T1

| Item | Dasatinib | | | | | | Imatinib | | | | | |
| --- | --- | --- | --- | --- | --- | --- | --- | --- | --- | --- | --- | --- |
|  | Mean | Lower 95%CI | Higher 95%CI | Median | IQR | Skewness | Mean | Lower 95%CI | Higher 95%CI | Median | IQR | Skewness |
| Fatigue (scale) | 46.3 | 39.8 | 52.9 | 47.2 | 31.9 | 0.1 | 39.0 | 33.7 | 44.3 | 38.9 | 44.4 | 0.0 |
| Headaches | 23.7 | 16.1 | 31.3 | 0.0 | 33.3 | 1.1 | 12.6 | 7.7 | 17.5 | 0.0 | 33.3 | 1.4 |
| Dizzy | 14.9 | 9.0 | 20.9 | 0.0 | 33.3 | 1.5 | 11.3 | 7.2 | 15.3 | 0.0 | 33.3 | 1.2 |
| Cognitive problems (scale) | 30.5 | 23.6 | 37.5 | 22.2 | 33.3 | 0.7 | 21.1 | 16.3 | 25.9 | 11.1 | 33.3 | 0.6 |
| Bruised easily | 12.9 | 6.9 | 18.8 | 0.0 | 33.3 | 1.8 | 21.7 | 15.7 | 27.8 | 0.0 | 33.3 | 0.9 |
| Skin problems (scale) | 28.5 | 23.4 | 33.5 | 26.6 | 20.0 | 0.4 | 19.5 | 15.1 | 24.0 | 13.3 | 20.0 | 1.2 |
| Wounds healing problems | 11.1 | 5.0 | 17.2 | 0.0 | 0.0 | 2.1 | 10.2 | 5.7 | 14.7 | 0.0 | 33.3 | 2.2 |
| Hair loss | 26.8 | 19.1 | 34.4 | 33.3 | 41.6 | 0.6 | 11.6 | 6.6 | 16.5 | 0.0 | 33.3 | 1.9 |
| Nail problems* | 8.8 | 1.5 | 16.0 | 0.0 | 16.7 | 1.1 | 25.0 | 11.1 | 38.9 | 0.0 | 66.6 | 0.8 |
| Eye problems (scale) | 22.2 | 16.9 | 27.5 | 13.3 | 26.7 | 0.6 | 27.3 | 22.9 | 31.8 | 26.6 | 26.6 | 0.5 |
| Swelling face/eyes | 20.0 | 11.4 | 28.6 | 0.0 | 33.3 | 1.4 | 38.9 | 31.3 | 46.4 | 33.3 | 66.6 | 0.2 |
| Swelling body parts | 19.1 | 12.1 | 26.1 | 0.0 | 33.3 | 1.1 | 22.0 | 15.4 | 28.7 | 0.0 | 33.3 | 1.0 |
| Hearing problems | 7.7 | 3.2 | 12.2 | 0.0 | 0.0 | 2.1 | 13.9 | 8.0 | 19.7 | 0.0 | 33.3 | 1.8 |
| Nose bleeds | 3.0 | 0.4 | 5.5 | 0.0 | 0.0 | 2.9 | 3.3 | 0.9 | 5.6 | 0.0 | 0.0 | 2.7 |
| Other nose problems* | 25.0 | 11.7 | 38.2 | 16.7 | 41.6 | 0.5 | 20.8 | 10.0 | 31.6 | 0.0 | 33.3 | 0.7 |
| Dry mouth | 19.0 | 11.5 | 26.6 | 0.0 | 33.3 | 1.5 | 25.2 | 17.6 | 32.8 | 0.0 | 33.3 | 1.0 |
| Pain/soreness mouth | 9.5 | 4.2 | 14.8 | 0.0 | 0.0 | 2.5 | 9.9 | 5.2 | 14.5 | 0.0 | 0.0 | 2.3 |
| Teeth problems | 8.5 | 3.5 | 13.4 | 0.0 | 0.0 | 2.1 | 13.1 | 7.6 | 18.7 | 0.0 | 33.3 | 2.0 |
| Bleeding gums | 5.9 | 1.7 | 10.1 | 0.0 | 0.0 | 2.7 | 9.8 | 5.8 | 13.9 | 0.0 | 33.3 | 1.5 |
| Taste change | 9.7 | 3.8 | 15.6 | 0.0 | 0.0 | 2.4 | 15.5 | 8.8 | 22.1 | 0.0 | 33.3 | 1.8 |
| Appetite loss | 10.7 | 5.3 | 16.1 | 0.0 | 8.3 | 1.7 | 17.1 | 10.4 | 23.9 | 0.0 | 33.3 | 1.5 |
| Mood problems (scale) | 24.2 | 18.3 | 30.1 | 16.7 | 27.8 | 1.2 | 24.3 | 18.6 | 30.1 | 16.7 | 33.3 | 1.2 |
| Palpitations | 23.2 | 15.4 | 31.0 | 0.0 | 33.3 | 1.0 | 20.0 | 14.2 | 25.8 | 0.0 | 33.3 | 0.8 |
| Pain in chest | 7.1 | 3.1 | 11.2 | 0.0 | 0.0 | 2.0 | 8.3 | 3.8 | 12.8 | 0.0 | 0.0 | 2.7 |
| Short of breath | 20.8 | 14.3 | 27.3 | 16.7 | 33.3 | 1.0 | 27.1 | 20.3 | 33.9 | 33.3 | 33.3 | 0.6 |
| Coughed | 13.1 | 6.3 | 19.8 | 0.0 | 33.3 | 2.0 | 13.2 | 7.8 | 18.6 | 0.0 | 33.3 | 1.7 |
| Feeling bloated | 30.9 | 23.0 | 38.9 | 33.3 | 41.6 | 0.6 | 22.4 | 15.6 | 29.1 | 0.0 | 33.3 | 1.0 |
| Indigestion | 19.4 | 11.3 | 27.5 | 0.0 | 33.3 | 1.4 | 21.7 | 14.6 | 28.8 | 0.0 | 33.3 | 1.1 |
| Nausea | 16.1 | 8.8 | 23.3 | 0.0 | 33.3 | 1.6 | 20.1 | 14.8 | 25.3 | 33.3 | 33.3 | 0.6 |
| Vomited | 3.0 | 0.0 | 6.1 | 0.0 | 0.0 | 4.1 | 7.7 | 4.1 | 11.4 | 0.0 | 0.0 | 1.7 |
| Diarrhoea | 16.7 | 10.1 | 23.2 | 0.0 | 33.3 | 1.6 | 30.9 | 22.3 | 39.5 | 33.3 | 66.6 | 0.8 |
| Obstipation | 22.6 | 14.1 | 31.1 | 0.0 | 33.3 | 1.3 | 8.7 | 4.4 | 12.9 | 0.0 | 0.0 | 1.9 |
| Abdominal pain/cramps | 24.8 | 16.5 | 33.2 | 33.3 | 33.3 | 1.2 | 21.7 | 15.9 | 27.5 | 33.3 | 33.3 | 0.6 |
| Flatulence | 34.5 | 25.4 | 43.7 | 33.3 | 66.6 | 0.5 | 36.7 | 29.5 | 44.0 | 33.3 | 66.6 | 0.4 |
| Frequent urination | 35.7 | 26.1 | 45.4 | 33.3 | 66.6 | 0.5 | 29.9 | 22.5 | 37.3 | 33.3 | 66.6 | 0.6 |
| Muscle/joint cramps/pain (scale) | 25.5 | 19.7 | 31.3 | 25.0 | 33.3 | 0.9 | 31.3 | 25.7 | 36.9 | 26.6 | 34.1 | 0.5 |
| Pail/cold fingers/toes* | 40.0 | 20.6 | 59.3 | 33.3 | 66.6 | 0.3 | 26.4 | 12.0 | 40.7 | 0.0 | 41.6 | 0.9 |
| Tingling/numbness hands/feet | 35.1 | 25.3 | 45.0 | 33.3 | 66.6 | 0.6 | 26.4 | 19.4 | 33.5 | 33.3 | 33.3 | 0.8 |
| Problems finger dexterity | 11.7 | 5.3 | 18.1 | 0.0 | 0.0 | 2.0 | 14.2 | 8.4 | 20.0 | 0.0 | 33.3 | 1.8 |
| Back pain* | 31.6 | 19.8 | 43.5 | 33.3 | 41.6 | 0.1 | 16.7 | 6.5 | 26.8 | 0.0 | 33.3 | 1.8 |
| Fever/chills | 11.7 | 6.1 | 17.3 | 0.0 | 33.3 | 1.5 | 8.8 | 4.3 | 13.4 | 0.0 | 0.0 | 2.5 |
| Sweating excessively | 14.5 | 8.3 | 20.7 | 0.0 | 33.3 | 1.3 | 17.1 | 11.2 | 23.1 | 0.0 | 33.3 | 1.3 |
| Problems tolerating heat/cold | 21.8 | 14.6 | 29.0 | 0.0 | 33.3 | 1.1 | 21.6 | 14.9 | 28.2 | 0.0 | 33.3 | 1.2 |
| Hot flushes | 17.0 | 9.6 | 24.3 | 0.0 | 33.3 | 1.5 | 7.3 | 3.0 | 11.7 | 0.0 | 0.0 | 3.0 |
| Airway infections* | 6.7 | 0.0 | 14.8 | 0.0 | 0.0 | 2.5 | 20.8 | 6.0 | 35.7 | 0.0 | 33.3 | 1.5 |
| Other infections* | 8.3 | 0.0 | 19.5 | 0.0 | 0.0 | 3.1 | 8.3 | 0.0 | 18.7 | 0.0 | 0.0 | 2.9 |
| Sore/enlarged nipples/breasts | 2.5 | 0.1 | 4.9 | 0.0 | 0.0 | 3.3 | 4.5 | 1.7 | 7.3 | 0.0 | 0.0 | 2.1 |
| Sexuality problems (scale) | 29.1 | 21.3 | 36.9 | 22.2 | 33.3 | 1.2 | 25.3 | 18.5 | 32.1 | 22.2 | 44.4 | 0.9 |
| Difficulty getting/maintaining erection | 19.0 | 5.0 | 33.1 | 0.0 | 33.3 | 1.7 | 18.6 | 9.0 | 28.2 | 0.0 | 33.3 | 1.3 |
| Dry vagina | 30.8 | 16.3 | 45.4 | 0.0 | 66.6 | 0.7 | 24.1 | 12.4 | 35.8 | 0.0 | 33.3 | 0.9 |

* At T4 as not available at T1. Abbreviations: CI: confidence interval; IQR: inter quartile range. Shaded cells indicate a skewness of ≥2.0. A skewness of <1.0 is seen as excellent, and <2.0 as acceptable [9].

Table 28 Mean and median symptoms scores for 7 scales plus 43 single items, in 39 patients on nilotinib, and 8 patients on ponatinib at T1

| Item | Nilotinib | | | | | | Ponatinib | | | | | |
| --- | --- | --- | --- | --- | --- | --- | --- | --- | --- | --- | --- | --- |
|  | Mean | Lower 95%CI | Higher 95%CI | Median | IQR | Skewness | Mean | Lower 95%CI | Higher 95%CI | Median | IQR | Skewness |
| Fatigue (scale) | 38.5 | 31.2 | 45.7 | 38.9 | 33.3 | 0.0 | 40.5 | 20.2 | 60.8 | 40.0 | 31.9 | -0.2 |
| Headaches | 7.4 | 1.9 | 12.9 | 0.0 | 0.0 | 2.1 | 4.8 | 0.0 | 16.4 | 0.0 | 0.0 | 2.0 |
| Dizzy | 12.0 | 5.3 | 18.7 | 0.0 | 33.3 | 1.4 | 9.5 | 0.0 | 24.5 | 0.0 | 16.7 | 0.9 |
| Cognitive problems (scale) | 16.5 | 10.4 | 22.6 | 11.1 | 33.3 | 0.9 | 28.5 | 11.9 | 45.2 | 33.3 | 11.1 | -0.3 |
| Bruised easily | 9.5 | 4.3 | 14.8 | 0.0 | 33.3 | 0.9 | 4.8 | 0.0 | 16.4 | 0.0 | 0.0 | 2.0 |
| Skin problems (scale) | 24.2 | 16.8 | 31.7 | 20.0 | 36.7 | 0.6 | 28.5 | 13.6 | 43.5 | 26.6 | 26.6 | -0.1 |
| Wounds healing problems | 1.0 | 0.0 | 2.9 | 0.0 | 0.0 | 5.7 | 5.6 | 0.0 | 19.8 | 0.0 | 0.0 | 1.8 |
| Hair loss | 24.5 | 13.7 | 35.3 | 16.7 | 33.3 | 1.2 | 9.5 | 0.0 | 24.5 | 0.0 | 16.7 | 0.9 |
| Nail problems* | 5.1 | 0.0 | 12.7 | 0.0 | 0.0 | 1.9 | 0.0 | NA | NA | 0.0 | 0.0 | NA |
| Eye problems (scale) | 21.3 | 15.4 | 27.2 | 20.0 | 24.6 | 1.4 | 17.1 | 4.4 | 29.9 | 20.0 | 16.7 | 0.4 |
| Swelling face/eyes | 11.8 | 4.2 | 19.3 | 0.0 | 25.0 | 1.6 | 4.8 | 0.0 | 16.4 | 0.0 | 0.0 | 2.0 |
| Swelling body parts | 7.3 | 0.6 | 13.9 | 0.0 | 0.0 | 2.4 | 5.6 | 0.0 | 19.8 | 0.0 | 0.0 | 1.8 |
| Hearing problems | 9.1 | 1.1 | 17.1 | 0.0 | 0.0 | 2.7 | 0.0 | NA | NA | 0.0 | 0.0 | NA |
| Nose bleeds | 7.1 | 0.0 | 14.2 | 0.0 | 0.0 | 3.4 | 0.0 | NA | NA | 0.0 | 0.0 | NA |
| Other nose problems* | 19.0 | 2.7 | 35.4 | 0.0 | 33.3 | 0.9 | 33.3 | NA | NA | 33.3 | 0.0 | NA |
| Dry mouth | 18.2 | 7.9 | 28.4 | 0.0 | 33.3 | 1.6 | 33.3 | 15.5 | 51.1 | 33.3 | 0.0 | 0.0 |
| Pain/soreness mouth | 7.1 | 0.0 | 14.1 | 0.0 | 0.0 | 2.6 | 4.8 | 0.0 | 16.4 | 0.0 | 0.0 | 2.0 |
| Teeth problems | 4.0 | 0.0 | 8.9 | 0.0 | 0.0 | 3.5 | 19.0 | 2.6 | 35.5 | 33.3 | 33.3 | -0.3 |
| Bleeding gums | 5.2 | 0.8 | 9.6 | 0.0 | 0.0 | 1.9 | 14.3 | 0.0 | 30.7 | 0.0 | 33.3 | 0.3 |
| Taste change | 7.1 | 1.3 | 12.8 | 0.0 | 0.0 | 2.2 | 11.1 | 0.0 | 39.6 | 0.0 | 0.0 | 1.8 |
| Appetite loss | 7.1 | 0.6 | 13.5 | 0.0 | 0.0 | 2.5 | 11.1 | 0.0 | 29.1 | 0.0 | 25.0 | 0.7 |
| Mood problems (scale) | 16.4 | 11.1 | 21.7 | 11.1 | 22.2 | 1.0 | 16.7 | 5.6 | 27.7 | 22.2 | 16.7 | -0.6 |
| Palpitations | 15.1 | 5.8 | 24.5 | 0.0 | 33.3 | 1.7 | 16.7 | 0.0 | 35.8 | 16.7 | 33.3 | 0.0 |
| Pain in chest | 6.1 | 0.0 | 12.3 | 0.0 | 0.0 | 2.8 | 5.6 | 0.0 | 19.8 | 0.0 | 0.0 | 1.8 |
| Short of breath | 14.1 | 6.3 | 22.0 | 0.0 | 33.3 | 1.3 | 5.6 | 0.0 | 19.8 | 0.0 | 0.0 | 1.8 |
| Coughed | 16.1 | 7.7 | 24.6 | 0.0 | 33.3 | 1.1 | 0.0 | NA | NA | 0.0 | 0.0 | NA |
| Feeling bloated | 14.1 | 6.3 | 22.0 | 0.0 | 33.3 | 1.3 | 33.3 | 2.0 | 64.6 | 33.3 | 50.0 | 0.0 |
| Indigestion | 11.1 | 3.5 | 18.7 | 0.0 | 0.0 | 1.7 | 33.3 | 11.2 | 55.4 | 33.3 | 0.0 | 0.0 |
| Nausea | 10.4 | 3.3 | 17.5 | 0.0 | 8.3 | 1.7 | 16.7 | 0.0 | 45.9 | 0.0 | 25.0 | 1.1 |
| Vomited | 2.1 | 0.0 | 5.0 | 0.0 | 0.0 | 3.6 | 0.0 | NA | NA | 0.0 | 0.0 | NA |
| Diarrhoea | 7.3 | 0.0 | 14.6 | 0.0 | 0.0 | 3.4 | 5.6 | 0.0 | 19.8 | 0.0 | 0.0 | 1.8 |
| Obstipation | 18.7 | 6.9 | 30.5 | 0.0 | 33.3 | 1.6 | 16.7 | 0.0 | 35.8 | 16.7 | 33.3 | 0.0 |
| Abdominal pain/cramps | 16.7 | 7.0 | 26.3 | 0.0 | 33.3 | 1.5 | 16.7 | 0.0 | 35.8 | 16.7 | 33.3 | 0.0 |
| Flatulence | 32.3 | 21.1 | 43.5 | 33.3 | 66.6 | 0.3 | 22.2 | 0.0 | 50.7 | 16.7 | 33.3 | 0.6 |
| Frequent urination | 26.0 | 14.7 | 37.3 | 0.0 | 66.6 | 0.7 | 38.9 | 12.5 | 65.2 | 33.3 | 25.0 | -0.2 |
| Muscle/joint cramps/pain (scale) | 23.1 | 15.0 | 31.1 | 20.0 | 28.3 | 0.9 | 21.1 | 10.8 | 31.4 | 20.0 | 5.0 | 1.3 |
| Pail/cold fingers/toes* | 7.1 | 0.0 | 15.3 | 0.0 | 0.0 | 1.4 | 33.3 | NA | NA | 33.3 | 0.0 | NA |
| Tingling/numbness hands/feet | 16.7 | 9.2 | 24.1 | 0.0 | 33.3 | 0.8 | 33.3 | 11.2 | 55.4 | 33.3 | 0.0 | 0.0 |
| Problems finger dexterity | 6.2 | 1.5 | 11.0 | 0.0 | 0.0 | 1.6 | 11.1 | 0.0 | 29.1 | 0.0 | 25.0 | 0.7 |
| Back pain* | 30.9 | 10.3 | 51.6 | 33.3 | 33.3 | 0.9 | 0.0 | NA | NA | 0.0 | 0.0 | NA |
| Fever/chills | 4.2 | 0.1 | 8.2 | 0.0 | 0.0 | 2.3 | 11.1 | 0.0 | 29.1 | 0.0 | 25.0 | 0.7 |
| Sweating excessively | 22.9 | 12.6 | 33.2 | 0.0 | 33.3 | 1.0 | 11.1 | 0.0 | 29.1 | 0.0 | 25.0 | 0.7 |
| Problems tolerating heat/cold | 14.6 | 5.0 | 24.2 | 0.0 | 33.3 | 1.7 | 11.1 | 0.0 | 29.1 | 0.0 | 25.0 | 0.7 |
| Hot flushes | 13.5 | 3.0 | 24.0 | 0.0 | 0.0 | 2.1 | 16.7 | 0.0 | 45.9 | 0.0 | 25.0 | 1.1 |
| Airway infections* | 4.8 | 0.0 | 11.7 | 0.0 | 0.0 | 2.0 | 0.0 | NA | NA | 0.0 | 0.0 | NA |
| Other infections* | 2.4 | 0.0 | 7.5 | 0.0 | 0.0 | 3.3 | 0.0 | NA | NA | 0.0 | 0.0 | NA |
| Sore/enlarged nipples/breasts | 5.2 | 0.0 | 10.6 | 0.0 | 0.0 | 2.9 | 0.0 | NA | NA | 0.0 | 0.0 | NA |
| Sexuality problems (scale) | 26.5 | 16.7 | 36.4 | 22.2 | 44.4 | 0.6 | 29.6 | 6.7 | 52.5 | 22.2 | 25.0 | 0.9 |
| Difficulty getting/maintaining erection | 41.6 | 19.3 | 64.0 | 33.3 | 41.6 | 0.5 | 11.1 | 0.0 | 58.9 | 0.0 | 16.7 | 0.7 |
| Dry vagina | 33.3 | 16.2 | 50.4 | 33.3 | 33.3 | 0.8 | 33.3 | NA | NA | 33.3 | 0.0 | NA |

* At T4 as not available at T1. Abbreviations: CI: confidence interval; IQR: inter quartile range. Shaded cells indicate a skewness of ≥2.0. A skewness of <1.0 is seen as excellent, and <2.0 as acceptable [9].

Table 29 Mean and median symptoms scores for 7 scales plus 43 single items, in 40 patients in treatment-free remission at T1, and 227 patients on any TKI at T1

| Item | Treatment-free remission | | | | | | Any TKI | | | | | |
| --- | --- | --- | --- | --- | --- | --- | --- | --- | --- | --- | --- | --- |
|  | Mean | Lower 95%CI | Higher 95%CI | Median | IQR | Skewness | Mean | Lower 95%CI | Higher 95%CI | Median | IQR | Skewness |
| Fatigue (scale) | 40.5 | 32.3 | 48.8 | 41.6 | 34.0 | 0.2 | 41.5 | 38.2 | 44.8 | 38.9 | 38.9 | 0.1 |
| Headaches | 14.8 | 5.7 | 23.9 | 0.0 | 33.3 | 1.7 | 15.4 | 12.1 | 18.7 | 0.0 | 33.3 | 1.5 |
| Dizzy | 12.4 | 4.5 | 20.3 | 0.0 | 33.3 | 2.1 | 13.5 | 10.5 | 16.5 | 0.0 | 33.3 | 1.5 |
| Cognitive problems (scale) | 22.5 | 14.7 | 30.4 | 22.2 | 33.3 | 1.3 | 24.6 | 21.3 | 27.9 | 22.2 | 33.3 | 0.8 |
| Bruised easily | 6.5 | 1.2 | 11.7 | 0.0 | 0.0 | 2.4 | 17.4 | 13.8 | 21.1 | 0.0 | 33.3 | 1.4 |
| Skin problems (scale) | 20.6 | 14.5 | 26.7 | 13.3 | 23.3 | 0.8 | 24.3 | 21.4 | 27.2 | 20.0 | 26.6 | 0.7 |
| Wounds healing problems | 7.4 | 0.2 | 14.6 | 0.0 | 0.0 | 3.1 | 8.9 | 6.1 | 11.7 | 0.0 | 0.0 | 2.5 |
| Hair loss | 13.9 | 6.1 | 21.7 | 0.0 | 33.3 | 1.4 | 20.1 | 16.2 | 24.0 | 0.0 | 33.3 | 1.3 |
| Nail problems* | 20.0 | 0.0 | 43.0 | 0.0 | 33.3 | 1.7 | 14.7 | 8.7 | 20.7 | 0.0 | 33.3 | 1.5 |
| Eye problems (scale) | 16.2 | 10.1 | 22.3 | 13.3 | 26.6 | 1.0 | 24.1 | 21.4 | 26.8 | 20.0 | 30.0 | 0.6 |
| Swelling face/eyes | 12.7 | 2.8 | 22.7 | 0.0 | 0.0 | 2.2 | 24.6 | 20.1 | 29.1 | 0.0 | 33.3 | 1.0 |
| Swelling body parts | 23.6 | 13.6 | 33.7 | 33.3 | 33.3 | 0.9 | 17.7 | 13.9 | 21.4 | 0.0 | 33.3 | 1.2 |
| Hearing problems | 17.6 | 6.1 | 29.2 | 0.0 | 25.0 | 1.6 | 11.3 | 8.0 | 14.6 | 0.0 | 0.0 | 2.1 |
| Nose bleeds | 4.9 | 0.0 | 10.0 | 0.0 | 0.0 | 3.0 | 5.0 | 2.8 | 7.2 | 0.0 | 0.0 | 3.8 |
| Other nose problems* | 29.1 | 11.3 | 47.0 | 33.3 | 8.3 | 0.1 | 25.1 | 18.0 | 32.1 | 0.0 | 66.6 | 0.6 |
| Dry mouth | 17.6 | 8.9 | 26.3 | 0.0 | 33.3 | 1.0 | 22.9 | 18.6 | 27.2 | 0.0 | 33.3 | 1.2 |
| Pain/soreness mouth | 9.8 | 3.7 | 15.9 | 0.0 | 25.0 | 1.5 | 10.0 | 7.0 | 13.1 | 0.0 | 0.0 | 2.3 |
| Teeth problems | 10.8 | 3.9 | 17.6 | 0.0 | 25.0 | 1.6 | 10.9 | 7.8 | 13.9 | 0.0 | 0.0 | 2.0 |
| Bleeding gums | 7.1 | 0.6 | 13.5 | 0.0 | 0.0 | 2.5 | 8.3 | 5.8 | 10.9 | 0.0 | 0.0 | 2.2 |
| Taste change | 3.9 | 0.0 | 8.7 | 0.0 | 0.0 | 3.6 | 12.9 | 9.2 | 16.5 | 0.0 | 33.3 | 2.0 |
| Appetite loss | 4.0 | 0.0 | 8.9 | 0.0 | 0.0 | 3.5 | 13.9 | 10.3 | 17.6 | 0.0 | 33.3 | 1.8 |
| Mood problems (scale) | 22.8 | 15.0 | 30.7 | 22.2 | 19.4 | 2.0 | 23.1 | 19.9 | 26.3 | 16.7 | 27.8 | 1.2 |
| Palpitations | 16.1 | 8.8 | 23.4 | 0.0 | 33.3 | 0.9 | 20.6 | 16.7 | 24.5 | 0.0 | 33.3 | 1.1 |
| Pain in chest | 4.2 | 0.1 | 8.2 | 0.0 | 0.0 | 2.3 | 8.6 | 5.9 | 11.3 | 0.0 | 0.0 | 2.5 |
| Short of breath | 16.7 | 6.5 | 26.8 | 0.0 | 33.3 | 1.5 | 23.2 | 19.2 | 27.1 | 0.0 | 33.3 | 0.9 |
| Coughed | 12.5 | 4.0 | 21.0 | 0.0 | 33.3 | 2.1 | 15.6 | 11.9 | 19.2 | 0.0 | 33.3 | 1.5 |
| Feeling bloated | 17.7 | 6.7 | 28.7 | 0.0 | 33.3 | 1.6 | 26.1 | 21.6 | 30.5 | 33.3 | 33.3 | 0.9 |
| Indigestion | 16.7 | 6.5 | 26.8 | 0.0 | 33.3 | 1.5 | 21.0 | 16.7 | 25.4 | 0.0 | 33.3 | 1.2 |
| Nausea | 4.3 | 0.1 | 8.5 | 0.0 | 0.0 | 2.2 | 17.4 | 13.8 | 21.0 | 0.0 | 33.3 | 1.3 |
| Vomited | 0.0 | NA | NA | 0.0 | 0.0 | NA | 5.3 | 3.3 | 7.4 | 0.0 | 0.0 | 2.7 |
| Diarrhoea | 11.5 | 3.0 | 19.9 | 0.0 | 8.3 | 2.3 | 21.4 | 16.8 | 26.1 | 0.0 | 33.3 | 1.4 |
| Obstipation | 10.7 | 2.8 | 18.7 | 0.0 | 16.7 | 2.5 | 16.6 | 12.5 | 20.6 | 0.0 | 33.3 | 1.8 |
| Abdominal pain/cramps | 11.4 | 4.9 | 18.0 | 0.0 | 33.3 | 1.3 | 23.7 | 19.4 | 28.0 | 0.0 | 33.3 | 1.1 |
| Flatulence | 21.9 | 11.5 | 32.2 | 0.0 | 33.3 | 1.0 | 36.3 | 31.5 | 41.0 | 33.3 | 66.6 | 0.4 |
| Frequent urination | 23.9 | 13.7 | 34.2 | 16.7 | 33.3 | 0.9 | 31.6 | 26.9 | 36.4 | 33.3 | 66.6 | 0.6 |
| Muscle/joint cramps/pain (scale) | 30.9 | 24.0 | 37.8 | 33.3 | 33.3 | 0.0 | 28.6 | 25.2 | 31.9 | 25.8 | 28.7 | 0.7 |
| Pail/cold fingers/toes* | 20.8 | 6.4 | 35.2 | 33.3 | 33.3 | -0.5 | 27.0 | 18.2 | 35.9 | 0.0 | 66.6 | 1.0 |
| Tingling/numbness hands/feet | 12.5 | 5.2 | 19.8 | 0.0 | 33.3 | 1.4 | 27.2 | 22.7 | 31.7 | 33.3 | 33.3 | 0.9 |
| Problems finger dexterity | 17.2 | 7.8 | 26.6 | 0.0 | 33.3 | 1.5 | 12.0 | 8.8 | 15.2 | 0.0 | 33.3 | 2.0 |
| Back pain* | 37.5 | 6.1 | 68.8 | 33.3 | 66.6 | 0.4 | 25.1 | 18.2 | 32.0 | 33.3 | 33.3 | 1.0 |
| Fever/chills | 2.1 | 0.0 | 5.2 | 0.0 | 0.0 | 3.5 | 9.9 | 7.0 | 12.7 | 0.0 | 0.0 | 2.2 |
| Sweating excessively | 18.3 | 9.4 | 27.1 | 0.0 | 33.3 | 0.9 | 17.8 | 14.0 | 21.5 | 0.0 | 33.3 | 1.2 |
| Problems tolerating heat/cold | 16.1 | 7.8 | 24.4 | 0.0 | 33.3 | 1.0 | 20.3 | 16.3 | 24.3 | 0.0 | 33.3 | 1.3 |
| Hot flushes | 10.7 | 4.1 | 17.3 | 0.0 | 33.3 | 1.4 | 12.8 | 9.2 | 16.5 | 0.0 | 33.3 | 2.0 |
| Airway infections* | 0.0 | NA | NA | 0.0 | 0.0 | NA | 10.6 | 4.7 | 16.5 | 0.0 | 0.0 | 2.5 |
| Other infections* | 4.2 | 0.0 | 14.0 | 0.0 | 0.0 | 2.3 | 6.8 | 1.7 | 11.8 | 0.0 | 0.0 | 3.3 |
| Sore/enlarged nipples/breasts | 3.2 | 0.0 | 6.9 | 0.0 | 0.0 | 2.7 | 3.4 | 1.8 | 4.9 | 0.0 | 0.0 | 3.1 |
| Sexuality problems (scale) | 23.0 | 11.5 | 34.4 | 11.1 | 41.6 | 1.3 | 27.1 | 23.0 | 31.1 | 22.2 | 44.4 | 0.9 |
| Difficulty getting/maintaining erection | 11.9 | 0.0 | 24.1 | 0.0 | 25.0 | 1.5 | 23.2 | 16.3 | 30.2 | 0.0 | 33.3 | 1.2 |
| Dry vagina | 35.5 | 15.2 | 55.8 | 33.3 | 66.6 | 0.5 | 27.9 | 21.0 | 34.8 | 33.3 | 58.3 | 0.8 |

* At T4 as not available at T1. Abbreviations: CI: confidence interval; IQR: inter quartile range. Shaded cells indicate a skewness of ≥2.0. A skewness of <1.0 is seen as excellent, and <2.0 as acceptable [9].

# Our pilot data in the context of published literature

## Meta-analysis of severity of symptoms

In a systematic search in PubMed and Embase, up to February 2025 [10] we identified eight other studies that used EORTC-instruments in CML-patients, with mean severity data that we could compare to our data (Table 30). We excluded one Indian study from this comparison, as it’s mean symptom severities were only a fraction of those reported in other studies and therefore lacked credibility [11]. For example, they reported a mean diarrhoea severity of 1.4, compared to a range of 17.9-21.7 in other studies. Similarly, for dyspnoea, they reported a mean severity of 1.3, compared to a range of 22.3-27.1 in other studies. Five further studies had data that could not be compared to other data, because either: means could not be extracted from the article [12-14]; it was unclear for which symptoms means/medians were reported [15]; or linear transformation had not been performed [16].

Symptom severity could be compared for eight symptoms/scales, for dasatinib, imatinib and nilotinib as current treatments. If only the standard deviation was given, we calculated the 95% confidence interval with the webtool [www.openepi.com](http://www.openepi.com), with the population size set at 10,000, based on the z-test for sample sizes ≥30, and based on the t-test for sample sizes <30. For the 24 symptom-treatment combinations, there was no significant difference (defined as: overlapping 95%CIs of the unadjusted estimates) between our data and data from the literature in 28 combinations. In six symptom-treatment combinations there was a significant difference between our data and data from the literature, with differences between unadjusted estimates ranging from 6.5 to 21.3%. Notably, we could not compare our data to data collected with a validated questionnaire for most of the items in our questionnaire.

In a random effects meta-analysis, there was no statistical difference between TKI treatments for fatigue, pain and insomnia (Figure 1, Figure 3, and Figure 5), whereas:

- Patients on imatinib reported more severe nausea and/or vomiting compared to patients on dasatinib or nilotinib (unadjusted mean severity difference 5.4% and 7.9%, Figure 2)
- Patients on dasatinib and imatinib reported more severe dyspnoea than patients on nilotinib (unadjusted mean severity difference 8.8% and 11.4%, Figure 4)
- Patients on imatinib reported more severe appetite loss than patients on nilotinib (unadjusted mean severity difference 10.1%, Figure 6)
- Patients on dasatinib reported more severe constipation than patients on imatinib (unadjusted mean severity difference 12.6%, Figure 7)
- Patients on imatinib reported more severe diarrhoea than patients on nilotinib (unadjusted mean severity difference 17.1%, Figure 8)

Table 30 Comparison of mean symptom severity, measured with EORTC-items in patient groups ≥30 symptoms

| **Symptom, treatment** | **Study** | **Recruitment** | **Number of included patients** | **Mean (95%CI))** |
| --- | --- | --- | --- | --- |
| *Fatigue scale EORTC ** | | | | |
| D | Efficace [17] | Hospital-based | 94 | 29.1 (24.4-33.8) |
|  | Foulon [18] | Web-based | 66 | 60.3 (53.0-67.6) |
|  | This study | Web-based | 70 | 47.3 (40.5-54.2) |
| I | Ali [19] | Hospital-based | 84 | 30.3 (26.7-33.9) |
|  | Efficace [17] | Hospital-based | 94 | 34.9 (29.7-40.1) |
|  | Foulon [18] | Web-based | 146 | 49.2 (44.9-53.5) |
|  | Rashid [20] | Hospital-based | 101 | # 35.8 (31.4-40.2) |
|  | This study | Web-based | 84 | 40.0 (34.4-45.6) |
| N | Ali [19] | Hospital-based | 38 | 33.7 (27.6-39.8) |
|  | Efficace [21] | Hospital based | 124 | $ 19.1 (15.3-22.8) |
|  | Foulon [18] | Web-based | 84 | 48.5 (42.9-54.1) |
|  | Sacha [22] | Hospital-based | 177 | § 26.6 (23.6-29.6) |
|  | Nguyen [23] | Hospital-based | 121 | 28.3 (24.3-32.3) |
|  | This study | Web-based | 39 | 40.4 (33.0-47.8) |
| D, I, N, O | Efficace [24] | Unclear | 779 | 28.0 (27.2-28.8) |
| D, I, N | This study | Web-based | 193 | 42.7 (39.0-46.5) |
| B, D, I, N, P, O, TFR | Foulon [18] | Web-based | 377 | 50.0 (47.8-52.2) |
| B, D, I, N, P, TFR | This study | Web-based | 255 | 42.5 (39.2-45.7) |
| *Nausea/vomiting scale EORTC* | | | | |
| D | Efficace [17] | Hospital-based | 94 | 6.1 (4.0-8.2) |
|  | This study | Web-based | 70 | 9.5 (5.4-13.7) |
| I | Ali [19] | Hospital-based | 84 | 11.8 (9.3-14.3) |
|  | Efficace [17] | Hospital-based | 94 | 10.6 (7.2-14.0) |
|  | Rashid [20] | Hospital-based | 101 | # 13.4 (11.8-15.0) |
|  | This study | Web-based | 84 | 14.0 (10.4-17.6) |
| N | Ali [19] | Hospital-based | 38 | 10.1 (5.7-14.5) |
|  | Efficace [21] | Hospital based | 124 | $ 2.7 (1.3-4.1) |
|  | Nguyen [23] | Hospital-based | 121 | 7.2 (na) |
|  | Sacha [22] | Hospital-based | 177 | § 2.2 (1.3-3.1) |
|  | This study | Web-based | 39 | 6.2 (2.0-10.5) |
| D, I, N, O | Efficace [24] | Unclear | 779 | 6.2 (5.8-6.6) |
| D, I, N | This study | Web-based | 193 | 10.8 (8.5-13.2) |
| B, D, I, N, P, O, TFR | Foulon [18] | Web-based | 377 | 13.7 (12.1-15.3) |
| B, D, I, N, P, TFR | This study | Web-based | 255 | 9.7 (7.6-11.8) |
| *Pain* | | | | |
| D | Efficace [17] | Hospital-based | 94 | 14.7 (10.3-19.1) |
|  | This study | Web-based | 70 | 25.4 (17.9-33.0) |
| I | Ali [19]  Efficace [17] | Hospital-based  Hospital-based | 84  94 | 18.3 (13.6-23.0)  22.0 (17.4-26.6) |
|  | Rashid [20] | Hospital-based | 101 | # 28.7 (24.8-32.6) |
|  | This study | Web-based | 84 | 24.0 (17.3-30.7) |
| N | Ali [19]  Nguyen [23] | Hospital-based  Hospital-based | 38  121 | 32.0 (23.2-40.8)  21.1 (16.7-25.5) |
|  | Sacha [22] | Hospital-based | 177 | § 16.0 (13.0-19.0) |
|  | This study | Web-based | 39 | 18.7 (8.6-28.8) |
| *Dyspnoea* | | | | |
| D | Efficace [17] | Hospital-based | 94 | 21.4 (16.4-26.4) |
|  | This study | Web-based | 70 | 20.8 (14.3-27.3) |
| I | Ali [19]  Efficace [17] | Hospital-based  Hospital-based | 84  94 | 18.6 (13.6-23.6)  22.3 (17.0-27.6) |
|  | Rashid [20] | Hospital-based | 101 | # 26.7 (24.3-29.1) |
|  | This study | Web-based | 84 | 27.1 (20.3-33.9) |
| N | Ali [19]  Nguyen [23] | Hospital-based  Hospital-based | 38  121 | 15.9 (8.9-22.9)  14.3 (na) |
|  | Sacha [22] | Hospital-based | 177 | § 11.3 (8.5-14.1) |
|  | This study | Web-based | 39 | 14.1 (6.3-22.0) |
| D, I, N, O | Efficace [24] | Unclear | 779 | 17.0 (16.2-17.8) |
| D, I, N | This study | Web-based | 193 | 22.2 (18.1-26.3) |
| B, D, I, N, P, O, TFR | Foulon [18] | Web-based | 377 | 30.1 (27.7-32.8) |
| B, D, I, N, P, TFR | This study | Web-based | 255 | 21.3 (17.6-25.0) |
| *Insomnia* | | | | |
| D | Efficace [17] | Hospital-based | 94 | 20.4 (15.0-25.8) |
|  | This study | Web-based | 70 | 29.9 (21.3-38.6) |
| I | Ali [19]  Efficace [17] | Hospital-based  Hospital-based | 84  94 | 20.8 (15.9-25.7)  27.0 (20.9-33.1) |
|  | Rashid [20] | Hospital-based | 101 | # 15.7 (14.3-17.1) |
|  | This study | Web-based | 84 | 33.3 (25.5-41.1) |
| N | Ali [19]  Nguyen [23] | Hospital-based  Hospital-based | 38  121 | 17.3 (10.3-24.3)  22.9 (18.0-27.8) |
|  | Sacha [22] | Hospital-based | 177 | § 21.1 (17.9-24.3) |
|  | This study | Web-based | 39 | 28.8 (19.0-38.7) |
| *Appetite loss* | | | | |
| D | Efficace [17] | Hospital-based | 94 | 11.8 (7.0-16.6) |
|  | This study | Web-based | 70 | 10.7 (5.3-16.1) |
| I | Ali [19]  Efficace [17] | Hospital-based  Hospital-based | 84  94 | 15.2 (11.0-19.4)  12.8 (8.4-17.2) |
|  | Rashid [20] | Hospital-based | 101 | # 23.4 (20.6-26.2) |
|  | This study | Web-based | 84 | 17.1 (0.4-23.9) |
| N | Ali [19]  Efficace [21] | Hospital-based  Hospital based | 38  124 | 13.0 (6.8-19.2)  ^$^4.1 (1.4-6.8) |
|  | Nguyen [23] | Hospital-based | 121 | 17.1 (na) |
|  | Sacha [22] | Hospital-based | 177 | § 7.5 (5.2-9.8) |
|  | This study | Web-based | 39 | 7.1 (0.6-13.5) |
| D, I, N, O | Efficace [24] | Unclear | 779 | 10.0 (9.2-10.8) |
| D, I, N | This study | Web-based | 193 | 12.8 (9.0-16.5) |
| B, D, I, N, P, O, TFR | Foulon [18] | Web-based | 377 | 14.7 (12.8-16.6) |
| B, D, I, N, P, TFR | This study | Web-based | 255 | 11.6 (8.5-14.8) |
| *Constipation* | | | | |
| D | Efficace [17] | Hospital-based | 94 | 19.5 (14.7-24.3) |
|  | This study | Web-based | 70 | 22.6 (14.1-31.1) |
| I | Ali [19] | Hospital-based | 84 | 2.2 (0.4-4.0) |
|  | Efficace [17] | Hospital-based | 94 | 10.9 (6.9-14.9) |
|  | Rashid [20] | Hospital-based | 101 | # 9.3 (8.5-10.1) |
|  | This study | Web-based | 84 | 8.7 (4.4-12.9) |
| N | Ali [19]  Nguyen [23] | Hospital-based  Hospital-based | 38  121 | 26.0 (13.7-38.1)  11.9 (na) |
|  | Sacha [22] | Hospital-based | 177 | § 10.5 (7.6-13.4) |
|  | This study | Web-based | 39 | 18.7 (6.9-30.3) |
| D, I, N, O | Efficace [24] | Unclear | 779 | 9.9 (9.3-10.5) |
| D, I, N | This study | Web-based | 193 | 15.7 (11.4-20.0) |
| B, D, I, N, P, O, TFR | Foulon [18] | Web-based | 377 | 16.7 (14.5-18.9) |
| B, D, I, N, P, TFR | This study | Web-based | 255 | 15.2 (11.5-18.8) |
| *Diarrhoea* | | | | |
| D | Efficace [17] | Hospital-based | 94 | 8.8 (4.8-12.8) |
|  | This study | Web-based | 70 | 16.7 (10.1-23.2) |
| I | Ali [19]  Efficace [17] | Hospital-based  Hospital-based | 84  94 | 24.8 (17.8-31.8)  17.9 (13.4-22.4) |
|  | Rashid [20] | Hospital-based | 101 | # 18.8 (16.2-21.4) |
|  | This study | Web-based | 84 | 30.9 (22.3-39.5) |
| N | Ali [19]  Nguyen [23] | Hospital-based  Hospital-based | 38  121 | 8.7 (3.0-14.4)  5.5 (na) |
|  | Sacha [22] | Hospital-based | 177 | § 1.4 (0.5-2.3) |
|  | This study | Web-based | 39 | 7.3 (0.0-14.6) |
| D, I, N, O | Efficace [24] | Unclear | 779 | 12.0 (11.3-12.7) |
| D, I, N | This study | Web-based | 193 | 21.0 (16.2-25.8) |
| B, D, I, N, P, O, TFR | Foulon [18] | Web-based | 377 | 24.8 (22.3-27.3) |
| B, D, I, N, P, TFR | This study | Web-based | 255 | 19.7 (15.6-23.9) |

Grey shading indicates non-overlapping 95%CIs between studies. Red shaded areas indicate non-overlapping 95%CIs between studies, and a difference between unadjusted estimates of 15% or greater; * The EORTC QLQ-C30’s Fatigue scale consists of three items (needed rest, felt weak, tired), whilst our Fatigue (scale) consists of six items (tired, need to rest, lacked energy, lethargic, weak, drowsy). For this comparison the EORTC’s three-item Fatigue scale is used. $: after 24 months of treatment; #: after 12 months of treatment; §: at the sixth visit. Abbreviations: B: bosutinib, CI: confidence interval; D: dasatinib, I: imatinib, N: nilotinib, na: not available; O: other; P: ponatinib; SD: standard deviation; TFR: treatment-free remission

Figure 1 Meta-analysis of mean fatigue severity measured by EORTC instruments in ≥30 patients per treatment group

Figure 2 Meta-analysis of mean nausea/vomiting severity measured by EORTC instruments in ≥30 patients per treatment group

Figure 3 Meta-analysis of mean pain severity measured by EORTC instruments in ≥30 patients per treatment group

Figure 4 Meta-analysis of mean dyspnoea severity measured by EORTC instruments in ≥30 patients per treatment group

Figure 5 Meta-analysis of mean insomnia severity measured by EORTC instruments in ≥30 patients per treatment group

Figure 6 Meta-analysis of mean appetite loss severity measured by EORTC instruments in ≥30 patients per treatment group

Figure 7 Meta-analysis of mean constipation severity measured by EORTC instruments in ≥30 patients per treatment group

Figure 8 Meta-analysis of mean diarrhoea severity measured by EORTC instruments in ≥30 patients per treatment group

## TKI-treatments compared in other studies

In a systematic search in PubMed and Embase, from inception up to February 2025, we identified ten studies that compared symptom prevalence or mean symptom severity between current treatments, measured with validated patient reported measurement instruments in at least thirty patients (Table 31). Seven studies made comparisons between similar current treatments as we did, of which only one study by Efficace et al. adjusted for covariates [17]. This one study found that patients on dasatinib experienced less nausea/vomiting, pain and diarrhoea compared to imatinib, while patients on imatinib experienced less constipation. Comparing results from our study with this study, our results are similar with regard to diarrhoea and constipation. However, for nausea/vomiting and pain we found no difference between dasatinib and imatinib; while for headaches, skin problems, and hair loss we did find significant differences favouring imatinib. Possibly, differences in matching/covariates adjusted for, and/or population differences, are the cause of these differences. Efficace et al. matched on co-morbidity and previous CML-treatment for example, but did not adjust for dosage, as we did. Other differences we identified (Cognitive problems (scale), Swelling face/eyes, Feeling bloated, and Hot flushes) were not evaluated by Efficace et al.

Table 31 Significant differences in symptoms reported by patients, measured with validated instruments, in studies that included over 30 patients and made comparisons between TKI treatments

| **Article** | **Study type, setting** | **Current treatment** | **Questionnaires** | **Significant differences between current treatments** | **Adjusted for** |
| --- | --- | --- | --- | --- | --- |
| Abulafia 2020 [12] | Cross-sectional, nationwide, recruitment via e-mail patients’ organization | Dasatinib (n=45)  Imatinib (n=70)  Nilotinib (n=24) | EORTC QLQ-CML24 | Eight individual symptoms evaluated, three with significant differences in **mean symptom severity**:   - Less fatigue in imatinib (p=0.02) vs. dasatinib and nilotinib - Less pain in imatinib vs. nilotinib (p=0.01) - Less worry, stress, depression or nervousness in imatinib vs. nilotinib (p=0.01) | Not adjusted |
| Ali 2024 [19] | Single centre, observational | Bosutinib (n=39)  Imatinib (n=84)  Nilotinib (n=38) | EORTC QLQ-CML24 | Eight individual symptoms evaluated, three with significant differences in **mean symptom severity** (all p<0.05 after Bonferroni):   - Less severe pain in imatinib vs. nilotinib - Less severe constipation in imatinib versus bosutinib and nilotinib - More severe diarrhoea in bosutinib vs. imatinib and nilotinib, and in imatinib vs. nilotinib | Not adjusted |
| Bostan 2020 [15] | Cross-sectional, university medical centre | Dasatinib (n=30)  Original imatinib (n=17)  Generic imatinib (n=44)  Nilotinib (n=30) | EORTC QLQ-CML24, MDASI-CML | 28 Individual symptoms evaluated, three with significant differences in their **prevalence**:   - Less swelling of ankles, legs or around the eyes in dasatinib or nilotinib vs. imatinib (p=0.02) - Less rash/skin changes in imatinib (p=0.02) vs. dasatinib and nilotinib - Less appetite loss in original imatinib (p=0.03) vs. vs. dasatinib and nilotinib   The difference in **median severity** was evaluated for 8 individual symptoms, with no statistical differences between treatment groups | Multiple comparisons, not adjusted |
| Cho 2025 [13] | Retrospective cohort, single centre | Dasatinib (n=22)  Imatinib (n=53)  Nilotinib (n=67) | EORTC QLQ-C30 | Six individual symptoms for differences in **mean symptom severity**, of which 2 with significant differences:   - Nausea/vomiting was less severe in imatinib vs. nilotinib - Diarrhoea was more severe in imatinib and dasatinib vs. nilotinib | Not adjusted |
| Cortes 2019 [25] | RCT | Bosutinib (n=268)  Imatinib (n=268) | EQ-5D | Two symptoms evaluated (pain/discomfort and anxiety/depression), neither with significant differences in **repeated measures mixed-effect models** or **prevalence** at 12 months | Not adjusted (RCT) |
| Efficace 2020 [17] | Propensity matched multicentre case-control study | Dasatinib (n=94)  Imatinib (n=94) | EORTC QLQ-CML24 | Eight individual symptoms evaluated, four with significant **adjusted mean severity** differences:   - Less nausea/vomiting (p=0.02), pain, diarrhoea (p<0.01) in dasatinib - Less constipation in imatinib (p=0.03) | Matching on age, sex, living arrangement, comorbidity, ECOG-performance status, Sokal risk, and previous CML-treatment. Adjustment for education and months from treatment start |
| Réa 2023 [26] | RCT | Asciminib (n=157)  Bosutinib (n=76) | EQ-5D, MDASI-CML | 20 Individual symptoms evaluated, seven with significant differences in average change **of mean symptom severity** from baseline:  Favoured asciminib (as 95%CI did not include 0) for feeling drowsy, lack of appetite, dry mouth, nausea, vomiting, diarrhoea, and pain (p-values not given) | Not adjusted (RCT) |
| Uyanik [14] | Cohort, single centre | Imatinib (n=20)  Generic imatinib (n=22)  2^nd^-generation TKIs (n=10) | EORTC QLQ-C30, HADS | Eight individual symptoms evaluated (including anxiety and depression) in **mean severity**, one with a significant difference:   - Patients on generic imatinib reported more severe diarrhoea than patients on imatinib (p=0.012) | Not adjusted |
| Williams 2013 [27] | Cross-sectional, single centre | Bafetinib (n=1)  Bosutinib (n=6)  Dasatinib (n=34)  Imatinib (n=71)  Nilotinib (n=22)  Ponatinib (n=5)  Rebastinib (n=3)  Other (n=3)  No treatment (n=7) | MDASI-CML | Twenty individual symptoms tested, three with significant differences in **mean symptom severity**:   - Less severe diarrhoea (p<0.01), muscle soreness and cramping (p=0.02), swelling (p=0.04) in dasatinib and nilotinib vs. imatinib | Not adjusted |
| Zulbaran-Rojas 2018 [28] | Cohort study | Dasatinib (n=104)  Nilotinib (n=82)  Ponatinib (n=33) | MDASI-CML | Twenty individual symptoms tested, 11 with significant differences **in mean symptom severity**:   - Dasatinib lower disturbed sleep, diarrhoea (p<0.01) and oedema (p=0.02) than nilotinib - Dasatinib higher pain (p=0.03) than nilotinib - Dasatinib lower disturbed sleep vs. ponatinib (p=0.02) - Nilotinib lower malaise (p=0.01), oedema, pain (p<0.01), shortness of breath (p=0.01) than ponatinib - Dasatinib and nilotinib lower skin rash, muscle cramps, dry mouth (p<0.01) and distress (p<0.01 for dasatinib and p=0.03 for nilotinib) vs. ponatinib | Not adjusted |

Shaded cells indicate comparisons including between similar current treatments as in our study

# References

1.	Tong A, Sainsbury P, and Craig J (2007) Consolidated criteria for reporting qualitative research (coreq): A 32-item checklist for interviews and focus groups*.* Int J Qual Health Care 19: 349–57. <https://doi.org/10.1093/intqhc/mzm042>

2.	Baum F, MacDougall C, and Smith D (2006) Participatory action research*.* J Epidemiol Community Health 60: 854–7. <https://doi.org/10.1136/jech.2004.028662>

3.	von Elm E, Altman DG, Egger M, Pocock SJ, Gotzsche PC, Vandenbroucke JP, and Initiative S (2007) The strengthening the reporting of observational studies in epidemiology (strobe) statement: Guidelines for reporting observational studies*.* Lancet 370: 1453–7. <https://doi.org/10.1016/S0140-6736(07)61602-X>

4.	Terwee CB, Prinsen CAC, Chiarotto A, Westerman MJ, Patrick DL, Alonso J, Bouter LM, de Vet HCW, and Mokkink LB (2018) Cosmin methodology for evaluating the content validity of patient-reported outcome measures: A delphi study*.* Qual Life Res 27: 1159–1170. <https://doi.org/10.1007/s11136-018-1829-0>

5.	de Vet HC, Terwee C, Mokkink LB, and Knol DL, *Measurement in medicine: A practical guide*. Practical guides to biostatistics and epidemiology. 2011, Cambridge: Cambridge University Press.

6.	Revicki D, Hays RD, Cella D, and Sloan J (2008) Recommended methods for determining responsiveness and minimally important differences for patient-reported outcomes*.* J Clin Epidemiol 61: 102–9. <https://doi.org/10.1016/j.jclinepi.2007.03.012>

7.	Fayers PM, Aaronson NK, Bjordal K, Groenvold M, Curran D, and Bottomley A, *The eortc qlq-c30 scoring manual (3rd edition)*, E.Q.o.L. Group, Editor. 2001, European Organisation for Research and Treatment of Cancer: Brussels.

8.	Benjamin Y and Hochberg Y (1995) Controlling the false discovery rate: A practical and powerful approach to multiple testing*.* J R Soc Ser B (Methodol) 57: 289–300.

9.	Hair J, Hult G, Ringle C, and Sarstedt M, *A primer on partial least squares structural equation modeling (pls-sem) 3rd ed.* 2022, Thousand Oaks, CA: Sage.

10.	Smit Y, Scheuter P, Lange MPM, Janssen J, Posthuma EFM, Bekker CL, Hermens R, and Blijlevens NMA (2025) Patient-reported toxicity symptoms during tyrosine kinase inhibitor treatment in chronic myeloid leukemia: A systematic review and meta-analysis*.* Support Care Cancer 33: 446. <https://doi.org/10.1007/s00520-025-09451-4>

11.	Jain P, Das VN, Ranjan A, Chaudhary R, and Pandey K (2013) Comparative study for the efficacy, safety and quality of life in patients of chronic myeloid leukemia treated with imatinib or hydroxyurea*.* J Res Pharm Pract 2: 156–61. <https://doi.org/10.4103/2279-042X.128145>

12.	Shacham Abulafia A, Shemesh S, Rosenmann L, Berger T, Leader A, Sharf G, Raanani P, and Rozovski U (2020) Health-related quality of life in patients with chronic myeloid leukemia treated with first- versus second-generation tyrosine kinase inhibitors*.* J Clin Med 9. <https://doi.org/10.3390/jcm9113417>

13.	Cho HJ, Baek DW, Kim J, Jang YE, Lee Y, Moon JH, and Sohn SK (2025) Real-world experience in the management of chronic myeloid leukemia patients focused on tyrosine kinase inhibitors intolerance and health-related quality of life*.* Acta Haematol: 1–14. <https://doi.org/10.1159/000542562>

14.	Uyanik MS, Pamuk GE, Maden M, Merev E, Cevik G, Uyanik V, Umit EG, Demir AM, and Akker M (2017) The impact of new generic formulations of imatinib mesylate on general quality of life in chronic phase chronic myeloid leukaemia patients*.* Eur J Cancer Care (Engl) 26. <https://doi.org/10.1111/ecc.12423>

15.	Bostan H, Toptas T, Tanrikulu FP, Kut K, Arikan F, Yilmaz F, Atagunduz I, and Firatli-Tuglular T (2020) Quality of life and symptom burden with first- and second-generation tyrosine kinase inhibitors in patients with chronic-phase chronic myeloid leukemia*.* Clin Lymphoma Myeloma Leuk 20: 836–842. <https://doi.org/10.1016/j.clml.2020.08.009>

16.	Efficace F, Baccarani M, Breccia M, Saussele S, Abel G, Caocci G, Guilhot F, Cocks K, Naeem A, Sprangers M, Oerlemans S, Chie W, Castagnetti F, Bombaci F, Sharf G, Cardoni A, Noens L, Pallua S, Salvucci M, Nicolatou-Galitis O, Rosti G, and Mandelli F (2014) International development of an eortc questionnaire for assessing health-related quality of life in chronic myeloid leukemia patients: The eortc qlq-cml24*.* Qual Life Res 23: 825–36. <https://doi.org/10.1007/s11136-013-0523-5>

17.	Efficace F, Stagno F, Iurlo A, Breccia M, Cottone F, Bonifacio M, Abruzzese E, Castagnetti F, Caocci G, Crugnola M, Capodanno I, Martino B, Tiribelli M, Patriarca A, Gozzini A, Pregno P, Saussele S, Cascavilla N, Fozza C, Bergamaschi M, Binotto G, Vignetti M, and Rosti G (2020) Health-related quality of life of newly diagnosed chronic myeloid leukemia patients treated with first-line dasatinib versus imatinib therapy*.* Leukemia 34: 488–498. <https://doi.org/10.1038/s41375-019-0563-0>

18.	Foulon S, Cony-Makhoul P, Guerci-Bresler A, Daban M, Kapso R, Tubert-Bitter P, and Bonastre J (2021) Health state utility and quality of life measures in patients with chronic myeloid leukemia in france*.* Qual Life Res 30: 2021–2032. <https://doi.org/10.1007/s11136-021-02794-5>

19.	Ali H, Mohamed Z, and Abdullah D (2024) Health-related quality of life in chronic myeloid leukaemia patients receiving long-term therapy with different tyrosine kinase inhibitors in kurdistan region*.* Georgian Med News: 173–180.

20.	Rashid A and Albasry Z (2020) Assessing the quality of life in imatinib-treated chronic myeloid leukemia patients: A cross sectional descriptive study*.* Int J Pharmaceut Res 12: 2784–91.

21.	Efficace F, Castagnetti F, Martino B, Breccia M, D'Adda M, Angelucci E, Stagno F, Cottone F, Malato A, Trabacchi E, Capalbo SF, Gobbi M, Visani G, Salvucci M, Capodanno I, Tosi P, Tiribelli M, Scortechini AR, Levato L, Maino E, Binotto G, Gugliotta G, Vignetti M, Baccarani M, and Rosti G (2018) Health-related quality of life in patients with chronic myeloid leukemia receiving first-line therapy with nilotinib*.* Cancer 124: 2228–2237. <https://doi.org/10.1002/cncr.31323>

22.	Sacha T, Gora-Tybor J, Wasak-Szulkowska E, Kyrcz-Krzemien S, Medras E, Becht R, Bober G, Kotowska A, Waclaw J, and Hellmann A (2017) Quality of life and adherence to therapy in patients with chronic myeloid leukemia treated with nilotinib as a second-line therapy: A multicenter prospective observational study*.* Clin Lymphoma Myeloma Leuk 17: 283–295. <https://doi.org/10.1016/j.clml.2017.01.001>

23.	Nguyen CTT, Nguyen BT, Nguyen TTT, Petrelli F, Scuri S, and Grappasonni I (2022) Quality of life among chronic myeloid leukemia patients in the second-line treatment with nilotinib and influential factors*.* Qual Life Res 31: 733–743. <https://doi.org/10.1007/s11136-021-02952-9>

24.	Efficace F, Iurlo A, Patriarca A, Stagno F, Bee PC, Ector G, Capodanno I, Elena C, Bonifacio M, Blijlevens NMA, Caocci G, Wan C, Abruzzese E, Breccia M, Cottone F, Okumura I, Oerlemans S, Cascavilla N, Albano F, Kota V, Sztankay M, Miggiano MC, Saussele S, Di Renzo N, Sora F, Castagnetti F, Baccarani M, Vignetti M, and Rosti G (2021) Validation and reference values of the eortc qlq-cml24 questionnaire to assess health-related quality of life in patients with chronic myeloid leukemia*.* Leuk Lymphoma 62: 669–678. <https://doi.org/10.1080/10428194.2020.1838509>

25.	Cortes JE, Gambacorti-Passerini C, Deininger MW, Mauro MJ, Chuah C, Kim DW, Milojkovic D, le Coutre P, Garcia-Gutierrez V, Crescenzo R, Mamolo C, Reisman A, Hochhaus A, Brummendorf TH, and Investigators BS (2019) Patient-reported outcomes in the phase 3 bfore trial of bosutinib versus imatinib for newly diagnosed chronic phase chronic myeloid leukemia*.* J Cancer Res Clin Oncol 145: 1589–1599. <https://doi.org/10.1007/s00432-019-02894-3>

26.	Rea D, Boquimpani C, Mauro MJ, Minami Y, Allepuz A, Maheshwari VK, D'Alessio D, Wu Y, Lawrance R, Narbutas S, Sharf G, and Hochhaus A (2023) Health-related quality of life of patients with resistant/intolerant chronic phase chronic myeloid leukemia treated with asciminib or bosutinib in the phase 3 ascembl trial*.* Leukemia 37: 1060–1067. <https://doi.org/10.1038/s41375-023-01888-y>

27.	Williams LA, Garcia Gonzalez AG, Ault P, Mendoza TR, Sailors ML, Williams JL, Huang F, Nazha A, Kantarjian HM, Cleeland CS, and Cortes JE (2013) Measuring the symptom burden associated with the treatment of chronic myeloid leukemia*.* Blood 122: 641–7. <https://doi.org/10.1182/blood-2013-01-477687>

28.	Zulbaran-Rojas A, Lin HK, Shi Q, Williams LA, George B, Garcia-Manero G, Jabbour E, O'Brien S, Ravandi F, Wierda W, Estrov Z, Borthakur G, Kadia T, Cleeland C, Cortes JE, and Kantarjian H (2018) A prospective analysis of symptom burden for patients with chronic myeloid leukemia in chronic phase treated with frontline second- and third-generation tyrosine kinase inhibitors*.* Cancer Med 7: 5457–5469. <https://doi.org/10.1002/cam4.1808>
